# Multidisciplinary intervention in frail elderly patients with cardiovascular diseases: a multicentre, single-blinded, randomised controlled trial

**DOI:** 10.1038/s41467-026-77277-7

**Published:** 2026-08-31

**Authors:** Ke Chai, Chen Meng, Liwei Ji, Yujia Liu, Zhao Ma, Linlin Qiao, Lijuan Wang, Di Guo, Ning Sun, Lingling Cui, Simin Yao, Peipei Zheng, Xuelu Zhu, Wei Dong, Ping Zeng, Yao Lu, Ping Zhang, Guobin Miao, Yi Li, Jiefu Yang, Hua Wang

**Affiliations:** 1 Department of Cardiology, Beijing Hospital, National Centre for Gerontology; National Clinical Research Centre for Gerontology; The Key Laboratory of Geriatrics of NHC; Institute of Geriatric Medicine, Chinese Academy of Medical Sciences, Beijing, China; 2 Department of Geriatric Medicine, Beijing Hospital, National Centre for Gerontology; National Clinical Research Centre for Gerontology; The Key Laboratory of Geriatrics of NHC; Institute of Geriatric Medicine, Chinese Academy of Medical Sciences, Beijing, China; 3 Department of Pharmacy, Beijing Hospital, National Centre for Gerontology; National Clinical Research Centre for Gerontology; The Key Laboratory of Geriatrics of NHC; Institute of Geriatric Medicine, Chinese Academy of Medical Sciences, Beijing, China; 4 Department of Rehabilitation Medicine, Beijing Hospital, National Centre for Gerontology; National Clinical Research Centre for Gerontology; The Key Laboratory of Geriatrics of NHC; Institute of Geriatric Medicine, Chinese Academy of Medical Sciences, Beijing, China; 5 Department of Traditional Chinese Medicine, Beijing Hospital, National Centre for Gerontology; National Clinical Research Centre for Gerontology; The Key Laboratory of Geriatrics of NHC; Institute of Geriatric Medicine, Chinese Academy of Medical Sciences, Beijing, China; 6 Department of Nutrition, Beijing Hospital, National Centre for Gerontology; National Clinical Research Centre for Gerontology; The Key Laboratory of Geriatrics of NHC; Institute of Geriatric Medicine, Chinese Academy of Medical Sciences, Beijing, China; 7 Department of Cardiology, The First Hospital of Shanxi Medical University, Taiyuan, Shanxi China; 8 Department of Cardiology, The First Affiliated Hospital of Zhengzhou University, Zhengzhou, China; 9 Department of Biostatistics, Clinical Research Institute, Institute of Advanced Clinical Medicine, Peking University, Beijing, China; 10 Senior Department of Cardiology, The Sixth Medical Centre of PLA General Hospital, Beijing, China; 11 The Key Laboratory of Geriatrics, Beijing Institute of Geriatrics, Institute of Geriatric Medicine, Chinese Academy of Medical Sciences, Beijing Hospital, National Centre for Gerontology, Beijing, China; 12 Clinical Research Institute, Institute of Advanced Clinical Medicine, Peking University, Beijing, China; 13 Beijing Tsinghua Changgung Hospital, Beijing, China; 14 Department of Emergency, Emergency General Hospital, Beijing, China

**Keywords:** Cardiovascular diseases, Quality of life, Geriatrics

## Abstract

Evidence supporting multidisciplinary interventions for frailty in elderly cardiovascular inpatients remains limited. In this completed multicentre, single-blinded, randomised controlled trial (ChiCTR1900022623), 333 frail inpatients aged ≥ 70 years with cardiovascular diseases were randomly assigned to RENAP (Rehabilitation exercises, patient Education, Nutritional guidance, Acupoint massage, and Polypharmacy management) programme (*n* = 166) or usual care (*n* = 167). The trial was supported by grants from the Beijing Municipal Science and Technology Commission and the Chinese Academy of Medical Sciences. The primary outcomes were changes from baseline to 12 months in Fried Frailty Phenotype (FFP) and Short Physical Performance Battery (SPPB) scores at one year. RENAP significantly improved FFP score [t(993) = −4.79; *P* < 0.001; adjusted mean difference −0.79; 95% CI: −1.11 to −0.46] and SPPB score [t(993) = 4.06; *P* < 0.001; adjusted mean difference 2.02; 95% CI: 1.04 to 2.99]. No treatment-related serious adverse events were observed during the study. These findings suggest that a hospital-initiated multidisciplinary programme may improve frailty and physical function in frail elderly adults with cardiovascular diseases. The single-city setting and loss to follow-up may limit generalisability and warrant confirmation in broader healthcare settings.

## Abstract (web summary)

A hospital-initiated multidisciplinary program was associated with improved frailty and physical function and lower readmission risk in frail elderly patients with cardiovascular diseases, although larger studies are needed to confirm these.

## Introduction

By 2050, the global elderly population is projected to reach two billion, presenting profound challenges to healthcare systems worldwide^1^. Frailty, defined by the deterioration of multiple physiological systems and an increased susceptibility to stressors^2^, has emerged as a critical global health issue with significant influences for clinical practice and public health^3^. Importantly, frailty is not an irreversible condition, previous studies showed there were effective and cost-efficient interventions to reverse frailty and enhance the quality of life for elderly individuals with frailty^4^.

Frailty and cardiovascular diseases (CVD) are closely interconnected, sharing common pathological mechanisms such as chronic inflammation and oxidative stress, which mutually exacerbate each other^4^. A pivotal study involving over three million U.S. veterans revealed a strong correlation between the severity of frailty and cardiovascular mortality, independent of other cardiovascular conditions, highlighting the unmet need for clinical evidence showing efficacy of relieving frailty by various management strategies focusing on frailty screening and intervention by assessing clinical outcome among patients with CVD^5^.

In the setting of global aged population, healthcare providers increasingly encounter elderly adults presenting with both frailty and CVD. Physical inactivity, malnutrition are major components of frailty, which are associated with further poorer adverse outcomes among patients with CVD^4,6^. These challenges highlight the urgent need for clinical trials designed to prevent, mitigate or even reverse frailty within the context of CVD with targeted interventions^6^.

Emerging evidence supports the efficacy of tailored cardiac rehabilitation programmes, in improving frailty and physical function among patients with heart failure^7^. The relationship between frailty status at the onset of cardiac rehabilitation and long-term adverse outcomes is well-documented, with improvements in frailty linked to delayed hospitalisations and improved survival^8^. Many previous studies were conducted in community settings and the specific components and intensity of these programmes vary, creating a need for a standardised, reproducible, and comprehensive model focusing on the high-risk population of frail elderly inpatients with CVD. Acupoint massage, derived from traditional Chinese medicine (TCM), is recognized as an effective complementary therapy for insomnia symptoms and constipation, which contributes to a higher quality of life for elderly CVD patients^9,10^. Building on established interventions and our clinical experience, we developed a comprehensive, in-hospital initiated, multidisciplinary programme-RENAP (Rehabilitation, Education, Nutritional guidance, Acupoint massage and Polypharmacy management). To evaluate its efficacy, we conducted a multicentre randomised controlled trial across three cardiovascular departments in Beijing^11^. This study aimed to determine whether the RENAP intervention improved frailty status and physical function in frail elderly patients with CVD and to explore its potential effects on clinical outcomes.

## Results

### Baseline patient characteristics

**Fig. 1:**
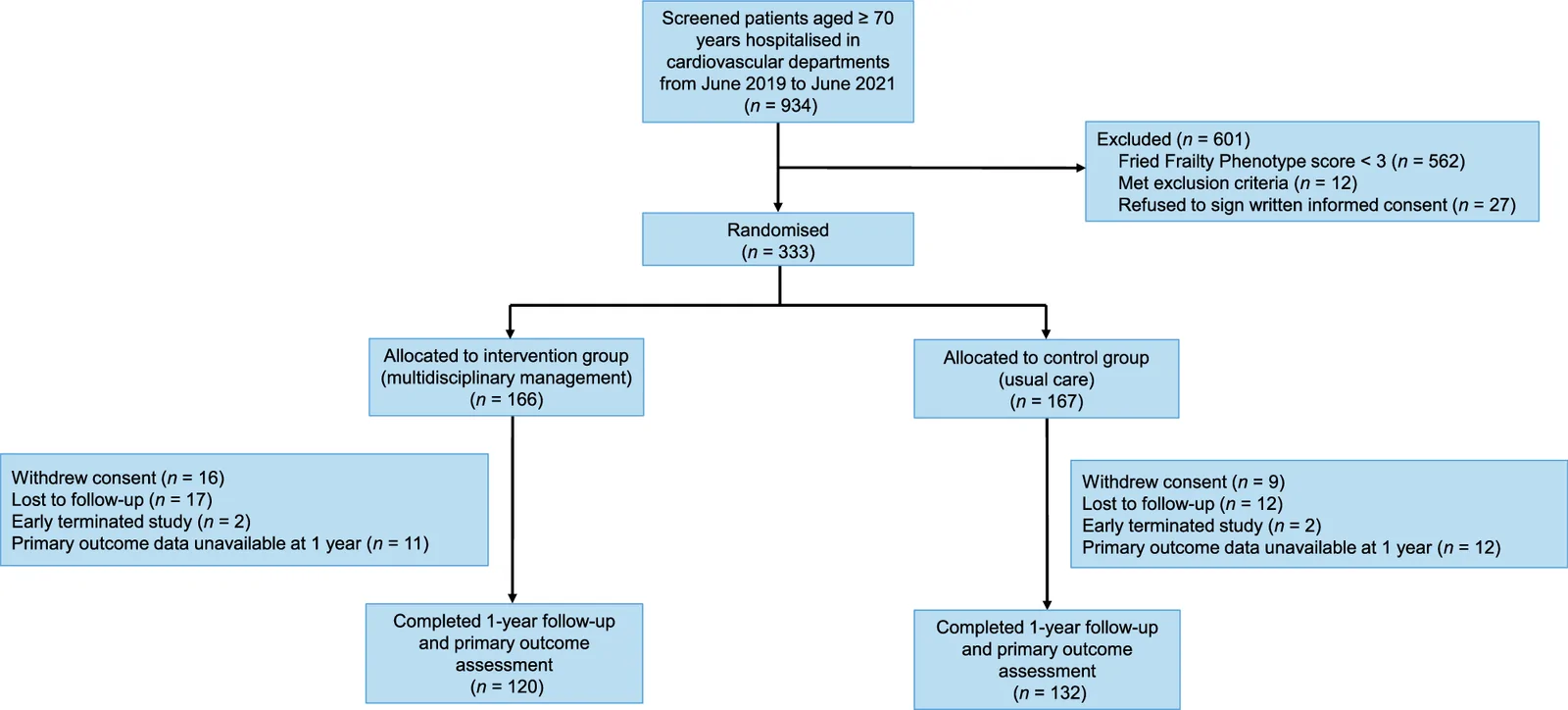
Flow of participants through the study. This figure shows the CONSORT diagram for participant flow throughout the study. Out of 934 screened patients aged 70 years or older, 333 participants were randomised into either the intervention group (*n* = 166) receiving multidisciplinary management or the control group (*n* = 167) receiving usual care. The diagram details the exclusion criteria, withdrawal reasons, and follow-up outcomes, illustrating participant retention and completion rates for the primary outcomes.

Figure 1 illustrates the participant flow through the trial. Between June 2019 and June 2021, 333 frail elderly patients were enrolled, with 166 assigned to the intervention group and 167 to the control group. Baseline characteristics are shown in Table 1. The mean age was 79.5 ± 5.4 years, with 42.6% male. 25 patients withdrew consent, 29 were lost to follow-up, 4 terminated the study early and 23 had unavailable primary outcome data at one year. Finally, 252 frail elderly patients completed the one-year primary outcome assessment, including 120 (72.3%) in the intervention group and 132 (79.0%) in the control group (Supplementary Table 1). The characteristics of participants who completed versus those lost to follow-up and those lost to follow-up in the intervention versus control groups are compared in Supplementary Tables 2 and 3, respectively.

**Table 1:** Baseline patient characteristics

	Control group (*n* = 167)	Intervention group (*n* = 166)
Demographic characteristics		
Age, years	79.2 ± 5.4	79.9 ± 5.5
Male, %	69 (41.3)	73 (44.0)
BMI, kg/m^2^	24.7 ± 3.3	25.3 ± 3.7
Widowed, %	20 (12.4)	28 (17.6)
Living alone, %	24 (15.0)	23 (14.5)
Mental work, %	109 (67.7)	106 (66.7)
History of smoking, %	38 (23.6)	44 (27.7)
Education level		
Primary school or below, %	28 (17.3)	23 (14.5)
Junior high school, %	41 (25.3)	47 (29.6)
Senior high school, %	39 (24.1)	36 (22.6)
Junior college or above, %	54 (33.3)	53 (33.3)
Clinical diseases		
Hypertension, %	139 (84.2)	139 (84.8)
Coronary artery disease, %	120 (72.7)	113 (68.9)
Atrial fibrillation or flutter, %	46 (27.9)	44 (27.0)
Heart failure, %	46 (27.9)	43 (26.2)
Diabetes mellitus, %	66 (40.0)	65 (39.6)
Chronic kidney disease, %	29 (17.6)	20 (12.2)
Malignancy, %	18 (10.9)	17 (10.4)
Polypharmacy (≥ 5 medications), %	122 (73.1)	121 (72.9)
Comprehensive geriatric assessments		
FFP score	3.5 ± 0.6	3.5 ± 0.6
Handgrip strength, kg	18.4 ± 6.4	18.7 ± 6.9
Gait speed, m/s	0.6 ± 0.2	0.6 ± 0.2
Unintentional weight loss, %	41 (24.6)	45 (27.1)
Physical exhaustion, %	145 (86.8)	143 (86.1)
Low physical activity, %	128 (76.6)	122 (73.5)
SPPB	6.2 ± 2.6	6.0 ± 2.8
BADL	5.5 ± 1.3	5.5 ± 1.2
IADL	6.2 ± 2.2	6.3 ± 2.2
CFS	4.8 ± 0.8	4.8 ± 0.8
HADS-A	2.0 ± 2.1	2.7 ± 3.7
NRS2002	1.7 ± 0.9	1.8 ± 1.0
PSQI	9.0 ± 3.8	9.0 ± 4.3
Admission laboratory values		
Glycosylated hemoglobin, %	6.4 ± 1.0	6.7 ± 1.2
White blood cell, ×10^9^/L	6.0 ± 2.1	6.1 ± 1.8
Albumin, g/L	38.8 ± 3.5	39.0 ± 3.5
Creatine, umol/L	92.3 ± 53.5	82.8 ± 29.4
Total cholesterol, mmol/L	3.7 ± 0.9	3.7 ± 0.9
Triglyceride, mmol/L	1.2 ± 0.7	1.3 ± 0.8
hs-CRP, mg/L	2.0 ± 4.5	3.7 ± 5.9
NT-proBNP, pg/mL	448.3 (148.8,1201.5)	392.6 (151.4, 1152.0)
LVEF, %	59.4 ± 8.6	58.9 ± 9.3

Values are mean ± SD, *n* (%), or median (interquartile range). Percentages were calculated using the number of participants with available data for each variable as the denominator, unless otherwise specified. *n* shown in the column headings represent the total number of randomised participants.

*BADL* Basic Activities of Daily Living, *BMI* body mass index, *CFS* Clinical Frailty Scale, *FFP* Fried Frailty Phenotypem, *hs-CRP* high-sensitive C-reactive protein, *HADS-A* Hospital Anxiety and Depression Scale-anxiety, *IADL* Instrumental Activities of Daily Living, *LVEF* left ventricular ejection fraction, *NRS2002* Nutrition Risk Screening-2002, *NT-proBNP* N-terminal pro-B-type natriuretic peptide, *PSQI* Pittsburgh Sleep Quality Index, *SPPB* Short Physical Performance Battery.

### Primary outcomes

**Fig. 2:**
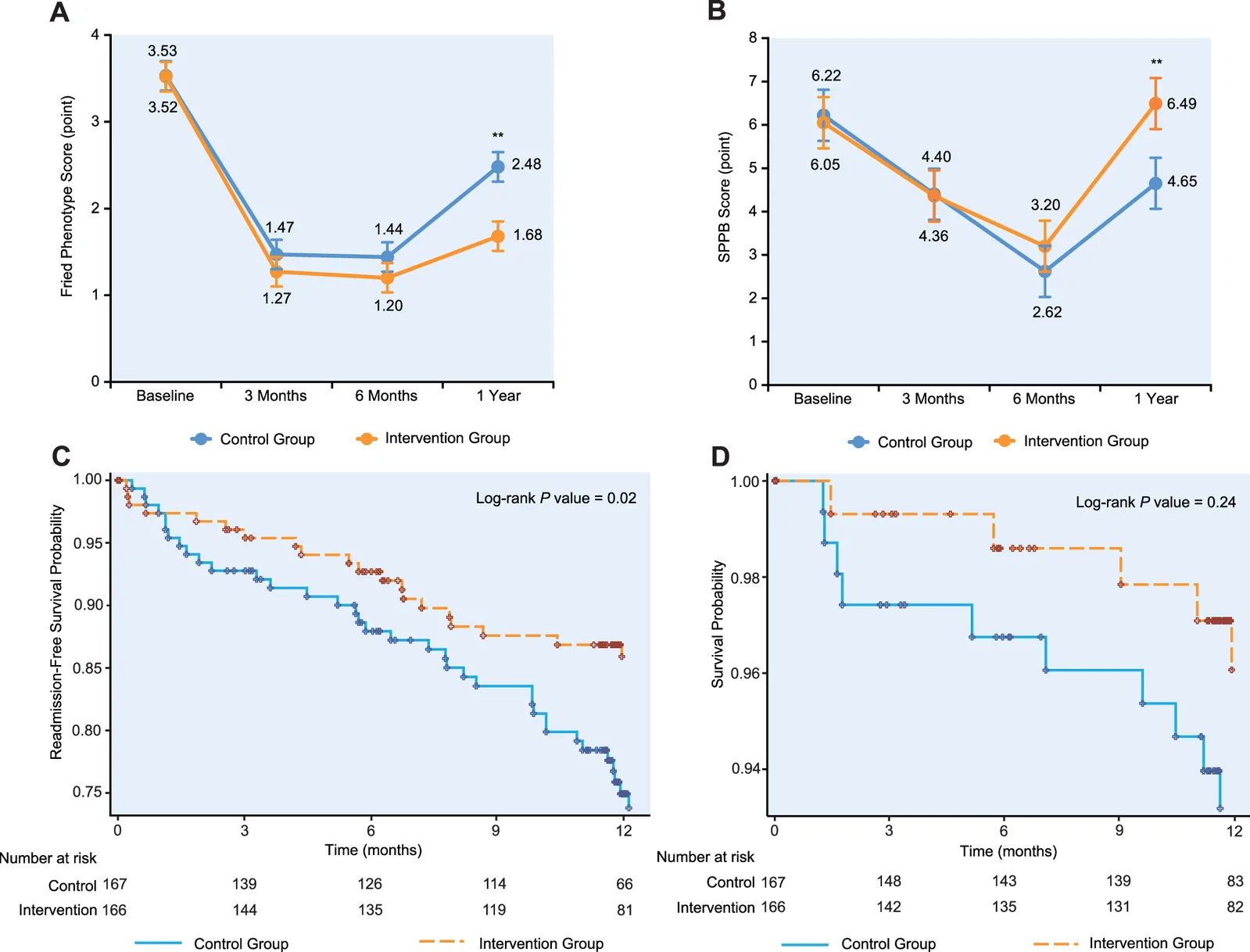
Impact of multidisciplinary intervention on frailty, physical function, readmission and mortality. **A**, **B** show changes in Fried Frailty Phenotype (FFP) and Short Physical Performance Battery (SPPB) scores from baseline to 12 months. Data are presented as least-square mean ± 95% confidence interval estimated from two-sided mixed-effects models using all available data from the intention-to-treat population (*n* = 333 participants). The between-group adjusted mean differences in FFP and SPPB at 12 months were −0.79 [*t* (993) = −4.79; *P* < 0.001; 95% CI: −1.11 to −0.46] and 2.02 [*t* (993) = 4.06; *P* < 0.001; 95% CI: 1.04–2.99]. **C**, **D** show Kaplan–Meier curves for all-cause readmission and mortality, compared using two-sided log-rank tests [*χ*²(1) = 5.44, *P* = 0.020 for readmission; *χ*²(1) = 1.40, *P* = 0.237 for mortality]. The number of participants at risk at each time point is shown in the tables below the curves. For all panels, blue and orange lines represent the control and intervention groups, respectively, and no adjustments were made for multiple comparisons. Source data are provided as a Source Data file. ** *P* < 0.001.

In the Intention-to-Treat (ITT) analysis, at the 12-month follow-up, the intervention group showed significant improvements in both Fried Frailty Phenotype (FFP) and Short Physical Performance Battery (SPPB) scores compared with baseline (Table 2). The mean FFP score decreased by 1.8 points in the intervention group compared to a decrease of 1.1 points in the control group, resulting in a between-group adjusted mean difference of −0.79 points [*t*(993) = −4.79; *P* < 0.001; 95% confidence interval (CI): −1.11 to −0.46]. Additionally, the mean SPPB score improved by 0.4 points in the intervention group, while it decreased by 1.6 points in the control group, leading to a between-group adjusted mean difference of 2.02 points [*t*(993) = 4.06; *P* < 0.001; 95% CI: 1.04–2.99]. Fig. 2A, B summarize the changes in FFP and SPPB scores over the study period. In the Per-Protocol Set (PPS) analysis (*n* = 120 for RENAP, *n* = 132 for control), the intervention group showed significant improvements in both FFP and SPPB scores (Supplementary Table 4 and Supplementary Fig. 1A, B). The between-group adjusted difference in FFP and SPPB at 12 months was −0.90 [*t*(750) = −4.67; *P* < 0.001; 95% CI: −1.27 to −0.52] and 2.78 [*t*(750) = 4.85; *P* < 0.001; 95% CI: 1.65–3.90] in the PPS analysis, respectively.

**Table 2:** Estimated between-group adjusted mean differences in change from baseline to 12 months

Outcome	Baseline		1-year follow-up		Least-square mean change from baseline to 1 year		Estimated adjusted difference in mean change at 1-year (95% CI)	Statistic (degrees of freedom)	*P* value
	Control group	Intervention group	Control group	Intervention group	Control group	Intervention group			
FFP score	3.5 ± 0.6	3.5 ± 0.6	2.5 ± 1.4	1.7 ± 1.3	−1.1 ± 0.1	−1.8 ± 0.1	−0.79 (−1.11 to −0.46)	*t*(993) = −4.79	<0.001
SPPB	6.2 ± 2.6	6.0 ± 2.8	4.6 ± 3.0	6.5 ± 4.0	−1.6 ± 0.4	0.4 ± 0.4	2.02 (1.04 to 2.99)	*t*(993) = 4.06	<0.001
IADL	6.2 ± 2.2	6.3 ± 2.2	5.4 ± 2.5	6.5 ± 2.2	−1.0 ± 0.2	0.2 ± 0.2	1.18 (0.69 to 1.67)	*t*(611) = 4.75	<0.001
CFS	4.8 ± 0.8	4.8 ± 0.8	5.0 ± 1.1	4.3 ± 1.2	0.2 ± 0.1	−0.4 ± 0.1	−0.67 (−0.90 to −0.44)	*t*(610) = −5.70	<0.001
HADS-A	2.0 ± 2.1	2.7 ± 3.7	2.9 ± 2.8	2.3 ± 2.8	0.9 ± 0.3	−0.4 ± 0.3	−1.31 (−2.18 to −0.43)	*t*(415) = −2.93	0.004

Values are shown as the mean ± standard deviation, otherwise indicated. Least-square mean changes from baseline to 1-year follow-up and estimated adjusted differences at 1-year were obtained from two-sided mixed-effects models. Least-square mean changes are presented as estimated change ± SE, and estimated differences are presented with 95% CIs. All reported *P* values for secondary outcomes are nominal and have not been adjusted for multiple comparisons.

*CFS* Clinical Frailty Scale, *CI* confidence interval, *FFP* Fried Frailty Phenotype, *HADS-A* Hospital Anxiety and Depression Scale-anxiety, *IADL* Instrumental Activities of Daily Living, *SPPB* Short Physical Performance Battery.

### Secondary and exploratory outcomes

The intervention group also demonstrated significant improvements in some secondary and non-pre-specified exploratory outcomes (Table 2 and Supplementary Table 5). The mean Clinical Frailty Scale (CFS) score decreased by 0.4 points in the intervention group compared to an increase of 0.2 points in the control group, resulting in a between-group adjusted mean difference of −0.67 points [*t*(610) = −5.70; *P* < 0.001; 95% CI: −0.90 to −0.44]. The mean Instrumental Activities of Daily Living (IADL) score improved by 0.2 points in the intervention group, while it decreased by 1.0 points in the control group, yielding a between-group adjusted mean difference of 1.18 points [*t*(611) = 4.75; *P* < 0.001; 95% CI: 0.69–1.67]. The mean Hospital Anxiety and Depression Scale-Anxiety (HADS-A) score decreased by 0.4 points in the intervention group, while it increased by 0.9 points in the control group. This resulted in a between-group adjusted mean difference of −1.31 points [*t*(415) = −2.93; *P* = 0.004; 95% CI: −2.18 to −0.43]. In the PPS analysis, the intervention group showed significant improvements in the CFS, IADL, and HADS-A score (Supplementary Table 4).

During the 1-year follow-up, 20 patients (12.0%) in the intervention group and 36 patients (21.6%) in the control group experienced all-cause readmission [*χ*²(1) = 5.44; *P* = 0.020 in the log-rank test, Fig. 2C]. Cox regression analysis (Supplementary Table 6) revealed that the intervention group had a significantly lower one-year all-cause readmission risk compared to the control group [Wald *χ*²(1) = 4.75; *P* = 0.029; hazard ratio (HR) = 0.53, 95% CI: 0.30–0.94]. In the PPS analysis, the intervention group also demonstrated a lower all-cause readmission rate (Supplementary Fig. 1C).

Regarding mortality during the 1-year follow-up, 5 patients (3.0%) in the intervention group and 10 patients (6.0%) in the control group experienced all-cause mortality [*χ*²(1) = 1.40; *P* = 0.237 in the log-rank test, Fig. 2D]. Cox regression analysis yielded a HR of 0.53 [Wald *χ*²(1) = 1.14; *P* = 0.286; 95% CI: 0.16–1.70; Supplementary Table 6) and the PPS analysis also did not indicate statistical significance (Supplementary Fig. 1D).

Regarding safety, the number of new falls occurring during each follow-up period is shown in Supplementary Table 7. At each time point, there were no statistically significant differences between the two groups [0–3 months: *χ*²(1) = 0.133, *P* = 0.715; 3–6 months: *χ*²(1) = 1.100, *P* = 0.294; 6–12 months: *χ*²(1) = 1.018, *P* = 0.313]. Fall-related hospitalisations occurred in one participant in the intervention group and in three participants in the control group. All fall-related hospitalisations were non-fatal.

### Subgroup analysis

**Fig. 3:**
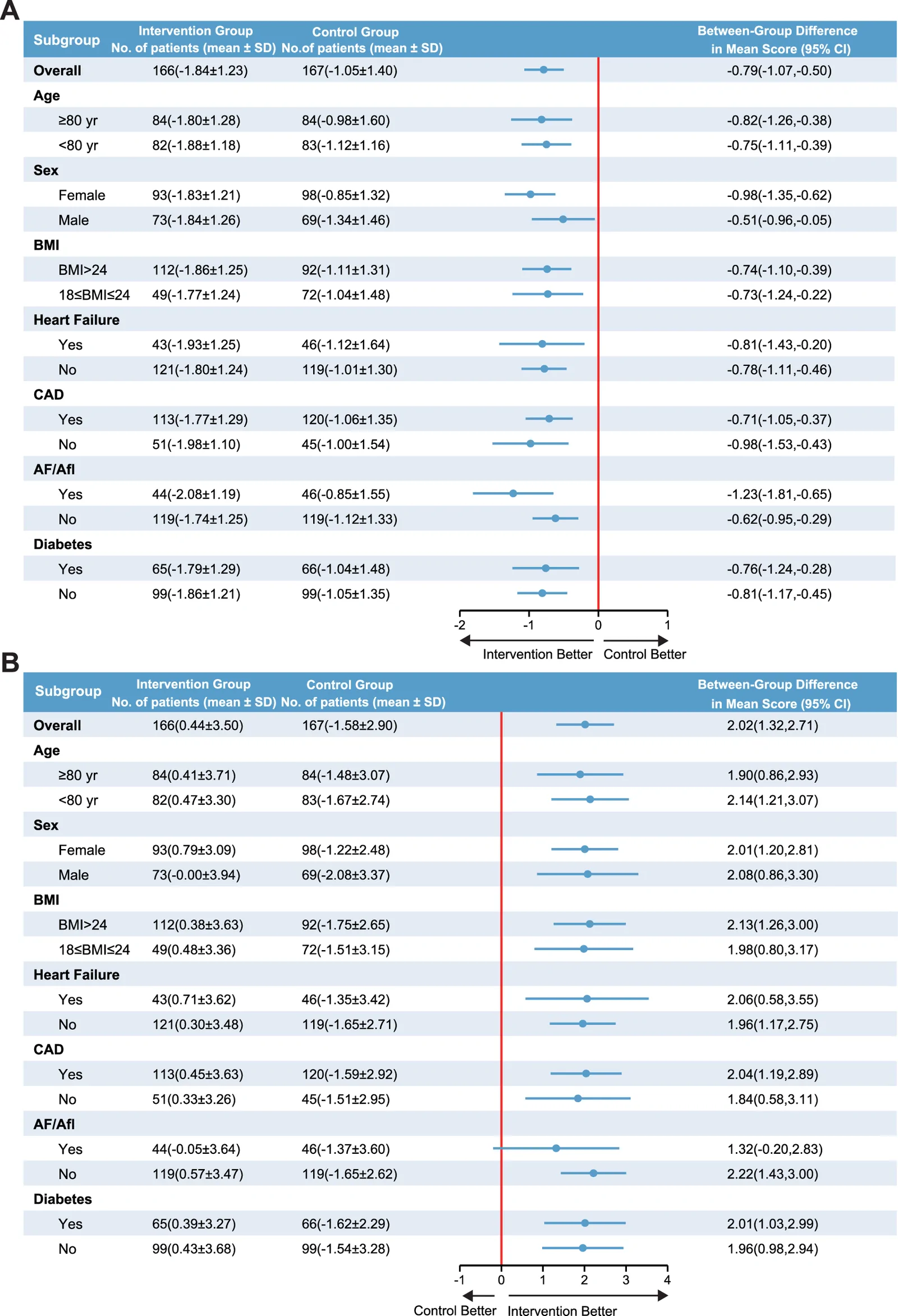
Subgroup analysis of multidisciplinary intervention effects on frailty and physical function. Forest plots show the effects of the multidisciplinary intervention on Fried Frailty Phenotype (FFP) and Short Physical Performance Battery (SPPB) scores across pre-specified subgroups. Between-group comparisons were performed using two-sample *t*-tests or Wilcoxon rank-sum tests, as appropriate. **A** shows the between-group differences in FFP score, and **B** shows those in SPPB scores. Subgroups included age, sex, body mass index (BMI), heart failure, coronary artery disease (CAD), atrial fibrillation/flutter (AF/Afl), and diabetes. Dots represent point estimates of the between-group differences; horizontal lines indicate 95% confidence intervals. The solid vertical line at 0 indicates no difference between groups. All subgroup analyses were exploratory and were not adjusted for multiple comparisons. Source data are provided as a Source Data file.

Subgroup analyses were presented in Fig. 3 (Fig. 3A for FFP and Fig. 3B for SPPB). For all subgroups, the point estimates for the between-group differences favoured the RENAP group for both FFP and SPPB. For all-cause readmission, the hazard ratios favoured the intervention group across most subgroups (Supplementary Fig. 2).

## Discussion

This randomised controlled trial demonstrated that a multidisciplinary intervention significantly improved frailty status and physical function in frail elderly patients with CVD, as reflected by improvements in the pre-specified primary outcomes of FFP and SPPB scores. The RENAP programme was also associated with a lower risk of all-cause readmission, although this secondary clinical outcome should be interpreted cautiously. Improvements in several secondary and exploratory measures, including CFS, IADL and HADS-A, further support the potential benefits of a multidisciplinary approach for this vulnerable population. Our study’s findings, particularly the significant improvements in frailty and functional outcomes, align with previous research on multidisciplinary interventions for frail elderly patients^12,13^. However, our study introduces several novel elements in comparison with earlier works.

While Fairhall et al. demonstrated the efficacy of a multidisciplinary management model in enhancing SPPB scores, our RENAP approach uniquely integrates a broader set of interventions, including acupoint massage to address insomnia and constipation, patient education, and nutritional guidance^12^. These added intervention items were designed to address a broader range of geriatric issues. This holistic approach underscores the importance of addressing both physical and mental health in frail elderly patients with CVD.

The SPRINTT project by Bernabei et al. demonstrated the efficacy of physical activity and nutritional counseling in reducing mobility disability in older adults with physical frailty and sarcopenia^13^. Our study builds on these findings by integrating additional therapeutic modalities, such as involving pharmacists in managing polypharmacy adherence and acupoint massage, further enhancing functional outcomes. In addition to frailty and physical function metrics, our study also evaluated the impact of multidisciplinary intervention on clinical events, including hospital readmissions, IADL and anxiety, reflecting a more holistic outcome assessment aligned with real-world patient priorities. This integration offers a new intervention mode to frailty management that has not been extensively explored in previous studies.

While Kitzman et al. reported short-term improvements in physical function following tailored rehabilitation in older patients with heart failure, 97% of whom were frail or prefrail, our study demonstrated sustained benefits over 12 months, including improvements in frailty status and physical function, and was also associated with fewer all-cause readmissions^7^. This extended follow-up period provides evidence of the sustained 12-month benefit of applied multidisciplinary approach (RENAP) in elderly frail CVD patients, enriched the current literature for approach which might be useful to improve outcome of patients with similar disease features tested in this clinical trial.

Our research suggests that the multidisciplinary RENAP approach may contribute to meaningful improvements in frailty, as measured by FFP score. Notably, the CFS score, a measure derived from the deficit-accumulation approach to frailty, was also significantly improved. It is worth noting that the FFP and CFS represent two conceptually distinct approaches to defining frailty: the former is based on a specific phenotype, while the latter relies on the accumulation of deficits across multiple domains. Despite these conceptual differences, both showed convergent improvement following the RENAP intervention. To our knowledge, limited intervention studies have reported simultaneous benefits across both of these frailty measures. This convergence across two complementary frailty frameworks strengthens the evidence that the RENAP intervention produces meaningful, multidomain enhancements in muscle strength, physical endurance and overall functionality.

The improvements observed are particularly relevant given that frailty in CVD patients is driven by complex biological processes, including inflammation and sarcopenia^14^. Comprehensive interventions that address these multifactorial issues can synergistically enhance physiological reserves and overall health^15^. A 6-month integrated care programme incorporating exercise, nutrition and psychological support has demonstrated significant health benefits, including measurable reductions in frailty^16^. Our findings not only align with existing evidence but also extend it by establishing a clear benefit for elderly patients with CVD.

Our study also found that the RENAP intervention was associated with a lower risk of all-cause readmission among frail elderly patients with CVD. This finding aligns with research by Kamiya et al., which showed that multidisciplinary outpatient cardiac rehabilitation improves long-term survival and reduces rehospitalisation rates in diverse heart failure patients^17^. Similarly, Reeves et al. found that a multidomain physical rehabilitation programme, initiated in-hospital and continued post-discharge, effectively reduces rehospitalisations in frail elderly patients with acute decompensated heart failure^18^. Hospital environments often exacerbate frailty in CVD patients through immobilization, fasting, sleep deprivation and disorientation, leading to muscle mass loss and “posthospital syndrome”^19^. These conditions contribute to higher rehospitalisation rates, increased mortality, and elevated healthcare costs, highlighting the need for interventions that delay or reverse frailty^3^. Although mortality was lower in the intervention group, the difference did not reach statistical significance, likely due to the small number of events (*n* = 15) and consequent low statistical power. Notably, the point estimate for mortality (HR = 0.53) was identical to that for hospital readmissions, which did show a statistically significant benefit. However, the wide confidence interval (0.16–1.70) is consistent with both a clinically meaningful protective effect and no effect. Furthermore, given the limited number of mortality events, analyses of mortality-related covariates should be regarded as exploratory and interpreted cautiously. Therefore, no firm conclusion can be drawn regarding mortality from this study, and future adequately powered trials using a larger sample size are needed to address this question.

Regarding safety, the temporal pattern of falls deserves comment. At each individual time point, there were no statistically significant differences in fall incidence between the two groups. However, an exploratory inspection of the data revealed that the intervention group experienced more new falls during the first six months but fewer during the 6–12 month period. This pattern raises the possibility that the RENAP intervention might be associated with an initial increase in fall risk, potentially due to increased mobility, followed by a longer-term trend toward fewer falls, which could reflect improved balance and physical function. Nevertheless, given the small numbers and the lack of statistical significance at individual time points, these observations are hypothesis-generating only and should be interpreted with caution.

We also observed improvements in HADS-A scores, suggesting a potential benefit of the RENAP intervention on anxiety symptoms and psychological well-being in frail elderly patients with CVD. Given the increased susceptibility to anxiety and depression in this demographic^20^, early identification and targeted interventions are crucial for managing frailty effectively. Research strongly supports the connection between emotional well-being and physical health in these patients^21^. A meta-analysis revealed a reciprocal relationship between depression and frailty in older adults^22^. Regular exercise improves mood and the RENAP intervention that include exercise helps alleviate anxiety with additional benefits. Similarly, Fountotos et al. found that an in-hospital multicomponent intervention targeting frailty in older patients with acute CVD led to meaningful improvements in quality of life and mental well-being^23^.

Our study also demonstrated improvements in IADL scores, suggesting that the RENAP intervention may help maintain or enhance the ability to perform daily activities in frail elderly patients with CVD. These findings align with previous research highlighting the benefits of multi-component interventions^24^.

Taken together, our study corroborates existing evidence while introducing a comprehensive approach to managing frailty in elderly patients with CVD, offering clinically meaningful insights with evidence of long-term benefits, which might help decision-making in future clinical practices for managing frailty in elderly CVD patients.

Our longitudinal analysis revealed a time-dependent treatment effect, which offers crucial insight into the intervention’s mechanism of action. The parallel, modest improvements in frailty (FFP) and comparable changes in physical function (SPPB) observed in both groups during the first 6 months suggest that the natural recovery process following hospital discharge was the dominant influence, potentially obscuring the specific effect of the RENAP programme in the short term. The critical divergence emerged between 6 and 12 months. The control group experienced a rebound in frailty and stagnation in physical function, a pattern indicative of the natural, unsupported course for these frail patients. In stark contrast, the RENAP group not only maintained its frailty improvements but also achieved significant functional recovery. This pattern underscores that the principal value of our sustained, multi-component intervention lies not in accelerating immediate post-discharge recovery, but in its potential to sustain improvements in frailty and physical function over 12 months and to slow the progression of frailty in this high-risk population.

The strong correlation between frailty and cardiovascular outcomes underscores the need for comprehensive frailty metrics as predictors of cardiovascular health^5,25^. As the global population ages, it is imperative to develop and implement strategies that improve both physical and mental health outcomes for elderly CVD patients^26^. Our RENAP approach, which integrates various facets of care, offers a model that can be adapted to different settings worldwide, paving the way for innovative strategies in managing elderly frail CVD patients. This strategy aligns with the growing emphasis on personalised medicine and patient-centred care, which are increasingly recognized as essential for managing chronic conditions in aging populations^27^.

Integrating these findings into clinical practice could support further evaluation of integrated care pathways toward more inclusive and coordinated care strategies that address the complex needs of elderly patients. Such strategies would aim to reduce hospital admissions, marking a significant advancement in geriatric CVD care. This shift from traditional care models to holistic, patient-centred strategies has the potential to improve patient-centred outcomes if confirmed in broader settings.

Effectively managing frail elderly CVD patients requires comprehensive education that extends beyond disease-specific information. These programmes should include essential self-management topics such as frailty awareness, fall prevention, adherence to treatment and comprehensive intervention, dietary guidance and lifestyle changes. Integrating frailty education into standard care has been linked to better outcomes, though challenges like varying literacy levels and cultural differences may affect effectiveness.

Patient adherence is very important for such comprehensive assessments and interventions. Increasing the frequency of contact with patients can enhance adherence. Utilizing appropriate educational materials and technology during follow-ups can also help overcome these barriers and enhance the programme’s impact. In this study, 120 out of 166 participants (72.3%) randomised to the intervention group met the pre-specified adherence criteria. Several strategies were adopted to improve patient adherence, as detailed in the study protocol. These included patient education, the use of a personalised patient handbook for self-monitoring, regular brief telephone support to provide guidance and encouragement, and the tailoring of exercise and dietary plans to individual tolerance and preferences. The fact that a complex, long-term intervention could be sustained in a population of frail, elderly patients with CVD suggests that the programme was not only effective but also feasible and acceptable to the target population, underscoring the potential for integrating such a supported model into routine care pathways.

Successful multidisciplinary interventions rely on forming and training a diverse team of specialists, including cardiologists, nurses, rehabilitation therapists, nutritionists, TCM practitioners and pharmacists. Establishing standardised operating procedures (SOPs) and ongoing training ensures the team remains aligned and up-to-date with healthcare advancements, which is crucial for meeting patient needs. As highlighted by Richter et al., a well-coordinated team enhances care through personalised strategies^28^. Lettino et al. also emphasize the importance of inclusive clinical trials and real-world studies to optimize treatment and improve quality of life^29^. Focusing on patient-centred care within a structured, multidisciplinary framework helps healthcare providers meet global standards of integrated care.

Our study has several limitations. First, it was conducted in three tertiary hospitals in Beijing, which might limit the generalizability to other regions or healthcare settings with different demographics and resources. Second, the one-year follow-up period may not fully capture the long-term effects of the RENAP intervention on frailty and related outcomes. Third, participants’ adherence and missing data have a significant impact on the research outcomes. The use of a 50% adherence rate, which was relatively low, may introduce bias^30^. Higher adherence thresholds are needed in future studies to clarify the dose-response relationship. Adherence to the intervention was self-reported, potentially introducing reporting bias; objective measures like wearable trackers or dietary logs could provide more accurate data. The differential contact frequency between groups may represent another potential confounding factor, as the additional communication in the intervention group could have contributed to the observed effects beyond the specific intervention components. Participants lost to follow-up differed from completers in some baseline characteristics; therefore, residual attrition bias cannot be excluded despite ITT imputation and PPS sensitivity analyses. Fourth, due to insufficient data, a formal cost-effectiveness analysis was not undertaken, even though the medical insurance expenses showed no significant difference between the intervention and control groups (Supplementary Table 5). Future research should evaluate the economic impact and feasibility of implementing these programmes in broader clinical practice. Fifth, 5-level European Quality of Life 5 Dimensions index (EQ-5D) was not systematically assessed due to feasibility constraints in the baseline comprehensive geriatric assessments (CGA). Its omission at baseline was a pragmatic decision to reduce assessment burden. Critically, multidomain alternatives (SPPB for mobility, IADL for self-care, HADS-A for psychological state) comprehensively captured health status and demonstrated intervention efficacy. Subgroup analyses were pre-specified and intended to assess the consistency of the intervention effect across clinically relevant subgroups. Formal interaction tests were not performed, as these analyses were exploratory in nature and the study was not powered for between-subgroup comparisons. Therefore, the subgroup findings should be interpreted as descriptive and hypothesis-generating rather than confirmatory. Lastly, the study design did not isolate the specific contributions of each intervention component. Furthermore, while interventions like acupoint massage were included to address insomnia and constipation, the lack of systematic, quantitative assessment of these specific outcomes limits our ability to draw definitive conclusions about their efficacy. Evaluating the efficacy of individual components and their potential synergies represents an important goal for future research and future studies using factorial designs could help identify the most effective components of multidisciplinary interventions.

In conclusion, in this multicentre, single-blinded randomised controlled trial, a hospital-initiated multidisciplinary RENAP programme improved frailty status and physical function at 12 months in frail elderly inpatients with CVD. The intervention was non-invasive and appeared feasible within routine inpatient care. RENAP was also associated with fewer all-cause readmissions, although this secondary clinical outcome requires confirmation in future studies. Further studies in larger and more diverse healthcare settings are needed to confirm generalisability, clarify which intervention components contribute most to benefit, and evaluate longer-term clinical outcomes.

## Methods

### Design

This multicentre, randomised controlled trial was conducted from June 2019 to June 2022 across three tertiary hospitals in Beijing, China^11^. The study protocol was pre-registered with the China Clinical Trials Registry (ChiCTR1900022623) on April 19, 2019, prior to the first patient enrollment, and patient enrollment is complete. The full protocol is available at https://www.chictr.org.cn/showproj.html?proj=38141. The study protocol was approved by the Ethics Committee of Beijing Hospital (2019BJYYEC-045-02), the Ethics Committee of the General Hospital of the People’s Liberation Army (S2019-208-01), and the Ethics Committee of Beijing Tsinghua Changgung Hospital (18166-0-01). The trial adhered to the Declaration of Helsinki and all relevant regulations, with written informed consent obtained from all participants. The authors affirm adherence to the trial protocol during its implementation and conduct, ensuring accuracy and completeness. The corresponding author (Hua Wang) affirms that this manuscript is an honest, accurate, and transparent account of the study being reported; that no important aspects of the study have been omitted; and that any discrepancies from the study as originally planned have been explained. The trial was monitored by an independent Data and Safety Monitoring Board. Participants did not receive financial compensation for their participation. The Peking University Clinical Research Institute established the electronic data capture (EDC) system (Research Electronic Data Capture version 10.6.22) for this trial and was responsible for data analysis and quality control throughout the trial. Data were collected using this system, recorded in real time, and stored on secure servers with access restricted to authorised study personnel.

### Participants

Potential participants were systematically identified by the research team through daily screening of all newly admitted elderly patients from the cardiac wards of Beijing Hospital, the General Hospital of the People’s Liberation Army, and Beijing Tsinghua Changgung Hospital. The inclusion criteria were: (1) age ≥ 70 years; (2) FFP score ≥3; and (3) long-term residency in Beijing. The exclusion criteria were: (1) inability to complete rehabilitation exercises or the SPPB assessment; (2) cognitive impairment or severe comorbidities including but not limited to metastatic cancer, end-stage renal disease on dialysis, severe chronic liver disease (e.g., Child-Pugh class C cirrhosis), or any other condition with a clinical life expectancy of less than 12 months as judged by the attending physician; (3) recent engagement in regular physical exercise at moderate intensity or treatment in nutrition, rehabilitation, or TCM departments within the last three months; (4) long-term residence in a hospital or nursing home; and (5) participation in other intervention trials. For patients who preliminarily met the criteria, written informed consent was obtained before any study procedures were initiated. The first patient was enrolled on 3 June 2019, and the last patient was enrolled on 10 June 2021. A formal baseline assessment was conducted, including the FFP evaluation to confirm they met all inclusion criteria before being randomised into the trial.

### Randomisation and blinding

After obtaining consent and completing baseline assessments, participants were randomly assigned to either the intervention or control group in a 1:1 ratio using a central computer system within the EDC system. Simple randomisation without stratification or blocking was used. The randomisation sequence was generated to ensure no manual interference. The allocation sequence was statistically unpredictable, and group assignment was only revealed after participant enrolment, informed consent and baseline assessments were completed. Blinding was maintained for endpoint evaluators and data analysts; however, due to the nature of the interventions, participants and multidisciplinary treatment teams were not blinded. The results were not stratified by hospital in the analysis. Individual hospitals did not have the authority to terminate the study independently; termination of the trial required approval by the principal investigator.

### Interventions

**Fig. 4:**
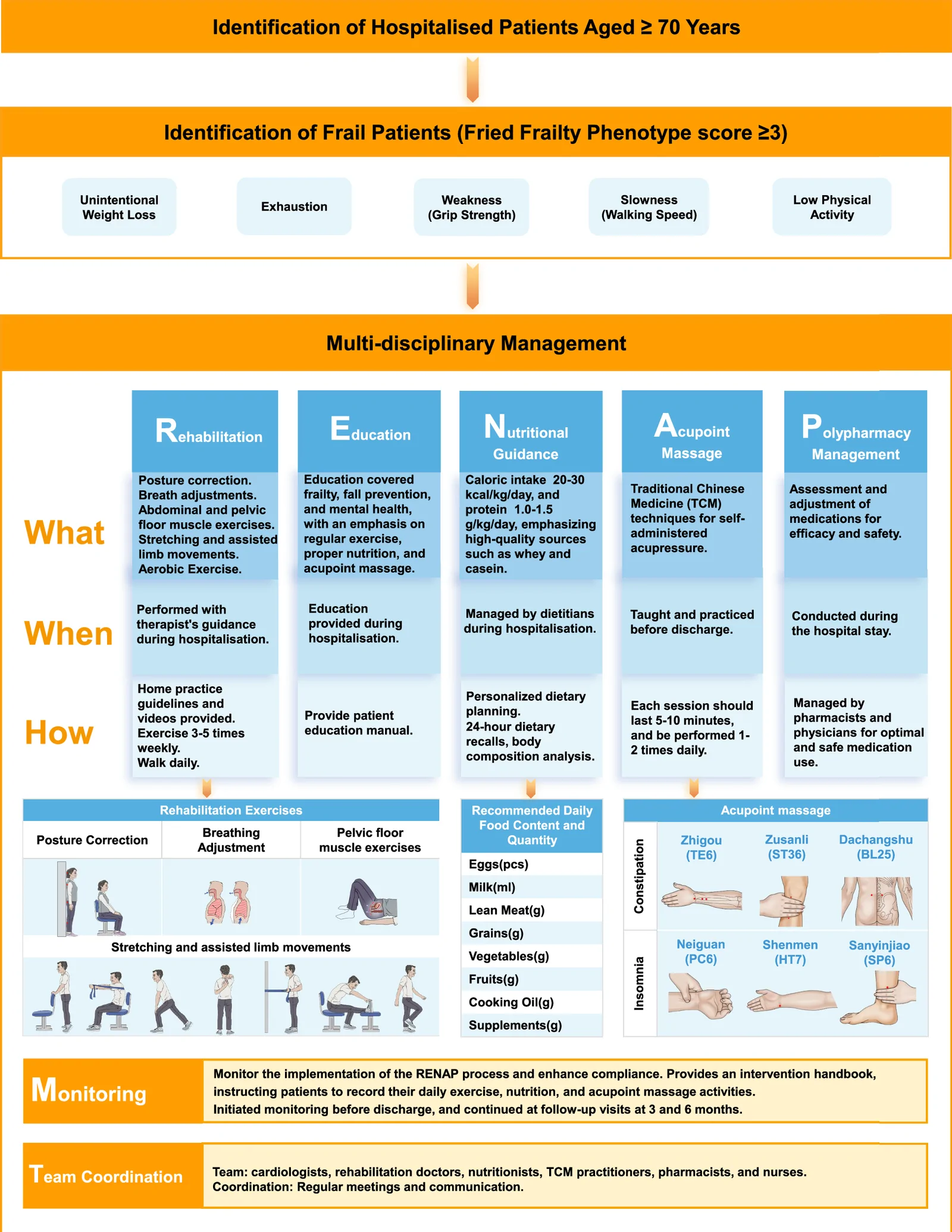
Multidisciplinary intervention protocol used during the trial. This figure illustrates the multidisciplinary intervention protocol used in the study. Participants identified as frail (Fried Frailty Phenotype score ≥3) were enrolled in the year-long RENAP programme, focusing on improving overall health and quality of life through personalised interventions. The protocol included rehabilitation exercises, frailty education, nutritional guidance, acupoint massage, and polypharmacy management, all tailored to meet individual patient needs. Symbols like arrows indicate the sequence of interventions. RENAP Rehabilitation, Education, Nutritional Guidance, Acupoint Massage, Polypharmacy Management; TCM traditional Chinese medicine.

The intervention cohort participated in a year-long, personalised multidisciplinary programme (RENAP) based on the problems identified by CGA. CGA included functional capacity assessment, nutritional status assessment, medication review, insomnia assessment, and TCM constitution and symptom assessment. Reproducibility was ensured through detailed flowcharts (Fig. 4) and SOPs (Appendix 1). The RENAP intervention consisted of the following components:

1. Rehabilitation Exercises: This component involved breath and posture adjustments, abdominal and pelvic floor exercises, stretching and assisted limb movements. Rehabilitation physicians supervised these activities during hospitalisation and provided video instructions for home practice. Participants performed 3 to 20 repetitions for each exercise and 3 to 5 times weekly for 12 months. The frequency, time and feelings concerning exercise each week were recorded. The exercise intensity was progressively adjusted by the supervising rehabilitation physicians. The progression was guided by the patient’s subjective tolerance and clinical observation for any adverse effects. The intensity remained at a “safe level,” which was defined as the highest level not associated with pain, adverse symptoms or functional deterioration. Daily walking was also encouraged.
2. Patient Education: A manual was distributed to participants, covering essential topics such as frailty, fall prevention, and mental health. Emphasis was placed on the importance of regular exercise, proper nutrition, and acupoint massage.
3. Nutritional Guidance: Dietitians conducted 24-h dietary recalls and analysed body composition and laboratory markers to develop individualised nutritional plans. Caloric intake recommendations ranged from 20 to 30 kcal/kg/day, and the target protein intake was at 1.0–1.5 g/kg/day, with more than 50% being high-quality sources such as whey and casein. Adjustments were made for specific conditions, such as chronic kidney disease. Carbohydrates were recommended to constitute 50–65% of total energy intake, fats were limited to 35%, with saturated fats under 10%, protein intake corresponded to approximately 15–20% of the total energy intake, and dietary fiber intake was set at 25–30 g/day.
4. Acupoint Massage: TCM physicians assessed the participants based on the TCM theory of syndrome differentiation, including a TCM tongue diagnosis and pulse diagnosis^31^. Tongue diagnosis provides visual information about the patient’s health status by examining the tongue’s color, shape and texture^32^. Pulse diagnosis assesses the quality, rhythm, and strength of the pulse to evaluate the functional status of internal organs^33^. The examinations, combined with other clinical symptoms and signs, was integrated to determine the TCM syndrome of each participant. Then, TCM practitioners guided participants in self-administered acupoint massage. For constipation, points including Zhigou, Zusanli and Dachangshu were selected to regulate intestinal function. For insomnia, points included Neiguan, Shenmen and Sanyinjiao were selected in calming the mind and improving sleep quality. A detailed description of acupoint localization and massage procedure is provided in the Supplementary Materials (Appendix 1 and 2). Following guidance from TCM practitioners, participants were instructed to perform self-administered acupoint massage at home. Each self-administered session lasted 5–10 min and was performed 1–2 times daily.
5. Polypharmacy Guidance: Clinical pharmacists assessed the appropriateness, efficacy and safety of participants’ medications. They then collaborated with the patient’s attending physician to develop a consensus-based treatment plan. This plan was discussed with the participant in a shared decision-making process before implementation. Medication use was re-evaluated at follow-ups, and this collaborative process was repeated to adjust treatments as needed. Detailed procedures are provided in the Supplementary Materials.
6. Multidisciplinary Team: The intervention was implemented by an interdisciplinary team of professionals from cardiology, rehabilitation, nursing, nutrition, TCM and pharmacy, responsible for designing and executing the intervention components.
7. Training and Coordination: Team members received specialized training on the standardised comprehensive multidisciplinary intervention protocols to ensure a unified approach to patient management and effective collaboration. Regular meetings facilitated ongoing communication, allowing for timely updates on patient progress and necessary adjustments to treatment plans.
8. Follow-Up and Supervision: Participants were provided with a manual to record daily exercise, meal intake, and acupoint massage activities (Appendix 2). Monthly follow-ups via telephone and WeChat were conducted to monitor adherence to dietary and exercise routines, as well as acupressure practices. Interventions were adjusted during the 3- and 6-month follow-ups. Participants in the intervention group were defined as treatment-compliant if they completed at least 50% of the planned rehabilitation exercises and/or 50% of their planned nutrition guidance programme and 50% of the acupoint massage prescribed by TCM within 12 months.

Participants in the control group received standard medical management, routine monitoring, and general advice on diet and lifestyle. After discharge, they received the standard follow-up provided by the hospital. No structured, multidisciplinary intervention targeting frailty was provided. All participants, including the control group, underwent the same schedule of research assessments (e.g., FFP, SPPB) at baseline, three, six and twelve months.

### Outcomes

The primary outcomes were changes from baseline to 12 months in FFP and SPPB scores. The FFP score, providing a holistic, syndromic classification of an individual’s overall vulnerability, assesses frailty through criteria: unintentional weight loss >5% of body weight in the past year, self-reported exhaustion, weakness (low grip strength), slow walking speed, and low physical activity. The FFP score was calculated as the sum of the five criteria, yielding a total score ranging from 0 to 5. For primary analysis, the FFP score was treated as a continuous variable. Frailty was defined as a score of ≥3, consistent with the original definition^2^. The SPPB score, as an objective physical performance measure, assesses standing balance, walking speed, and the ability to rise from a chair, with scores ranging from 0 to 12 and higher scores indicating better mobility. The SPPB was administered according to the standardised protocol developed by the National Institute on Aging. Secondary outcomes included CFS^34^, basic activities of daily living (BADL, assessing disability), EQ-5D (assessing health-related quality of life), medical insurance expenses, fall events, nutrition risk screening 2002, all-cause mortality, and non-elective hospital readmission at one year, which was defined as any unplanned admission via the Emergency Department or following a clinical visit. Non-pre-specified exploratory outcomes included IADL (assessing the ability to independently perform daily activities), HADS-A (assessing anxiety), and Pittsburgh Sleep Quality Index score (assessing sleep quality) at one year.

### Statistical analysis

The sample size was calculated based on a two-sided, two-sample *t*-test (power = 80%, *α* = 0.05). Based on refs. ^35,36^, we assumed a between-group mean difference of 0.39 points in the FFP score reduction, with a common standard deviation of 1.10, requiring 250 participants (125 per group). For the SPPB score, we assumed a between-group mean difference of 1.40 points, with a common standard deviation of 2.40^35^, requiring 94 participants (47 per group). The larger of these estimates (250 participants) was used. Considering an attrition rate of 20%, a minimum of 320 participants was recruited.

The study included two analysis sets: the ITT analysis set and the PPS set. The ITT set included all eligible participants who were randomised. The PPS was a subset of the ITT, comprising participants who completed the study per protocol. For the intervention group, this required completing at least 50% of the prescribed RENAP components (rehabilitation exercises, nutritional guidance and acupoint massage) and having no missing primary outcome data. For the control group, this required receiving standard medical management without crossover to the RENAP intervention, attending all scheduled follow-up assessments, and having no missing primary outcome data. Demographic, baseline analyses were conducted using the ITT, while efficacy analysis was primarily based on the ITT. Sensitivity analyses of efficacy outcomes were performed using the PPS.

Statisticians developed a statistical analysis plan in collaboration with the principal investigator after the protocol was finalized (Appendix 3 and 4). Statistical analysis was performed using SAS 9.4 (Simplified Chinese edition; SAS Institute, Cary, NC). All statistical tests were two-tailed, with *P* < 0.05 considered statistically significant. Baseline characteristics were presented descriptively without formal statistical testing, in accordance with CONSORT guidelines. For other comparisons, continuous variables were compared using paired *t*-tests or Wilcoxon rank-sum tests. Categorical variables were compared using chi-square tests or Fisher’s exact tests.

Primary efficacy analysis-The FFP score, SPPB score and changes from baseline to 12 months in these scores were analysed as repeated measures (longitudinal) data, using both analysis of variance (ANOVA) and mixed-effects models. Repeated measures ANOVA was used to compare between-group differences at each visit. Covariance analysis was applied to evaluate the FFP and SPPB scores, adjusting for study central effects, with adjusted means and 95% CIs calculated for differences between the two groups. A mixed-effects model was applied to estimate least squares means (Lsmeans) and corresponding 95% CIs for FFP score and SPPB score changes, with the individual considered as a random effect. The handling of missing data was pre-specified. In the ITT analysis, missing values for the primary outcomes were imputed using the expectation-maximization method, with one imputation performed and a fixed seed to ensure reproducibility. In the PPS analysis, no imputation was performed for missing data.

Secondary efficacy analysis-For longitudinal endpoints, statistical summaries and between-group comparisons were conducted using ANOVA and mixed-effects models. For time-to-event endpoints, survival functions for all-cause mortality and hospital readmission were estimated using the Kaplan–Meier method, with log-rank tests comparing the two groups. Multivariable Cox proportional hazards regression models were used to evaluate HR between groups for these events. The proportional hazards assumption was assessed using Schoenfeld residual-based methods and was satisfied for the treatment variable.

Subgroup analyses-Subgroup analyses were conducted to assess whether the beneficial effect of the RENAP intervention was consistently observed across clinically relevant subgroups. The pre-specified subgroups were chosen based on a priori clinical relevance rather than post-hoc data exploration, including sex, age (≥ 80, < 80 years), body mass index (BMI, >24 kg/m^2^, 18 ≤ BMI ≤ 24), heart failure, coronary artery disease, atrial fibrillation/flutter and diabetes. Between-group differences in primary outcomes (RENAP vs. usual care) were assessed using two-sample *t*-tests for normally distributed data or Wilcoxon rank-sum test for non-normally distributed data. Subgroup analyses of all-cause readmission were performed using Cox proportional hazards models within each subgroup. Results were presented as forest plots showing between-group differences with 95% CIs for each subgroup.

### Reporting summary

Further information on research design is available in the Nature Portfolio Reporting Summary linked to this article.

## Supplementary information

Supplementary Information

Reporting Summary

Transparent Peer Review file

## Source data

Source Data

## Data Availability

The raw participant-level clinical data from this randomised controlled trial cannot be publicly deposited because the institutional review board approval (Beijing Hospital Ethics Committee, 2019BJYYEC-045-02) explicitly restricts data access to the study team to protect participant privacy, and the informed consent obtained from participants did not include provisions for open data sharing. Additionally, national health data regulations in China prohibit the unrestricted public release of individual-level clinical data. Aggregate and summary-level numerical source data underlying the figures and tables in the main manuscript and Supplementary Information are provided as a Source Data file with this paper. For analyses involving missing outcome data, the Source Data file reports aggregate model estimates from the ITT analyses; individual observed or imputed participant-level data are not publicly provided. Additional de-identified participant-level data not included in the Source Data file may be made available to qualified academic investigators for non-commercial scientific research. Data access requests should be submitted to the corresponding author (Hua Wang, email: wh74220@aliyun.com) and must include: (1) a brief research proposal stating the purpose and planned analyses; (2) a data confidentiality commitment form; and (3) a completed data use agreement specifying the intended use and duration of access. All requests will be reviewed by the study investigators and the institutional ethics committee. Approved requests will receive a response within 4–6 weeks, and data will be provided via a secure file transfer mechanism. Data will be available for a period of up to 2 years for the specified project only. Source data are provided with this paper.

## Code availability

The custom SAS programs used for data processing and statistical analyses in this study have been deposited in Zenodo (https://doi.org/10.5281/zenodo.21026032)^37^. The repository contains all analysis code used for baseline analyses, longitudinal mixed-effects models, survival analyses, subgroup analyses, and safety analyses. The code was developed using SAS 9.4 (Simplified Chinese edition). Users should update the local file paths before running the programs.

## References

[1] Fane, M. & Weeraratna, A. T. How the ageing microenvironment influences tumour progression. *Nat. Rev. Cancer* **20**, 89–106, 10.1038/s41568-019-0222-9 (2020). PMC7377404 31836838

[2] Fried, L. P. et al. Frailty in older adults: evidence for a phenotype. *J. Gerontol. A Biol. Sci. Med. Sci* **56**, M146–M156, 10.1093/gerona/56.3.m146 (2001). 11253156

[3] Hoogendijk, E. O. et al. Frailty: implications for clinical practice and public health. *Lancet* **394**, 1365–1375, 10.1016/S0140-6736(19)31786-6 (2019). 31609228

[4] Dent, E. et al. Management of frailty: opportunities, challenges, and future directions. *Lancet* **394**, 1376–1386, 10.1016/S0140-6736(19)31785-4 (2019). 31609229

[5] Shrauner, W. et al. Frailty and cardiovascular mortality in more than 3 million US Veterans. *Eur. Heart J.* **43**, 818–826, 10.1093/eurheartj/ehab850 (2022). PMC9890630 34907422

[6] Ijaz, N. et al. Interventions for frailty among older adults with cardiovascular disease: JACC state-of-the-art review. *J. Am. Coll. Cardiol.* **79**, 482–503, 10.1016/j.jacc.2021.11.029 (2022). PMC8852369 35115105

[7] Kitzman, D. W. et al. Physical rehabilitation for older patients hospitalized for heart failure. *N. Engl. J. Med.* **385**, 203–216, 10.1056/NEJMoa2026141 (2021). PMC8353658 33999544

[8] Quach, J. et al. Admission frailty and its changes during cardiac rehabilitation: associations with 5-year outcomes. *Eur. J. Prev. Cardiol.* **30**, 807–819, 10.1093/eurjpc/zwad048 (2023). PMC10335868 36799963

[9] Waits, A., Tang, Y. R., Cheng, H. M., Tai, C. J. & Chien, L. Y. Acupressure effect on sleep quality: a systematic review and meta-analysis. *Sleep Med. Rev.* **37**, 24–34, 10.1016/j.smrv.2016.12.004 (2018). 28089414

[10] Aydemir, T. et al. The effect of acupressure on constipation symptoms and quality of life in older people: a mixed-methods study. *Eur. Geriatr. Med.* **14**, 1135–1144, 10.1007/s41999-023-00842-6 (2023). 37477804

[11] Yao, S. et al. The effect of comprehensive assessment and multi-disciplinary management for the geriatric and frail patient: a multi-center, randomized, parallel controlled trial. *Medicine* **99**, e22873, 10.1097/MD.0000000000022873 (2020). PMC7668452 33181655

[12] Fairhall, N. et al. Effects of a multifactorial interdisciplinary intervention on fall-related risk factors and fall rate in frail older adults. *Age Ageing* **43**, 616–622, 10.1093/ageing/aft204 (2014). 24381025

[13] Bernabei, R. et al. Multicomponent intervention to prevent mobility disability in frail older adults: results from the SPRINTT project. *BMJ* **377**, e068788, 10.1136/bmj-2021-068788 (2022). PMC9092831 35545258

[14] Sato, R., Vatic, M., Peixoto, D. F. G., Anker, S. D. & von Haehling, S. Biological basis and treatment of frailty and sarcopenia. *Cardiovasc. Res.* **120**, 982–998, 10.1093/cvr/cvae073 (2024). 38828887

[15] Taylor, J. A. et al. Multisystem physiological perspective of human frailty and its modulation by physical activity. *Physiol. Rev.* **103**, 1137–1191, 10.1152/physrev.00037.2021 (2023). PMC9886361 36239451

[16] Chan, D. D. et al. Integrated care for geriatric frailty and sarcopenia: a randomized control trial. *J. Cachexia Sarcopenia Muscle* **8**, 78–88, 10.1002/jcsm.12132 (2017). PMC5326822 27897406

[17] Kamiya, K. et al. Multidisciplinary cardiac rehabilitation and long-term prognosis in patients with heart failure. *Circ. Heart Fail.* **13**, e006798. 10.1161/CIRCHEARTFAILURE.119.006798 (2020). 32986957

[18] Reeves, G. R. et al. A novel rehabilitation intervention for older patients with acute decompensated heart failure: The REHAB-HF Pilot Study. *JACC Heart Fail.* **5**, 359–366, 10.1016/j.jchf.2016.12.019 (2017). PMC5409854 28285121

[19] Welch, C., Chen, Y., Hartley, P. & Gordon, A. L. New horizons in hospital-associated deconditioning: a global condition of body and mind. *Age Ageing* **53**, afae241. 10.1093/ageing/afae241 (2024). PMC11534583 39497271

[20] Dapp, U., Minder, C. E., Golgert, S. & von Renteln-Kruse, W. The inter-relationship between depressed mood, functional decline and disability over a 10-year observational period within the Longitudinal Urban Cohort Ageing Study (LUCAS). *J. Epidemiol. Community Health* **75**, 450–457, 10.1136/jech-2020-214168 (2021). PMC8053334 33158941

[21] Davoodian, N. et al. Contribution of depression and cardiometabolic diseases and the role of depression treatment in survival and functioning in older adults. *eClinicalMedicine* **82**, 103182. 10.1016/j.eclinm.2025.103182 (2025). PMC11999077 40235951

[22] Soysal, P. et al. Relationship between depression and frailty in older adults: a systematic review and meta-analysis. *Ageing Res. Rev.* **36**, 78–87, 10.1016/j.arr.2017.03.005 (2017). 28366616

[23] Fountotos, R. et al. Multicomponent intervention for frail and pre-frail older adults with acute cardiovascular conditions: outcomes from the TARGET-EFT randomized clinical trial. *J. Am. Geriatr. Soc.* **71**, 1406–1415, 10.1111/jgs.18228 (2023). 36645227

[24] Tarazona-Santabalbina, F. J. et al. A multicomponent exercise intervention for reversing frailty and improving cognition, emotion, and social networking in the frail elderly: a randomized clinical trial. *J. Am. Med. Dir. Assoc.* **17**, 426–433, 10.1016/j.jamda.2016.01.019 (2016). 26947059

[25] He, D. et al. Changes in frailty status and incident cardiovascular disease in three prospective cohorts. *Eur. Heart J.* **45**, 1058–1068, 10.1093/eurheartj/ehae175 (2024). 38241094

[26] Leong, D. P. et al. Frailty and its impact on outcomes in heart failure patients from high-, middle-, and low-income countries. *Eur. Heart J.* **44**, 4435–4444, 10.1093/eurheartj/ehad595 (2023). PMC10635666 37639487

[27] Forman, D. E. et al. The role of geroscience in shaping therapeutic strategies for older adults with cardiovascular disease: JACC scientific statement. *J. Am. Coll. Cardiol.* **82**, 631–647, 10.1016/j.jacc.2023.05.038 (2023). PMC10414756 37389519

[28] Richter, D. et al. Defining frailty in cardiology: assessment tools and clinical implications. A consensus document of the European Society of Cardiology. *Eur. J. Prev. Cardiol.* **29**, 216–227, 10.1093/eurjpc/zwaa167 (2022). 34270717

[29] Lettino, M. et al. Cardiovascular disease in the elderly: proceedings of the European Society of Cardiology-Cardiovascular Round Table. *Eur. J. Prev. Cardiol.* **29**, 1412–1424, 10.1093/eurjpc/zwac033 (2022). 35167666

[30] Páez, A., Nunan, D., McCulloch, P. & Beard, D. The influence of intervention fidelity on treatment effect estimates in clinical trials of complex interventions: a meta-epidemiological study. *J. Clin. Epidemiol.* **177**, 111610. 10.1016/j.jclinepi.2024.111610 (2025). 39528004

[31] Jiang, M. et al. Syndrome differentiation in modern research of traditional Chinese medicine. *J. Ethnopharmacol.* **140**, 634–642, 10.1016/j.jep.2012.01.033 (2012). 22322251

[32] Xie, J. et al. Digital tongue image analyses for health assessment. *Med. Rev.* **1**, 172–198, 10.1515/mr-2021-0018 (2022). PMC10388765 37724302

[33] Matos, L. C., Machado, J. P., Monteiro, F. J. & Greten, H. J. Can Traditional Chinese Medicine diagnosis be parameterized and standardised? A narrative review. *Healthcare* **9**, 177, 10.3390/healthcare9020177 (2021). PMC7914658 33562368

[34] Rockwood, K. & Theou, O. Using the clinical frailty scale in allocating scarce health care resources. *Can Geriatr J* **23**, 210–215, 10.5770/cgj.23.463 (2020). PMC7458601 32904824

[35] Cameron, I. D. et al. A multifactorial interdisciplinary intervention reduces frailty in older people: randomized trial. *BMC Med.* **11**, 65, 10.1186/1741-7015-11-65 (2013). PMC3751685 23497404

[36] Ng, T. P. et al. Nutritional, physical, cognitive, and combination interventions for frailty reversal in older adults: a randomized controlled trial. *Am. J. Med.* **128**, 1225–1236, 10.1016/j.amjmed.2015.06.017 (2015). 26159634

[37] Chai, K. et al. SAS code for ‘Multidisciplinary intervention in frail elderly patients with cardiovascular diseases: a multicentre, single-blinded, randomised controlled trial. *Zenodo*10.5281/zenodo.21026032 (2026).42811040

