# Supplementary material for: Multidisciplinary intervention in frail elderly patients with cardiovascular diseases: a multicentre, single-blinded, randomised controlled trial
Source: Nat Commun. 2026 Aug 31;17:10340. doi: 10.1038/s41467-026-77277-7 (PMC13623951; doi:10.1038/s41467-026-77277-7)
Supplement: Supplementary file 1 [file 41467_2026_77277_MOESM1_ESM.pdf]

Multidisciplinary Intervention in Frail Elderly Patients With Cardiovascular Diseases: a multicentre, single-blinded, randomised controlled trial

Supplementary Information

Supplementary Tables ……….……. page 3

- Supplementary Table 1: *Baseline Characteristics of Patients in the PPS* *(page 3)*
- Supplementary Table 2: *Comparison of Characteristics Between Participants Who Completed Follow-up and Those Lost to Follow-up* *(page 5)*
- Supplementary Table 3: *Comparison of Characteristics Among Participants Lost to Follow-up in the Intervention and Control Groups* *(page 6)*
- Supplementary Table 4: *Estimated Between-group Adjusted Mean Differences in Change From Baseline to 12 Months in the PPS* *(page 7)*
- Supplementary Table 5: *Between-Group Differences in secondary outcomes at 1-Year Follow-Up* *(page 8)*
- Supplementary Table 6: *Cox proportional hazards regression model analysis for all-cause readmission and mortality* *(page 9)*
- Supplementary Table 7: *New Falls Occurring during each Follow-up period* *(page 10)*

Supplementary Figures ………….. page 11

- Supplementary Figure 1: *Impact of Multidisciplinary Intervention on Frailty, Physical Function, Readmission and Mortality in the PPS.* *(page 11)*

- Supplementary Figure 2: *Subgroup Analysis of Multidisciplinary Intervention on All-Cause Readmission.* *(page 12)*

Supplementary Methods ………….. page 13

- Appendix 1: *Standard Operating Procedure (SOP) for Comprehensive Multidisciplinary Programme (RENAP) for Frail Elderly Patients* *(page 13)*
- Appendix 2: *Manual to Record Daily Exercise, Meal Intake, and Acupoint Massage Activities* *(page 30)*
- Appendix 3: *Clinical Research Protocol* *(page 36)*
- Appendix 4: *Statistical Analysis Plan* *(page 49)*
- References *(page 54)*

Supplementary Tables

Supplementary Table 1: Baseline Characteristics of Patients in the PPS

	Overall (*n*=252)	Control group (*n*=132)	Intervention group (*n*=120)
**Demographic characteristics**			
Age, years	79.5±5.5	79.4±5.3	79.7±5.7
Male, %	104(41.3)	50(37.9)	54(45.0)
BMI, kg/m^2	25.2±3.5	24.7±3.3	25.6±3.7
Widowed, %	34(13.6)	17(12.9)	17(14.3)
Living alone, %	33(13.1)	18(13.6)	15(12.5)
Mental work, %	169(67.3)	90(68.2)	79(66.4)
History of smoking, %	57(22.7)	26(19.7)	31(26.1)
**Education level**			
Primary school or below, %	40(15.9)	22(16.7)	18(15.1)
junior high school, %	70(27.9)	35(26.5)	35(29.4)
senior high school, %	61(24.3)	33(25.0)	28(23.5)
junior college or above, %	80(31.9)	42(31.8)	38(31.9)
**Clinical diseases**			
Hypertension, %	213(84.5)	112(84.9)	101(84.2)
Coronary artery disease, %	180(71.4)	97(73.5)	83(69.2)
Atrial fibrillation or flutter, %	59(23.5)	33(25.0)	26(21.9)
Heart failure, %	68(27.0)	35(26.5)	33(27.5)
Diabetes mellitus, %	104(41.3)	59(44.7)	45(37.5)
Chronic kidney disease, %	37(14.7)	23(17.4)	14(11.7)
Malignancy, %	31(12.3)	17(12.9)	14(11.7)
Polypharmacy (≥5 medications), %	192(76.2)	99(75.0)	93(77.5)
**Comprehensive geriatric assessments**			
Fried Frailty Phenotype score	3.5±0.6	3.6±0.7	3.5±0.6
Handgrip Strength, kg	18.6±6.9	18.1±6.6	19.3±7.1
Gait speed, m/s	0.6±0.2	0.6±0.2	0.6±0.2
Unintentional weight loss, %	64(25.4)	34(25.8)	30(25.0)
Physical exhaustion, %	219(86.9)	115(87.1)	104(86.7)
Low physical activity, %	186(73.8)	100(75.8)	86(71.7)
SPPB	6.3±2.7	6.4±2.6	6.3±2.7
IADL	6.3±2.1	6.2±2.2	6.4±2.0
CFS	4.8±0.8	4.8±0.8	4.8±0.8
HADS-A	2.3±3.1	1.9±2.2	2.7±3.9
NRS2002	1.7±0.9	1.7±0.8	1.7±0.9
PSQI	9.0±4.0	9.2±3.8	8.8±4.3
**Admission laboratory values**			
Glycosylated hemoglobin, %	6.6±1.2	6.5±1.1	6.8±1.3
White blood cell, ×10^9/L	6.1±1.9	6.1±2.2	6.1±1.6
Albumin, g/L	38.9±3.4	38.8±3.5	39.0±3.4

Creatine, umol/L	86.9±40.3	90.1±47.9	83.4±29.9
Total cholesterol, mmol/L	3.7±0.9	3.7±1.0	3.6±0.8
Triglyceride, mmol/L	1.3±0.8	1.2±0.7	1.3±0.8
hs-CRP, mg/L	3.7±6.3	2.5±5.3	5.4±7.4
NT-proBNP, pg/mL	387.1 (147.9, 1080.0)	416.6 (144.6, 1048.5)	387.1 (152.8, 1152.0)
LVEF, %	59.1±8.8	59.7±7.8	58.5±9.8

Values are mean ± SD, n (%), or median (interquartile range). Percentages were calculated using the number of participants with available data for each variable as the denominator, unless otherwise specified. *n* shown in the column headings represent the total number of participants included in the per-protocol set.

BMI = body mass index; CFS = Clinical Frailty Scale; hs-CRP = high-sensitive C-reactive protein; HADS-A = Hospital Anxiety and Depression Scale-anxiety; IADL = Instrumental Activities of Daily Living; LVEF = left ventricular ejection fraction; NRS2002 = Nutrition Risk Screening-2002; NT-proBNP = N-terminal pro-B-type natriuretic peptide; PPS = Per-Protocol Set; PSQI = Pittsburgh Sleep Quality Index; SPPB = Short Physical Performance Battery.

Supplementary Table 2: Comparison of Characteristics Between Participants Who Completed Follow-up and Those Lost to Follow-up

	Completed Follow-up (n=252)	Lost to Follow-up (n=81)	Statistic(degrees of freedom)	P value
Age, years	79.5±5.5	79.5±5.4	t(331)= 0.074	0.941
Male, %	104(41.3)	38(46.9)	$\chi^2(1)$ = 0.798	0.372
BMI, kg/m^2	25.2±3.5	24.6±3.6	t(331)= 1.123	0.262
Hypertension, %	213(84.5)	65(80.2)	$\chi^2(1)$ = 0.813	0.367
Coronary artery disease, %	180(71.4)	53(65.4)	$\chi^2(1)$ = 1.049	0.306
Atrial fibrillation or flutter, %	59(23.5)	31(38.3)	$\chi^2(1)$ = 6.862	0.009
Heart failure, %	68(27.0)	21(25.9)	$\chi^2(1)$ = 0.035	0.851
Diabetes mellitus, %	104(41.3)	27(33.3)	$\chi^2(1)$ = 1.618	0.203
Chronic kidney disease, %	37(14.7)	12(14.8)	$\chi^2(1)$ = 0.001	0.977
Malignancy, %	31(12.3)	4(4.9)	$\chi^2(1)$ = 3.533	0.060
Fried Frailty Phenotype score	3.5±0.6	3.5±0.7	t(331)= -0.134	0.893
SPPB	6.3±2.7	5.5±2.8	t(331)= 2.307	0.022

Values are mean ± SD or n (%). *n* represents the number of participants in each group. Continuous data were analysed using two-sided *t*-test and categorical data were analysed using two-sided chi-square test. No adjustments were made for multiple comparisons.

BMI = body mass index; SPPB = Short Physical Performance Battery.

Supplementary Table 3: Comparison of Characteristics Among Participants Lost to Follow-up in the Intervention and Control Groups

	Control group (*n*=35)	Intervention group (*n*=46)	Statistic(degrees of freedom)	*P* value
Age, years	78.5±5.9	80.2±4.9	t(79) = -1.404	0.164
Male, %	19(54.3)	19(41.3)	$\chi^2(1)$ = 1.345	0.246
BMI, kg/m^2	24.7±3.4	24.6±3.1	t(79) = 0.193	0.848
Hypertension, %	27(77.1)	38(82.6)	$\chi^2(1)$ = 0.375	0.540
Coronary artery disease, %	23(65.7)	30(65.2)	$\chi^2(1)$ = 0.002	0.963
Atrial fibrillation or flutter, %	13(37.1)	18(39.1)	$\chi^2(1)$ = 0.033	0.855
Heart failure, %	11(31.4)	10(21.7)	$\chi^2(1)$ = 0.972	0.324
Diabetes mellitus, %	7(20.0)	20(43.5)	$\chi^2(1)$ = 4.930	0.026
Chronic kidney disease, %	6(17.1)	6(13.0)	$\chi^2(1)$ = 0.265	0.607
Malignancy, %	1(2.9)	3(6.5)	$\chi^2(1)$ = 0.056	0.813
Fried Frailty Phenotype score	3.4±0.6	3.6±0.7	t(79) = -1.197	0.235
SPPB	5.6±2.6	5.5±2.9	t(79) = 0.274	0.785

Values are mean ± SD or n (%). *n* represents the number of participants in each group. Continuous data were analysed using two-sided *t*-test and categorical data were analysed using two-sided chi-square test. No adjustments were made for multiple comparisons.

BMI = body mass index; SPPB = Short Physical Performance Battery.

Supplementary Table 4: Estimated Between-group Adjusted Mean Differences in Change From Baseline to 12 Months in the PPS

Outcome	Baseline		1-year follow-up		Least-square mean change from baseline to 1 year		Estimated adjusted difference in mean change at 1-year endpoint (95% CI)	Statistic(degrees of freedom)	*P* value
	Control group	Intervention group	Control group	Intervention group	Control group	Intervention group			
FFP	3.6±0.7	3.5±0.6	2.7±1.3	1.7±1.3	-0.9±0.1	-1.8±0.1	-0.90 (-1.27 to -0.52)	t(750) = -4.67	<0.001
SPPB	6.4±2.6	6.3±2.7	5.0±2.8	7.7±3.6	-1.4±0.4	1.4±0.4	2.78 (1.65 to 3.90)	t(750) = 4.85	<0.001
IADL	6.2±2.2	6.4±2.0	5.3±2.5	6.6±2.1	-1.0±0.2	0.2±0.2	1.20 (0.67 to 1.74)	t(544) = 4.43	<0.001
CFS	4.8±0.8	4.8±0.8	5.0±1.0	4.3±1.2	0.2±0.1	-0.4±0.1	-0.68 (-0.93 to -0.43)	t(542) = -5.32	<0.001
HADS-A	1.9±2.2	2.7±3.9	3.0±2.8	2.4±2.8	1.0±0.3	-0.3±0.4	-1.38 (-2.35 to -0.42)	t(377) = -2.82	0.005

Values are mean ± standard deviation otherwise indicated. Least-square mean changes from baseline to 1-year follow-up and estimated adjusted differences at 1-year endpoint were obtained from two-sided mixed-effects models. Least-square mean changes are presented as estimated change ± SE, and estimated differences are presented with 95% CIs. All reported *P* values for secondary outcomes are nominal and have not been adjusted for multiple comparisons.

CFS = Clinical Frailty Scale; CI = confidence interval; FFP = Fried Frailty Phenotype; HADS-A = Hospital Anxiety and Depression Scale-anxiety; IADL = instrumental activities of daily living; SPPB = Short Physical Performance Battery.

Supplementary Table 5: Between-Group Differences in some secondary outcomes at 1-Year Follow-Up

	Control group	Intervention group	Statistical value	*P* value
BADL	5.5(4, 6)	6(5, 6)	Z = -3.348	0.001
NRS2002	1(1, 1)	1(1, 1)	Z = 0.099	0.921
Medical insurance expenses, RMB	18077.0 (11292.0, 29698.0)	18450.5(10830.0, 26089.0)	Z = -0.094	0.925

Values are median (interquartile range). Between-group comparisons were performed using two-sided Wilcoxon rank-sum tests. No adjustments were made for multiple comparisons.

BADL = Basic Activities of Daily Living; NRS2002 = Nutrition Risk Screening-2002.

Supplementary Table 6: Cox proportional hazards regression model analysis for all-cause readmission and mortality

	Readmission			Mortality		
	Statistic(degrees of freedom)	HR (95% CI)	*P* value	Statistic(degrees of freedom)	HR (95% CI)	*P* value
Age (years)						
<80	-	-	-	-	-	-
≥80	Wald $\chi^2(1)$ = 0.56	0.81 (0.47 to 1.40)	0.455	Wald $\chi^2(1)$ = 0.16	1.26 (0.40 to 3.95)	0.694
Sex (ref = male)	Wald $\chi^2(1)$ = 0.07	1.07 (0.62 to 1.87)	0.799	Wald $\chi^2(1)$ = 4.22	0.32 (0.11 to 0.95)	0.040
BMI, kg/m^2						
<18	-	-	-	-	-	-
18-24	Wald $\chi^2(1)$ = 0.10	1.39 (0.18 to 10.53)	0.752	Wald $\chi^2(1)$ = 5.95	0.11 (0.02 to 0.65)	0.015
>24	Wald $\chi^2(1)$ = 0.01	0.88 (0.12 to 6.73)	0.905	Wald $\chi^2(1)$ = 11.44	0.04 (0.007 to 0.26)	<0.001
Coronary artery disease (yes vs no)	Wald $\chi^2(1)$ = 0.05	1.07 (0.58 to 1.97)	0.826	Wald $\chi^2(1)$ = 4.94	3.68 (1.17 to 11.59)	0.026
Atrial fibrillation or flutter (yes vs no)	Wald $\chi^2(1)$ = 0.24	0.86 (0.47 to 1.57)	0.621	Wald $\chi^2(1)$ = 0.93	0.57 (0.18 to 1.78)	0.334
Heart failure (yes or no)	Wald $\chi^2(1)$ = 0.07	0.92 (0.50 to 1.70)	0.795	Wald $\chi^2(1)$ = 6.23	0.24 (0.08 to 0.73)	0.013
Diabetes mellitus (yes vs no)	Wald $\chi^2(1)$ = 0.13	1.11 (0.63 to 1.97)	0.716	Wald $\chi^2(1)$ = 0.88	0.58 (0.19 to 1.81)	0.347
RENAP intervention (yes vs no)	Wald $\chi^2(1)$ = 4.75	0.53 (0.30 to 0.94)	0.029	Wald $\chi^2(1)$ = 1.14	0.53 (0.16 to 1.70)	0.286

Hazard ratios (HR) and 95% confidence intervals (CI) were estimated from two-sided Cox proportional hazards models. No adjustments were made for multiple comparisons.

BMI = body mass index; RENAP = Rehabilitation, Education, Nutritional Guidance, Acupoint Massage, Polypharmacy Management.

Supplementary Table 7: New Falls Occurring during each Follow-up period

Follow-up period	Control group	Intervention group	Statistic(degrees of freedom)	*P* value
0-3 months	6(5.5)	5(6.8)	$\chi^2(1) = 0.133$	0.715
3-6 months	10(11.4)	15(16.9)	$\chi^2(1) = 1.100$	0.294
6-12 months	13(9.9)	8(6.4)	$\chi^2(1) = 1.018$	0.313

Values are n (%). Percentages were calculated using the number of participants with available follow-up data at each time period as the denominator. Between-group comparisons at each time point were performed using two-sided chi-square tests. No adjustments were made for multiple comparisons.

Supplementary Figures

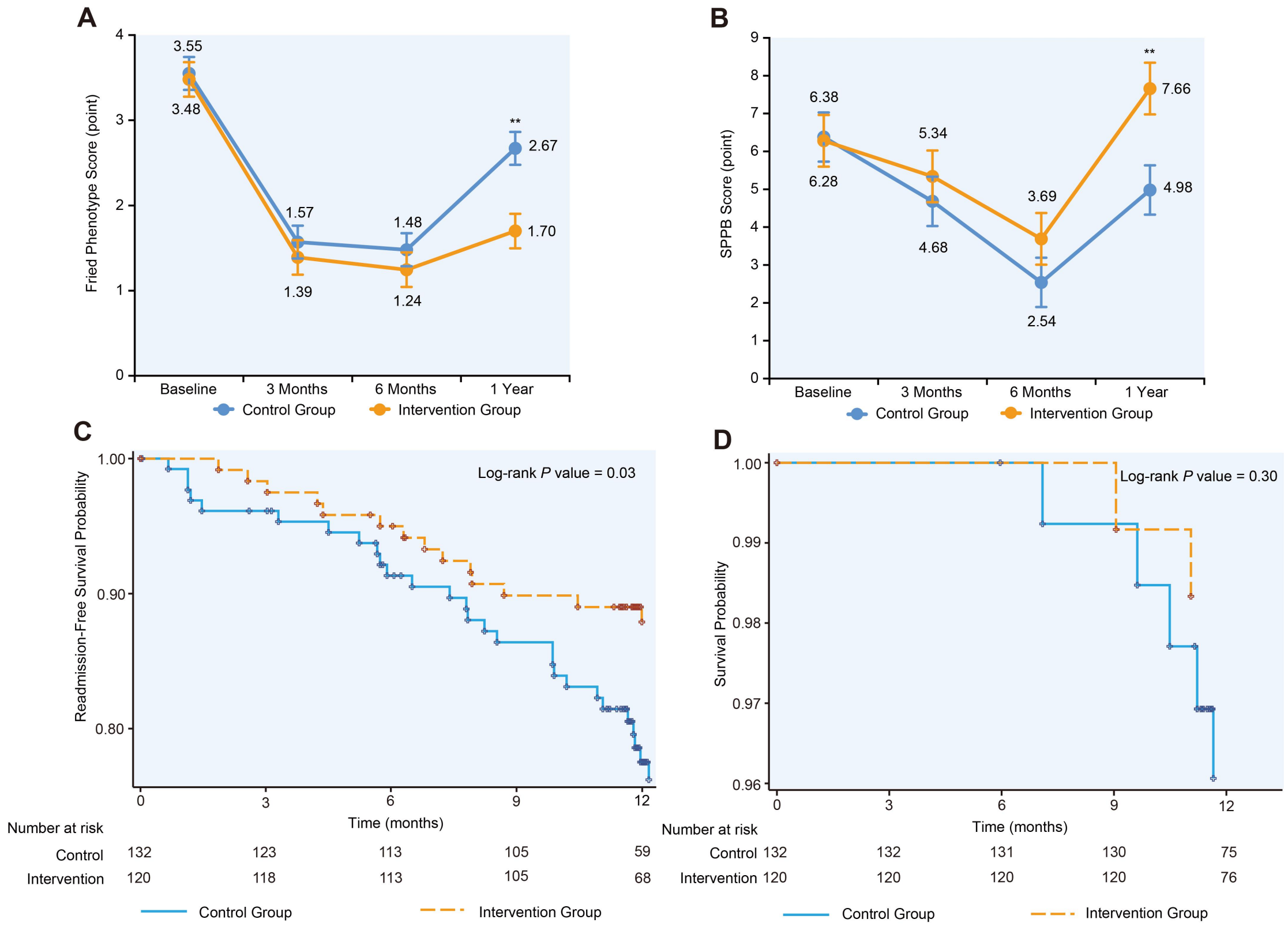

Supplementary Figure 1: Impact of Multidisciplinary Intervention on Frailty, Physical Function, Readmission and Mortality in the per-protocol set (PPS)
Panels A and B show changes in Fried Frailty Phenotype (FFP) and Short Physical Performance Battery (SPPB) scores from baseline to 12 months. Data are presented as least-square mean ± 95% confidence interval estimated from two-sided mixed-effects models using all available data from the per-protocol population (*n*=252 participants). The between-group adjusted mean differences in FFP and SPPB at 12 months were -0.90 [t(750) = -4.67; $P < 0.001$; 95% CI: -1.27 to -0.52] and 2.78 [t(750) = 4.85; $P < 0.001$; 95% CI: 1.65 to 3.90]. Panels C and D show Kaplan-Meier curves for all-cause readmission and overall mortality, compared using two-sided log-rank tests [$\chi^2(1) = 4.70$, $P = 0.030$ for readmission; $\chi^2(1) = 1.07$, $P = 0.302$ for mortality). The number of participants at risk at each time point is shown in the tables below the curves. For all panels, blue and orange lines represent the control and intervention groups, respectively, and no adjustments were made for multiple comparisons. Source data are provided as a Source Data file. **$P < 0.001$.

Subgroup	Interventional group no.of event/no.of participant (%)	Control group no.of event/no.of participant (%)	Hazard Ratio (95%CI)	P-value
**Overall**	**20/166(12.05)**	**36/167(21.56)**	**0.53(0.31,0.91)**	**0.02**
**Age**				
≥80 yr	10/84(11.90)	16/84(19.05)	0.59(0.27,1.30)	0.19
<80 yr	10/82(12.20)	20/83(24.10)	0.48(0.22,1.02)	0.06
**Sex**				
Female	12/93(12.90)	22/98(22.45)	0.59(0.29,1.20)	0.15
Male	8/73(10.96)	14/69(20.29)	0.45(0.19,1.07)	0.07
**BMI**				
BMI>24	13/112(11.61)	17/92(18.48)	0.61(0.30,1.26)	0.18
18≤BMI≤24	6/49(12.24)	19/72(26.39)	0.44(0.18,1.11)	0.08
**Heart Failure**				
Yes	6/43(13.95)	9/46(19.57)	0.62(0.22,1.75)	0.37
No	13/121(10.74)	27/119(22.69)	0.46(0.24,0.90)	0.02
**CAD**				
Yes	15/113(13.27)	23/120(19.17)	0.67(0.35,1.27)	0.22
No	4/51(7.84)	13/45(28.89)	0.24(0.08,0.75)	0.01
**AF/Afl**				
Yes	5/44(11.36)	11/46(23.91)	0.46(0.16,1.32)	0.15
No	14/119(11.76)	25/119(21.01)	0.53(0.27,1.02)	0.06
**Diabetes**				
Yes	10/65(15.38)	10/66(15.15)	1.07(0.45,2.57)	0.88
No	9/99(9.09)	26/99(26.26)	0.30(0.14,0.64)	0.002
**FRIED**				
≤3	7/93(7.53)	21/93(22.58)	0.30(0.13,0.72)	0.01
≥4	13/73(17.81)	15/74(20.27)	0.87(0.42,1.83)	0.72

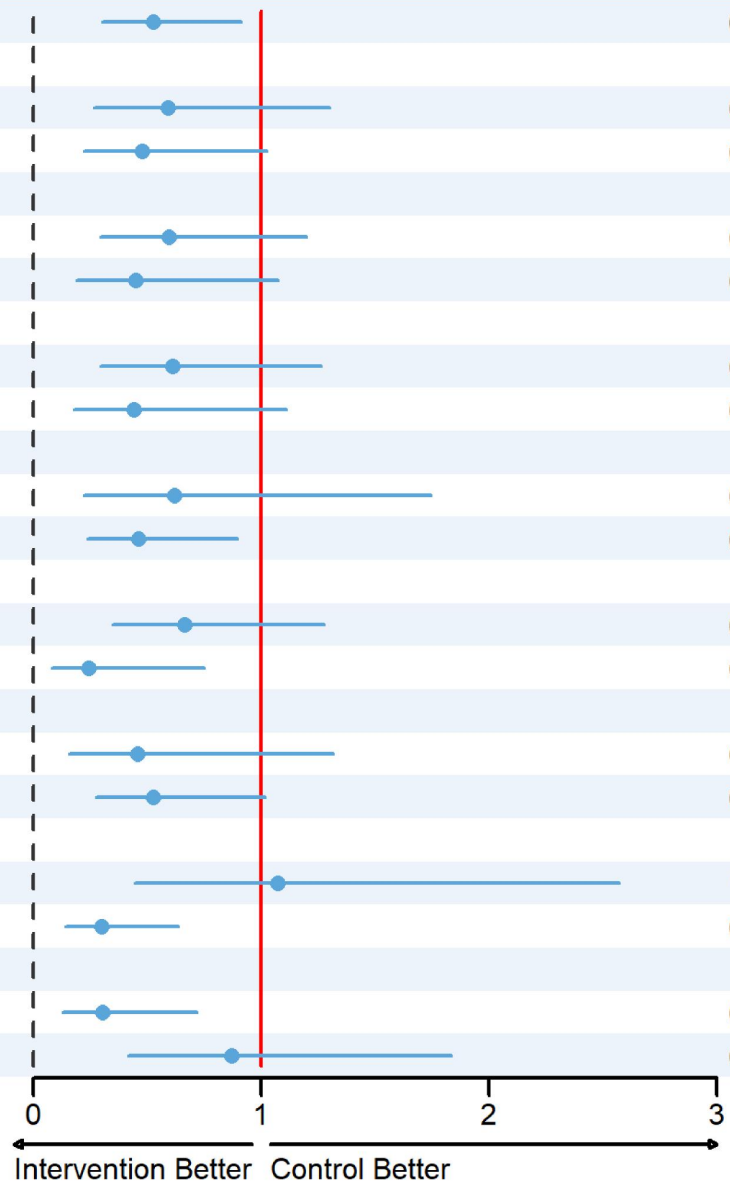

Supplementary Figure 2: Subgroup Analysis of Multidisciplinary Intervention on All-Cause Readmission

Forest plots show the hazard ratios of the multidisciplinary intervention on all-cause readmission across pre-specified subgroups. Estimates were derived from two-sided univariable Cox proportional hazards models. Subgroups included variables such as age, sex, body mass index (BMI), heart failure, coronary artery disease (CAD), atrial fibrillation/flutter (AF/Afl), diabetes and Fried Frailty Phenotype score. Dots represent point estimates of the hazard ratio; horizontal lines indicate 95% confidence intervals. The solid vertical line at 1 indicates no difference between groups. All subgroup analyses were exploratory and were not adjusted for multiple comparisons. Source data are provided as a Source Data file.

Supplementary Methods

Appendix 1. Standard Operating Procedure (SOP) for comprehensive multidisciplinary programme (RENAP) for Frail Elderly Patients

The operating procedure primarily applies to the project 'The Multidisciplinary Management Model on Frail Elderly Patients: A Randomised, Single-Blind, Parallel-Group, Multicentre Clinical Study'. It is designed to ensure the quality of the study across all participating research units and to guarantee that all subjects in the intervention group receive standardised interventions according to uniform criteria.

1. REHABILITATION EXERCISE

Rehabilitation physicians will teach a set of rehabilitation exercises, including strength training, flexibility, balance, and agility. In addition, patients will undergo individualised aerobic exercise training tailored to their personal interests.

1）Precautions

① Frail elderly individuals should perform exercises under the supervision of a family member or caregiver.

② Subjects should wear durable, non-slip shoes during ground-based training.

③ Ground exercises must be performed on a flat surface, with some exercises requiring support from a nearby wall.

④ Certain exercises may require the use of a chair. Ensure the chair is sturdy and stable, with the backrest positioned against a wall or placed on a carpet.

⑤ Breathing should be coordinated with the exercises, avoiding breath-holding.

⑥ Training should not be excessive; no pain should be felt during the exercises.

⑦ Avoid rapid movements; adhere to the principles of slow stretching and gradual relaxation.

⑧ If any discomfort occurs during the exercise, stop immediately. If discomfort persists after resting, seek medical attention promptly.

⑨ The recommended number of repetitions can be adjusted based on activity endurance, perceived fatigue, and interest. If exceeding the recommended range, consult a physician.

2）Basic Exercises

① Posture

Standing Posture 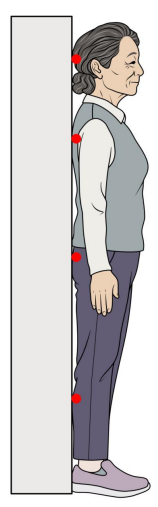	Keep your chest out and head up. Stand as straight and tall as possible.
Sitting Posture 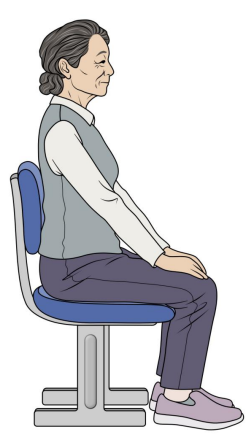	Sit on a chair, avoiding overly wide or soft sofas. Keep your buttocks close to the back of the chair, with your upper body relaxed against the chair back. Avoid jutting your head forward.

② Breathing Pattern Guidance

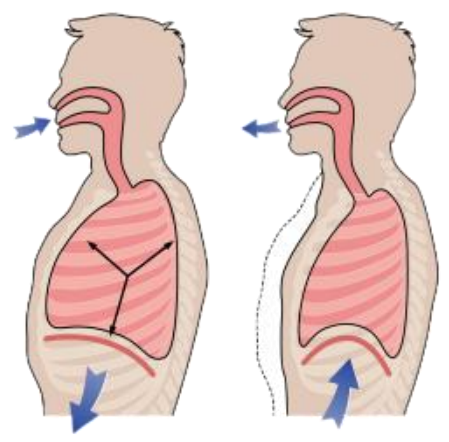	During inhalation, fully engage the diaphragm, allowing for even expansion of the entire chest and abdominal area (engaging the lower lungs). During exhalation, do the opposite.

③ Abdominal Tension. Keep your abdominal muscles slightly tensed (pushed outward) at all times.

④ Pelvic Floor Exercises. Start practicing in a lying position. Once you can perform the exercises successfully, progress to sitting and even standing positions.

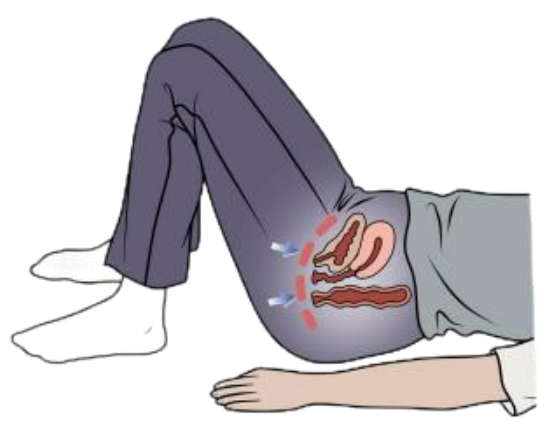	Strong and Quick Contractions: Contract your pelvic floor muscles with maximum strength for 3 seconds, then relax for 3 seconds. Perform 30 repetitions per set, 2-3 sets daily.<br>Sustained Contractions: Contract your pelvic floor muscles with 1/2 to 1/3 of your maximum strength, maintaining the contraction for as long as possible, aiming for 1 minute. Relax for 30 seconds between contractions. Perform 3 one-minute contractions per set, 2-3 sets daily. Ensure that your abdomen and limbs remain relaxed during the pelvic floor contractions,

	without participating in the movement.

3）Numbered Exercises

(1)Neck Muscle Exercises 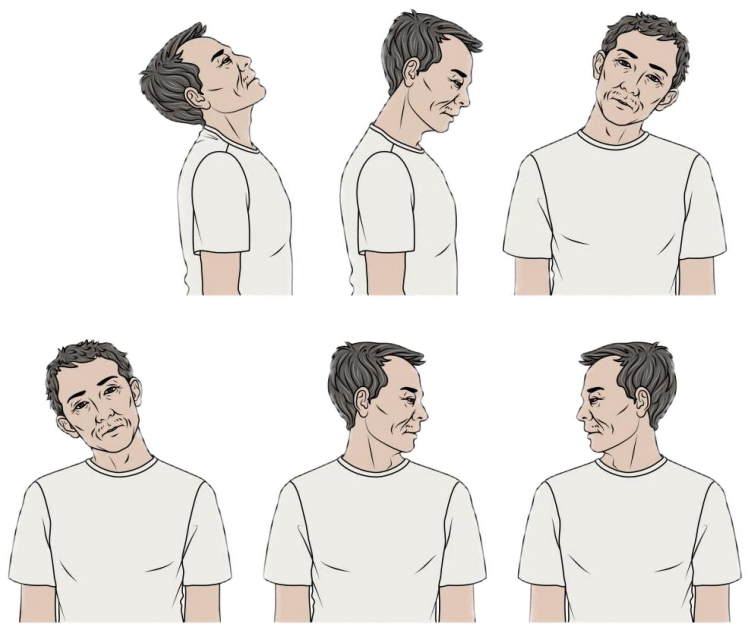	The subject stands on the ground with feet shoulder-width apart, arms at their sides. Perform head movements as follows: Look up and down 5 times each, holding each position for 5 seconds, trying to reach the maximum range.Tilt head to the left and right 5 times each. Rotate head to the left and right 5 times each, ensuring the shoulders do not rotate with the head. Avoid rapid movements and simultaneous up-and-down or side-to-side movements. To increase the difficulty, the range of head rotation, holding time, and number of repetitions can be increased.
(2)Seated Forward Arm Extension with Resistance Band	Sit on a straight-backed chair with the resistance band placed behind the shoulders. Hold both ends of the resistance band, adjusting it to a personal tolerance length (the endpoint of the stretch should

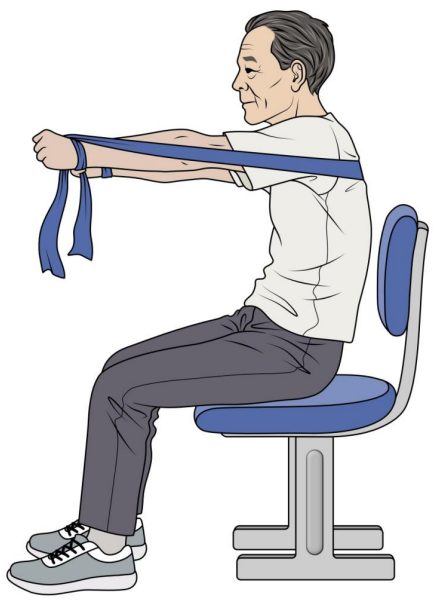	feel challenging but not overly difficult). Perform forward stretching movements, ensuring both the forward and return movements are done slowly to avoid quick rebound. Perform 5-20 repetitions. To increase difficulty, gradually transition to a standing position, increase the resistance of the band, or increase the number of repetitions. Stop if you feel fatigued during the initial training.
(3)Behind-the-Back Hand Clasp Stretch 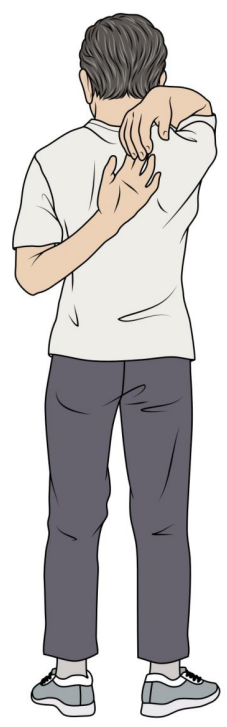	Stand with both feet on the ground. One hand reaches down from the shoulder, while the other hand reaches up from the back towards the centre of the back, trying to make the hands meet (reach as close to your limit as possible). Then, expand the chest and pull the shoulders and elbows backward, maintaining the stretch for 8 seconds. Alternate sides, performing 2 sets for each side. Difficulty can be increased by appropriately adding repetitions and extending the holding time.
(4) Lunge Stretch	Stand with both feet on the ground. Step one leg forward into a lunge position, keeping a stable stance. Place both hands, one on top of the other, on

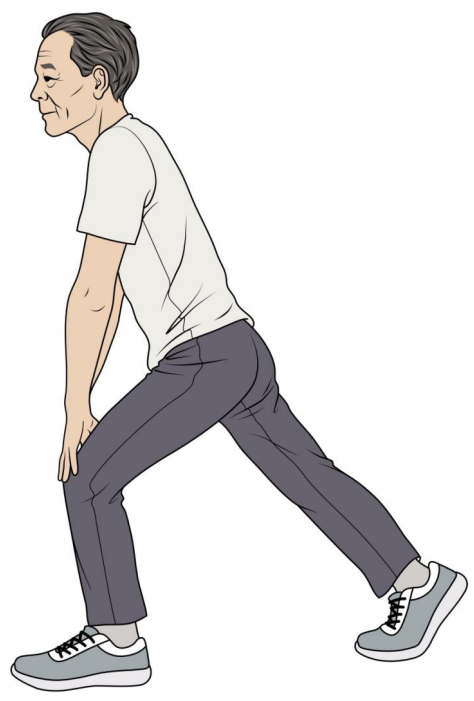	the knee of the front leg and press down, stretching the front of the thigh of the back leg. Maintain the stretch for 8 seconds. Alternate sides, performing 2 sets for each side, gradually increasing to 5 sets. Increase difficulty by widening the distance between the legs and increasing the number of presses and stretches.
(5) Heel Raise Training<br>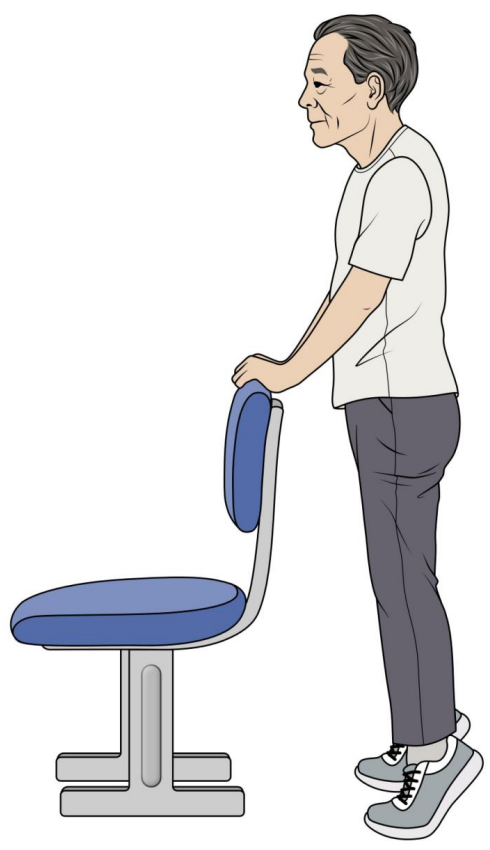	Stand on a flat surface with stable furniture or a wall nearby for support. Initially, distribute weight evenly on both feet and start with a low heel raise, gradually increasing the difficulty by raising the heels higher and shifting the weight to one leg. Alternate sides and practice single-leg heel raises. Ensure to avoid falling during the exercise. The ultimate goal is to perform 20 heel raises on each side. Begin the training with 1-2 heel raises.
(6) Marching in Place<br>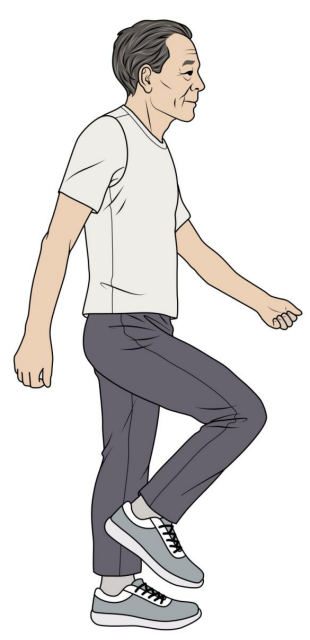	Start each step at a comfortable height, gradually raising each knee to the midpoint between the patella and the iliac crest. Begin with a duration of 30 seconds, gradually increasing the time as tolerated. It is not recommended to exceed 10 minutes.

(7) Seated Toe Touch 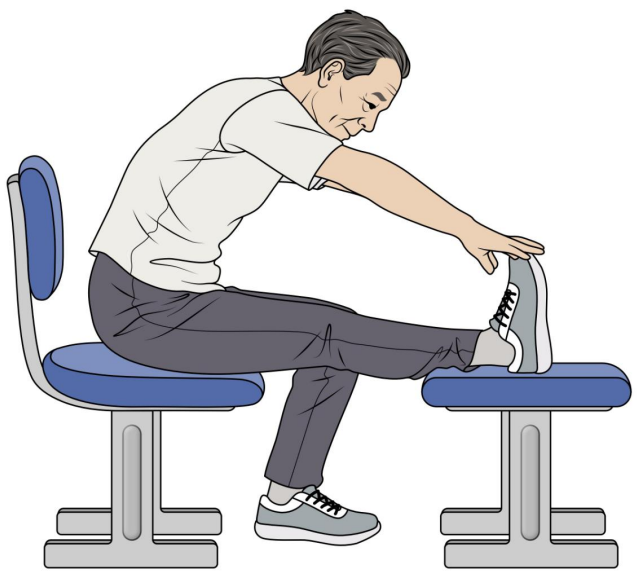	Sit on the front edge of a chair with one leg bent and the other leg extended straight, foot flexed upward at a 90° angle. Reach forward forcefully to touch your toes, maintaining the position for at least 2 seconds. At the end of the movement, hold the stretch for 8 seconds. Alternate sides, performing 2 sets for each side, gradually increasing to 8 sets per side.

4）Aerobic Exercise

This activity should be chosen according to personal preferences. Start with a small amount of exercise initially, gradually increasing the training volume. If the exercise intensity is low, aim to perform aerobic exercise daily for 30 minutes to 1 hour. If the exercise intensity reaches a moderate level, aim for 3-5 sessions per week, with each session lasting at least 30 minutes.

5）Follow-up

Each subject will be provided with a rehabilitation training logbook. Based on assessments conducted at 3 and 6 months, including the subject's adherence to rehabilitation training, activity fatigue, and tolerance, recommendations will be made to either increase, maintain, or reduce the intensity of the rehabilitation exercises.

2. PATIENT EDUCATION

One of the significant reasons for the increased severity of conditions in frail elderly patients is their lack of knowledge and skills in self-management. Education can enhance patients' self-management abilities and medication adherence, helping them improve their lifestyle. The primary content covers basic knowledge of frailty, fall prevention education, first aid education, medication therapy and adherence, dietary guidance, and lifestyle interventions.

1）Basic Knowledge of Frailty

Frailty is a nonspecific state resulting from a decline in physiological reserves, leading to increased vulnerability and reduced stress resilience in elderly individuals. It is a core aspect of geriatric syndromes and a precursor to disability, significantly impacting the function and quality of life of elderly individuals and posing a major threat to healthy life expectancy. The prevalence of frailty increases with age, and compared to non-frail elderly individuals, those who are frail have a higher average risk of mortality. Frail elderly individuals are more likely to experience adverse clinical events and consume a substantial amount of healthcare resources compared to their healthier counterparts. Frailty is strongly associated with poor individual prognosis and is an independent predictor of falls, reduced mobility, functional deterioration, hospitalisation, and death. Early identification of frailty and effective interventions can halt, reduce, delay, or reverse the severity of frailty, preventing or mitigating its adverse health outcomes. This would bring immense benefits to frail elderly individuals, their families, and society as a whole.

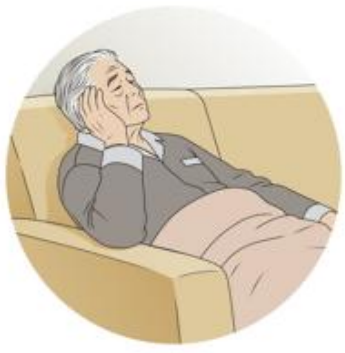	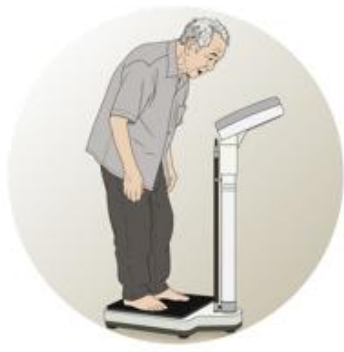	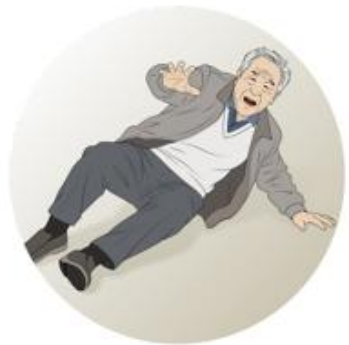	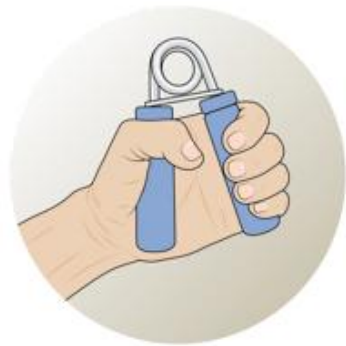	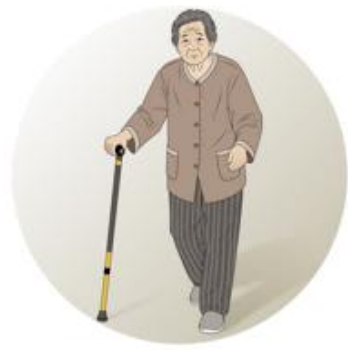
**Exhausion**	**Unintentional Weight Loss**	**Low Physical Activity**	**Weakness (Grip Strength)**	**Slowness (Walking Speed)**

2）Fall Prevention Education

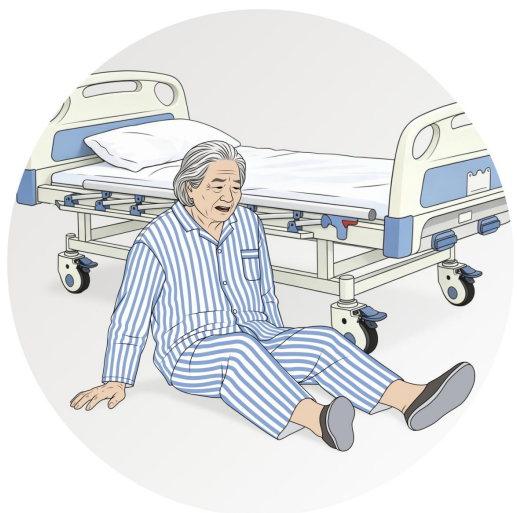	For patients with limited mobility, weakness, impaired vision, or disorientation, it is recommended to minimize activity. When necessary, please seek assistance from a nurse or family member. During essential activities, family members should accompany and assist the patient.
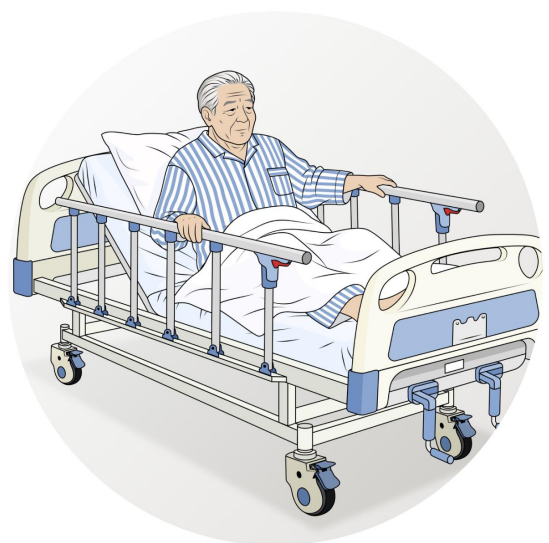	When the bed rails are raised, please ask a nurse or family member to lower the bed rails before getting out of bed. Do not attempt to bend over to remove the bed rails yourself or climb over the bed rails directly.
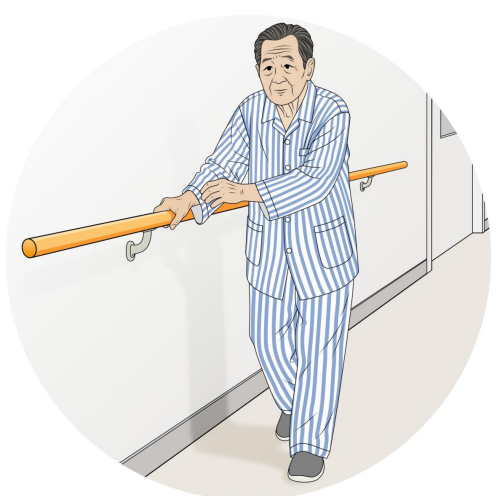	When taking sedatives, diuretics, antihypertensives, antidiabetic medications, or experiencing dizziness and fatigue, reduce activities and lie down to rest as soon as possible. Use the call button to request assistance from a nurse for any physiological needs. In the hallway, use handrails for walking or resting as needed.

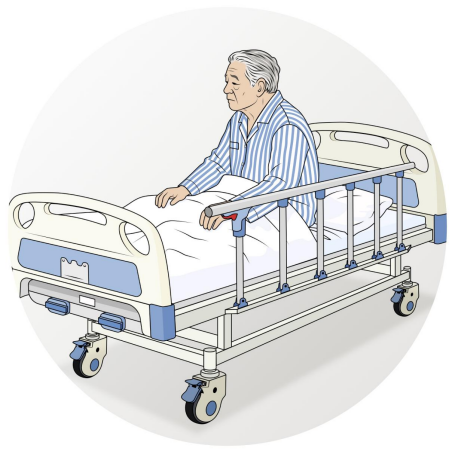	Upon waking, remain lying down for a moment, then sit up slowly, and finally stand. Avoid getting up directly from a lying position.
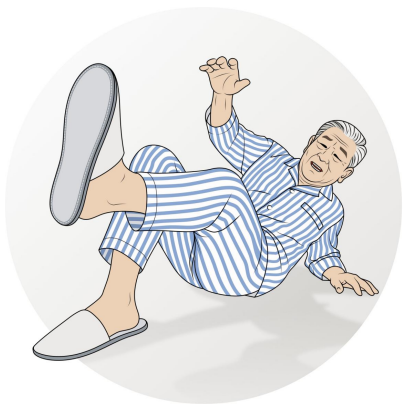	Wear non-slip shoes and avoid wearing slippers when going out. Disposable slippers are strictly prohibited.
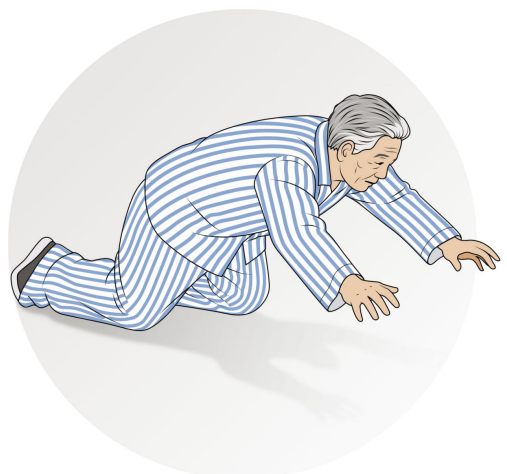	Wear well-fitting clothing that is not too long or too loose to avoid tripping. If necessary, roll up your pant legs.
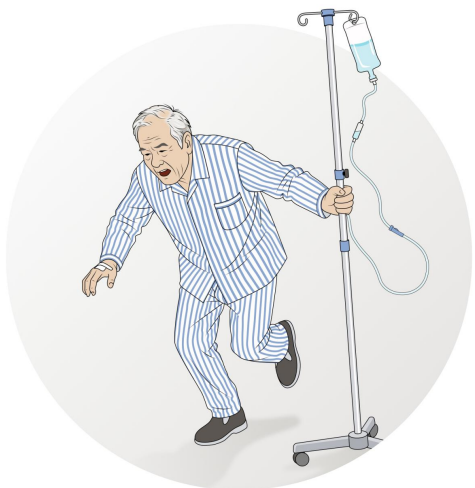	Use walking aids and keep them within easy reach. Do not use IV poles, wheelchairs, or other unstable objects as walking aids.
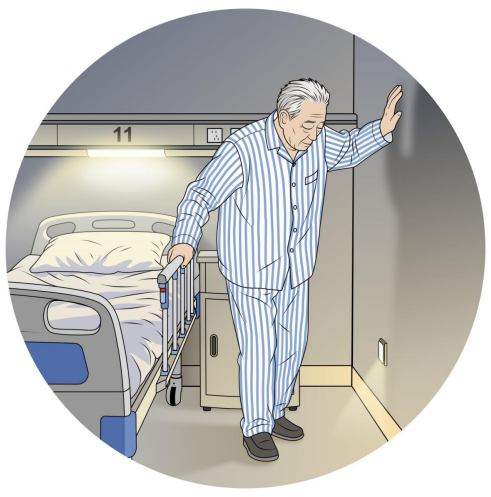	Use a bedside lamp at night for safe walking. Avoid walking in the dark to prevent tripping.
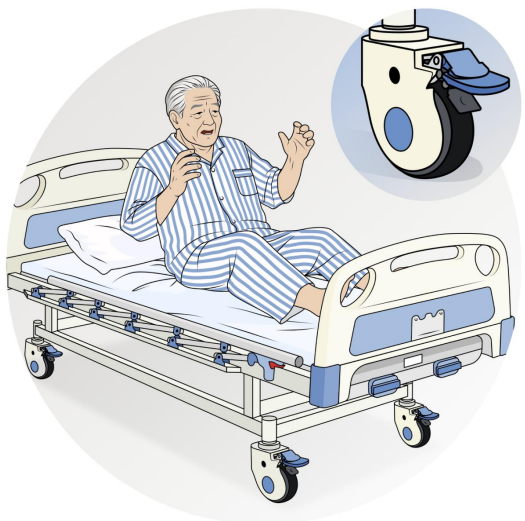	Do not disengage the hospital bed brake lock; keep the bed unit stable and secure.

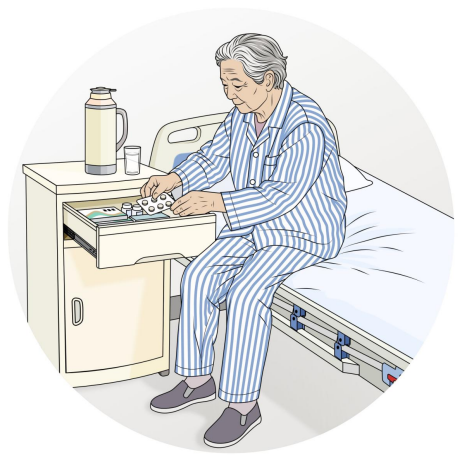	Store personal items in the cabinet to maintain clear pathways. Ask a nurse to store luggage and folding beds. Place essential items within easy reach.
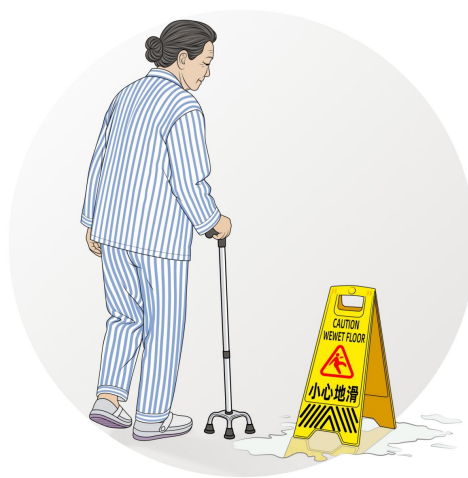	Keep the floor dry. If the floor is wet, promptly ask a nurse to handle it. Avoid walking through areas with wet floor warning signs.
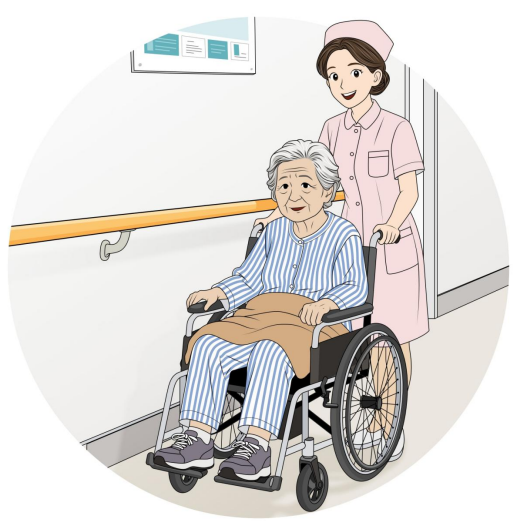	When patients go out for examinations, they should wear non-slip athletic shoes and are prohibited from wearing slippers. Patients with limited mobility should be accompanied by a designated person and wear a high-risk fall identification tag. If necessary, use a wheelchair for assistance.

3）Psychological and Mental Guidance

Frail elderly individuals are more likely to experience unique psychosocial conditions, such as dependency on others, cognitive impairment, rigidity, living alone, loneliness, social isolation, and depression. These factors can negatively impact their ability to maintain a healthy diet and exercise routine. It is recommended that patients maintain a positive and optimistic mindset and that psychological support be provided to help them cope with these challenges.

3. NUTRITIONAL GUIDANCE

The nutritionist will assess the subject's basic condition, nutritional-related laboratory indicators, body composition analysis, and conduct a 24-hour dietary recall to evaluate the patient's previous dietary intake. Based on this assessment, a nutritional prescription will be formulated and monitored for adherence.

1）Nutritional Prescription

The nutritionist will recommend the daily total energy intake (kcal/day), macronutrient intake (g/day), and energy distribution (%) based on the subject's sex, age, height, weight, medical history, and laboratory results. Dietary recommendations will be tailored according to the patient's eating habits. If the patient experiences poor appetite or difficulty eating during dietary interventions, nutritional supplements may be recommended.

2）Recommended Intake

①Energy. The target energy intake is 20-30 kcal/kg/day, with reductions during acute phases and increases during recovery phases. For underweight elderly patients, calculate based on 120% of actual body weight; for obese elderly patients, calculate based on ideal body weight [calculated as (height in cm - 105) kg].

②Protein. The target protein intake is 1.0-1.5 g/kg/day, with more than 50% being high-quality protein (whey protein, casein, and soy protein). A high-protein diet is recommended during the disease recovery period. For patients with chronic kidney disease not on replacement therapy, the target protein intake should be 0.6-0.8 g/kg/day, emphasizing the supplementation of high-quality proteins.

③Carbohydrates. carbohydrates make up 50%-65% of total energy intake for healthy individuals. Adjustments may be made based on the disease state.

④Fats. recommends that fats should not exceed 35% of total energy intake, with saturated fatty acids comprising less than 10% of total energy. Polyunsaturated fatty acids, which provide essential fatty acids, should account for 6%-11% of total energy, with a focus on increasing the proportion of monounsaturated fatty acids.

⑤Dietary Fiber. The recommended intake is 25-30 g/day.

Recommended Daily Food Content and Quantities					
Grains (g)	g	Eggs (g)	g	Milk (ml)	ml
Lean Meat (g)	g	Vegetables (g)	g	Fruits (g)	g
Cooking Oil (g)	g				

Food Exchange Portions
25g rice/millet/glutinous rice = 25g flour/cornmeal/oatmeal = 35g steamed bun = 200g fresh corn (with core)
160ml milk = 20g milk powder = 25g cheese = 130ml unsweetened yogurt = 400ml soy milk
50g lean meat = 80g fish/shrimp = 50g tofu strips/dry tofu = 100g northern tofu = 150g southern tofu = 1 egg (approx. 60g)
200g pear/peach/apple/mandarin orange/orange/kiwi/grapes = 150g banana/fresh lychee = 500g watermelon = 300g strawberries
500g leafy/stem vegetables = 400g white radish/bell pepper/bamboo shoots = 350g

pumpkin/cauliflower = 250g fresh long beans/garlic shoots/green beans/onions = 200g carrots = 150g yam/water chestnut/lotus root/potatoes
15g peanuts = 15g walnuts = 10ml peanut oil

3）Follow-up

Nutritional education will be provided, and adherence to the nutritional prescription will be closely monitored. Patients will record their daily dietary intake and the extent to which they follow the nutritional prescription. Adjustments to the nutritional prescription will be made at 3 and 6 months.

4.ACUPOINT MASSAGE

Evaluate subjects for constipation and insomnia, and teach relevant acupoint massage therapy methods.Based on the assessment results, provide recommendations for acupoint massage therapy. Use the thumb pad to gently massage the acupoints, applying moderate pressure until a slight sensation of soreness and distension is felt. Each session should last 5-10 minutes, performed 1-2 times daily.

1）Constipation

Zhigou (TE6) 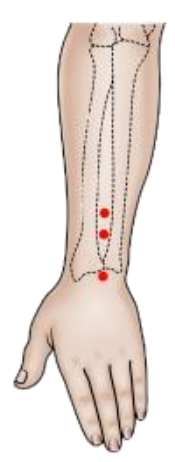	Located on the dorsal side of the forearm, 3 cun above the dorsal wrist crease, and is part of the Hand Shaoyang Sanjiao meridian.

Zusanli (ST36)<br>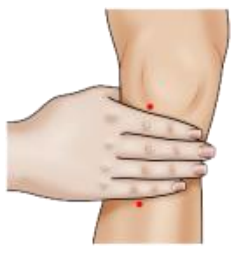	Located on the lateral side of the lower leg, 3 cun below Dubi (ST35), on the line connecting Dubi and Jiexi (ST41), on the Foot Yangming Stomach meridian.
Dachangshu (BL25)<br>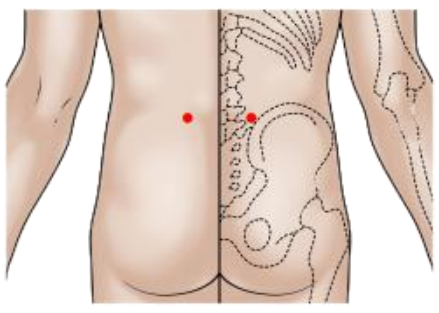	Located 1.5 cun lateral to the lower border of the spinous process of the 4th lumbar vertebra, on the Foot Taiyang Bladder meridian.

2）Insomnia

Neiguan (PC6)<br>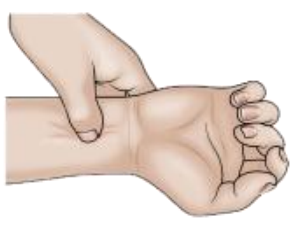	Located on the palmar side of the forearm, 2 cun above the transverse wrist crease, between the tendons of the palmaris longus and flexor carpi radialis, on the Hand Jueyin Pericardium meridian.
Shenmen (HT7)<br>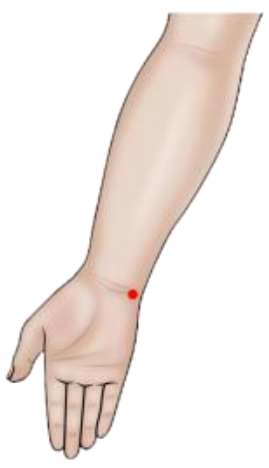	Located on the wrist, at the ulnar end of the transverse crease of the wrist, in the depression on the radial side of the tendon of the flexor carpi ulnaris, on the Hand Shaoyin Heart meridian.
Sanyinjiao (SP6)	Located on the medial side of the lower leg, 3 cun above the tip of the medial malleolus, posterior to

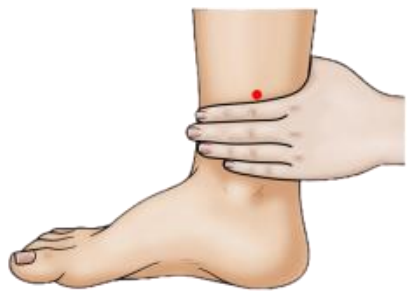	the medial border of the tibia, on the Foot Taiyin Spleen meridian.

3）Follow-up

Provide TCM education and monitor adherence to acupoint massage. Patients should record the location and duration of each acupoint massage session daily.Adjust the treatment plan based on assessments conducted at 3 and 6 months after enrollment.

5.POLYPHARMACY MANAGEMENT

Clinical pharmacists will evaluate the appropriateness, efficacy, safety, and adherence of medications used by frail patients. Any drug-related issues identified will be discussed with the physician and the patient. If necessary, adjustments to the medication regimen will be made, or a referral to another department will be recommended.

1）Process

①Information Collection and Verification. Gather and verify information regarding the patient's current and past medical history. Cross-check previous medication usage and perform a medication reconciliation.

②Analysis and Evaluation. Assess the medication regimen based on the patient's individualised treatment goals, focusing on appropriateness, efficacy, safety, and adherence. Identify any drug-related problems.

③Plan Development. Collaborate with the physician to formulate an initial medication treatment plan.

④Plan Implementation. Work in cooperation with healthcare staff to carry out the intervention.

2）Follow-up

Document the patient's medication use at the 3-month and 6-month follow-up points. Evaluate the appropriateness, efficacy, safety, and adherence of the medications. Any drug-related issues identified will be discussed with the physician and the patient. Adjustments to the medication regimen will be made if necessary.

Appendix 2. Manual to Record Daily Exercise, Meal Intake, and Acupoint Massage Activities

Holistic Health Tracker

(Daily Exercise, Nutrition, and Acupoint Massage)

Record your life

Improve your health

Rehabilitation

1. Basic Exercises:

    A. Posture Correction

    B. Breathing Adjustment

    C. Abdominal Tension Exercise

    D. Pelvic Floor Muscle Exercise

2. Numbered Exercises: (1-8)

3. Aerobic Exercise

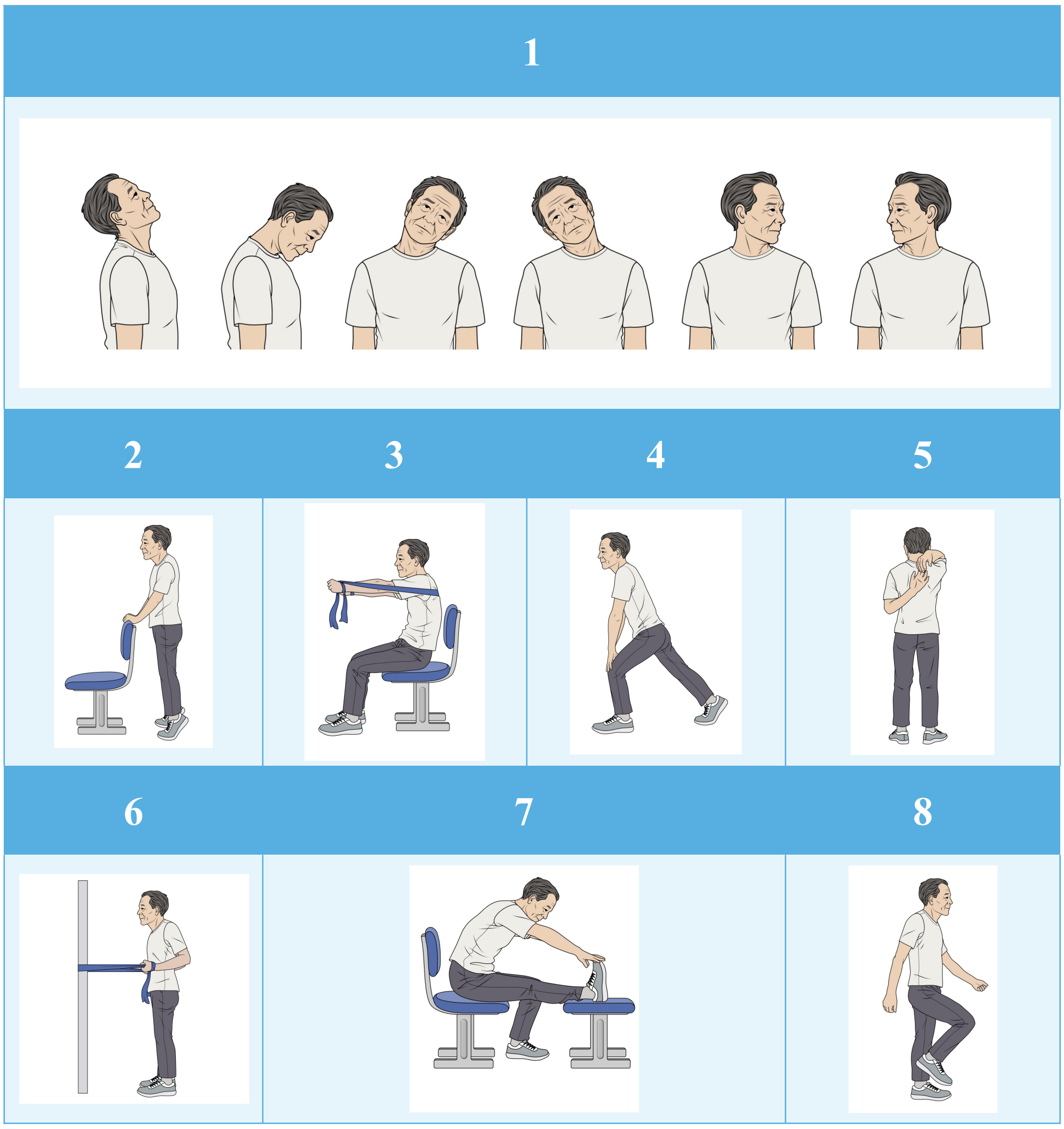

Nutrition

Recommended Daily Food Content and Quantity	
**Eggs(pcs)**	
**Milk(ml)**	
**Lean Meat(g)**	
**Grains(g)**<br><br>**The wheat starch-based staple foods(g)**	
**Vegetables(g)**	
**Fruits(g)**	
**Cooking Oil(g)**	
**Supplements(g)**	

Record

Acupoint massage

Constipation	Zhigou (TE6)	Zusanli (ST36)	Dachangshu (BL25)
	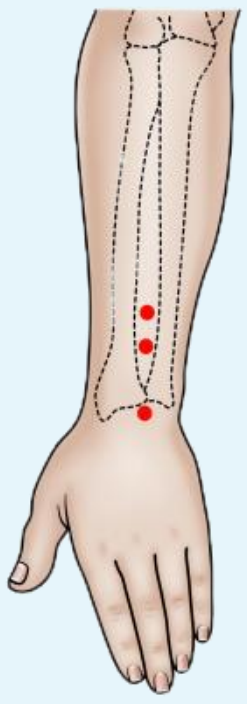	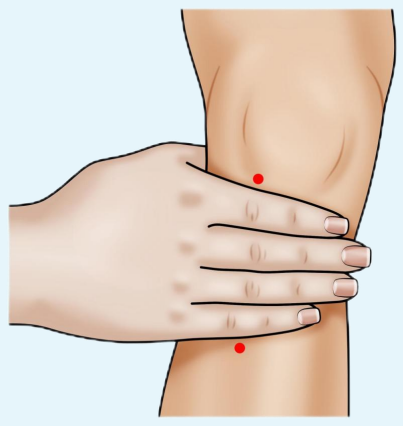	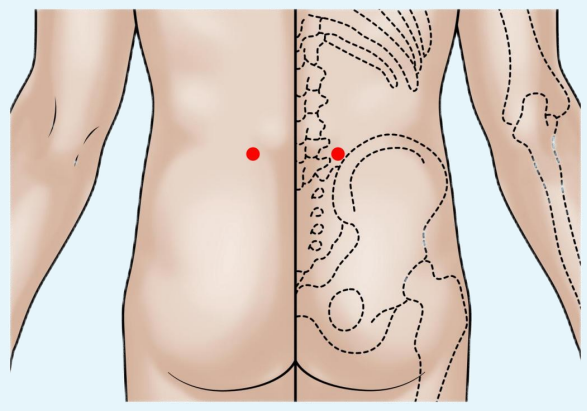

Insomnia	Neiguan(PC6)	Shenmen(HT7)	Sanyinjiao(SP6)
	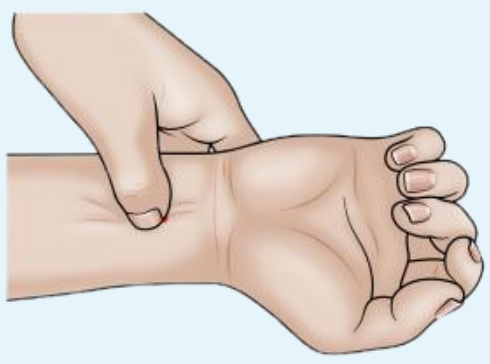	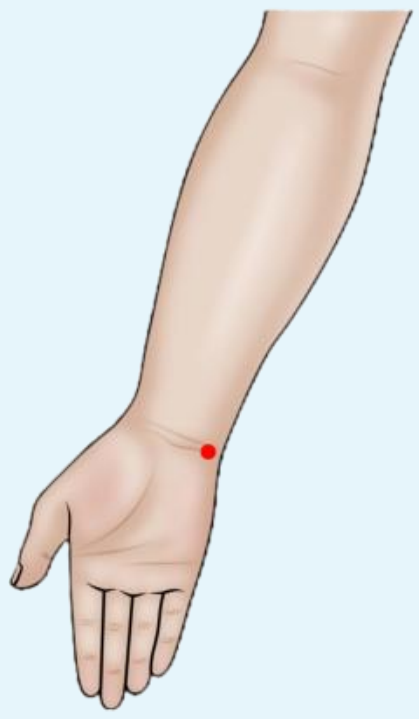	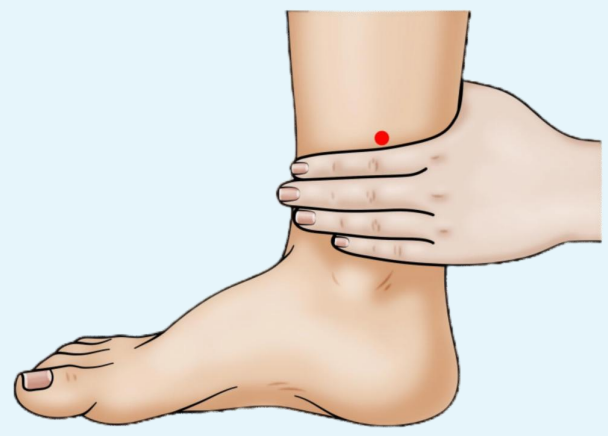

Health Improvement Guidelines:

- Follow the steps and complete each task daily.
- Keep track of your progress every day.
- Share your feedback regularly, including any challenges or changes you notice.
- Stay committed to improving your health by sticking with these activities.

Frailty Intervention Health Record

Item		Monday	Tuesday
Rehabilitation	Basic Movements		
	Numbered Movements		
	Exercise Time		
	Your Feeling		
Nutrition	Meat, Eggs, and Dairy Completion Rate	50% 100%	50% 100%
	Grain Snacks Completion Rate	50% 100%	50% 100%
	Vegetables and Fruits Completion Rate	50% 100%	50% 100%
	Cooking Oils and Nuts Completion Rate	50% 100%	50% 100%
	Nutritional Supplements Completion Rate		
Traditional Chinese Medicine	Massage Time		
	Your Feeling		

Wednesday	Thursday	Friday	Saturday	Sunday
50% 100%	50% 100%	50% 100%	50% 100%	50% 100%
50% 100%	50% 100%	50% 100%	50% 100%	50% 100%
50% 100%	50% 100%	50% 100%	50% 100%	50% 100%
50% 100%	50% 100%	50% 100%	50% 100%	50% 100%

Appendix 3. Clinical Research Protocol

Note: The final statistical analysis adhered to CONSORT guidelines; baseline characteristics are presented descriptively without formal hypothesis testing. See main manuscript Methods for details.

Study Title: The effect of comprehensive assessment and multi-disciplinary management for the geriatric and frail patients: A multi-centre, randomised, parallel controlled trial (MDT-F)

Funding: Beijing Municipal Science and Technology Commission, China (No. D181100000218003)

Lead Institution: Beijing Hospital

Principal Investigator : Hua Wang

Mobile: 86-13911680467

Email: wh74220@aliyun.com

Version Number: 1.0

Version Date: March 10, 2019

Background

Frailty is defined as the deterioration of multiple physiological systems and an increased susceptibility to stressors. It is a core component of geriatric syndromes and a precursor to disability, severely impacting the functional capacity and quality of life of elderly patients and posing a significant threat to healthy life expectancy. The prevalence of frailty increases with age, ranging from 4.0% to 59.1% among elderly adults in community-based researches, and is higher in hospitalised elderly (50%–80%). Related data are limited in China, ranging from 4.9% to 83.4%.[1-4]

Compared to non-frail elderly, frail older adults face a 15%–50% higher average mortality risk. Frail individuals are more prone to adverse clinical events and consume substantial healthcare resources. Studies indicate that frailty is strongly associated with poor prognosis and is an independent predictor of falls, mobility decline, functional deterioration, hospitalisation, and death. With aging population becoming increasingly prominent in China, particularly in Beijing, the impact of frailty on families and socioeconomic burdens is immeasurable. Early identification and effective intervention could mitigate or reverse frailty severity, preventing adverse health outcomes and yielding significant benefits for frail elderly, their families, and the whole society.

Hospitalisation exacerbates functional decline in elderly patients, many of whom fail to fully recover post-discharge, increasing risks of frailty, disability, dependency, rehospitalisation, and death.[5] Additionally, patients experience a period of heightened vulnerability post-discharge, during which the risk of adverse events may increase.[6]

In summary, frail elderly inpatients face high risks of functional decline and adverse outcomes during and after hospitalisation. Thus, urgent interventions to improve frailty status are necessary.

Current researches on frailty interventions are limited. Cameron et al. Found that

multidisciplinary interventions improved frailty in a randomised controlled trial (RCT) in 2013.[7] Ng et al. found that rehabilitation, nutrition, and cognitive interventions improved frailty in community-dwelling elderly in another RCT in 2015.[8] Losa-Reyna et al. found that strength- and gait-based exercise training improved frailty over six weeks in a non-RCT in 2019.[9] While studies have not explored frailty reversal, preliminary work on comprehensive geriatric assessment (CGA) and multidisciplinary interventions has shown improvements in mobility, falls, rehospitalisation rates, anxiety/depression, and quality of life.[10-12] Thus, multidisciplinary interventions may be a promising approach to improve frailty among elderly inpatients.

This study aims to investigate the prognostic impact of multidisciplinary interventions through a randomised controlled, single-blind, parallel-group, multicentre clinical trial using Fried Frailty Phenotype (FFP) score for frailty screening. If successful, this model could enhance care for frail elderly, optimize public healthcare resources, and provide evidence for China's elderly health service system.

Objectives

This prospective, randomised controlled, single-blind, parallel-group, multicentre clinical study compares comprehensive assessment and multidisciplinary interventions with traditional management in frail elderly patients. Outcomes include FFP score, Short Physical Performance Battery (SPPB), Clinical Frailty Scale (CFS), unplanned visits/rehospitalisation rates, disability (BADL), quality of life (EQ-5D), healthcare costs, mortality, falls, and nutritional assessment (NRS2002).

Study Design

1. Sample Size

The sample size was calculated using the formula for comparing means between two groups, with a power of 80%, a two-sided type I error of 0.05, and an estimated dropout rate of 20%.

Ian D Cameron et al. studied frail patients using the reduction in FFP score from baseline after multidisciplinary intervention as the primary endpoint. Their results showed:

- Intervention group: mean=0.8, SD=1.19
- Control group: mean=0.41, SD=1.02

- When using SPPB score as the primary endpoint, their results were:
    - Intervention group: mean=5.83, SD=2.82
    - Control group: mean=4.69, SD=2.91

Tze Pin Ng et al. studied pre-frail and frail patients with post-intervention Fried score as the primary endpoint, reporting:

- Intervention group: mean=1.2, SD=1.07
- Control group: mean=1.6, SD=0.97

For the current study focusing on frail patients with both FFP and SPPB scores after multidisciplinary intervention as co-primary endpoints, a minimum of 160 matched pairs (total sample size=320 subjects) is required to achieve adequate statistical power.

2.Study Population

Frail elderly inpatients from department of cardiology in Beijing Hospital, PLA General Hospital, and Beijing Tsinghua Changgung Hospital.

2.1 Inclusion Criteria

(1) age ≥ 70 years; (2) FFP score ≥3; (3) long-term residency in Beijing; (4) voluntarily participate in the study and sign informed consent.

2.2 Exclusion Criteria

(1) currently participating in other similar clinical trials; (2) have been treated with regular (≥3 days per week) traditional Chinese medicine (TCM) or acupuncture or have received regular (≥3 days per week) nutritional supplements or received treatment at a rehabilitation facility within the last 3 months; (3) have exercised regularly at moderate intensity over the past 3 months [maximum heart rate of 50% (220–age) during exercise]; (4) are unable to complete the SPPB (main result of the

study); (5) are unable to participate in and stick to exercise alone; (6) will be residing in a hospital or care home, including community specialist nursing facilities, rehabilitation centres and intermediate care facilities in the coming year; (7) have dementia or severe cognitive impairment; (8) have other severe concomitant conditions impair the ability of the patient; (9) have end-stage disease with a life expectancy <12 months; (10) have any other conditions or events that researchers or therapists consider to be excluded.

2.3 Termination and withdrawal criteria

In the course of the study, if the researchers consider that continuing to participate in the study will lead to adverse outcomes, then the subjects participation will be terminated and they will be asked to withdraw from the study. The subjects are also free to request withdrawal at any time.

3.Methods and Procedures

3.1 Outline

In this study, 320 elderly participants ≥70 years old will be randomly divided into the intervention group and control group. The intervention group will be given a CGA and multi-disciplinary management for 12 months besides a routine diagnosis and treatment, and the control group will be followed up for 12 months with routine management for basic diseases.

3.2 Outcomes

The primary outcomes: (1) Fried Frailty Phenotype; (2) the short physical performance battery (SPPB).

The secondary outcomes: (1) the clinical frailty scale (CFS); (2) non-elective hospital readmission, which is considered any type of emergency readmission; (3) disability, which is assessed by basic activities of daily living (BADL); (4) health-related quality of life, which is evaluated by the 5–level European Quality of Life 5 Dimensions index (EQ-5D); (5) medical insurance expenses; (6) fall events; (7) nutrition risk screening 2002 (NRS-2002); (8) all-cause mortality.

3.3 Recruitment process and follow-up

A flowchart of the trial is shown in Figure 1.

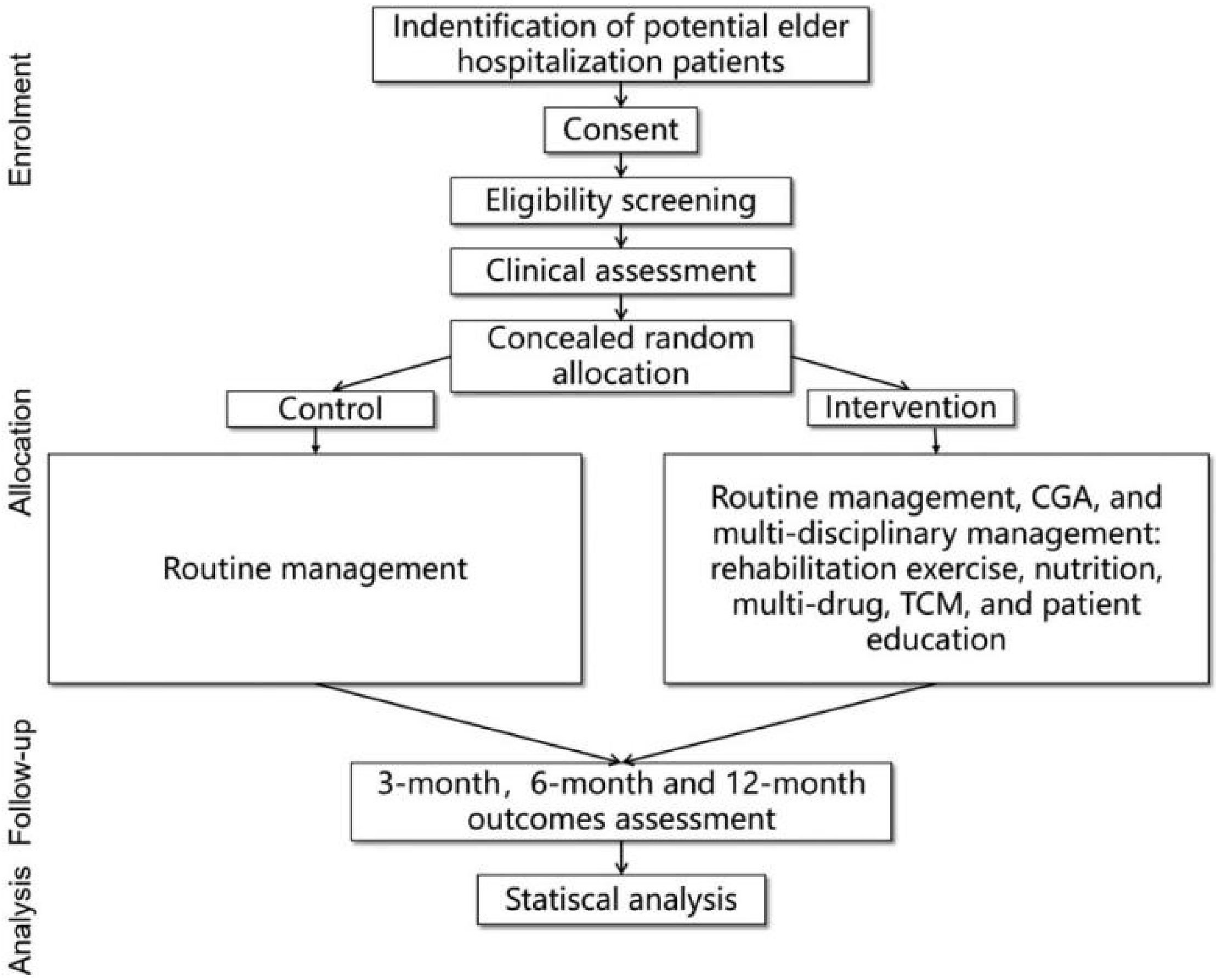

Figure 1. Overview of the participant flow for assessing the effect of a CGA and multi-disciplinary management on geriatric frail patients in China

CGA=comprehensive geriatric assessment; TCM = traditional Chinese medicine.

3.3.1 Recruitment

The researchers will recruit participants who meet the inclusion criteria and do not meet the exclusion criteria from cardiac ward at Beijing Hospital, PLA General Hospital and Tsinghua Changgeng Hospital. The researchers will explain the research plan and requirements in detail to the patients, obtain their consent and sign the informed consent form. The researchers will collect and evaluate the clinical data of the participants, including demographic data, medical history, physical examination, and related examinations.

3.3.2 Randomisation and blinding

A total of 320 participants will be randomly assigned by a central computer

randomisation software programme, including 160 participants in the intervention group and 160 participants in the control group. The randomisation sequence is automatically generated when the researchers complete the clinical evaluation and input the electronic data capture system (EDC), and artificial intervention is impossible.

This study is a single-blind trial design. Considering that the study needs the cooperation of participants and multi-disciplinary intervention teams, it is not possible to blind participants and staff administering interventions to group allocation. However, the staff performing the clinical evaluations, outcome assessors and data analysts will be blinded to the intervention group assignment. Assessors will not be involved in intervention activities.

This study will strictly select participants in accordance with the inclusion and exclusion criteria. It adopts a randomised, multicentre clinical research method. Through the telephone and follow-up platform, a good trust relationship will be established with patients to reduce choice deviation and increase compliance. The endpoint events of this study are all objective indicators. Researchers will quantify these objective indicators and establish unified and clear criteria. The assessors will receive unified and standardised training before investigation. Through brief telephone reviews and record books, a good trust relationship with participants will help reduce selection bias and increase compliance.

3.3.3 Intervention

Participants in the intervention group will receive a CGA and receive 12 months multi-disciplinary management. Interventions will be carried out by a multi-disciplinary team (including physicians, nurses, nutritionists, rehabilitation physicians, phar macists, and TCM physicians). A CGA will be carried out by nurses during hospitalisation, including nutritional status assessment, activities of daily living assessment and insomnia assessment.

The interventions are as follows:

(1) Nutrition aspect Assessment: Age, grip strength, daily physical activity, NRS 2002 score, clinical diagnosis (especially sarcopenia), laboratory results, human body

composition analysis (Inbody 720), dietary survey.

Advice: A reasonable dietary plan will be formulated by the nutritionist.

(2) Rehabilitation Assessment: SPPB, physical fitness tests, including a 30 second sit-to-stand test to assess lower body strength, a 30-second arm curl test to assess upper body strength, a 2 minutes step test to assess the cardiopulmonary function and aerobic endurance, a chair sit-and-reach test to assess lower body flexibility, a back scratch test to assess upper body flexibility, and a time-up-and-go test (TUG) to evaluate dynamic balance and agility.

Advice: A set of rehabilitation exercises will be taught, including breath adjustment, posture adjustment, guidance for contracting abdominal muscles and pelvic floor muscles, head and neck stretching, back scratching, sitting forward stretch ing, sitting elastic belt assisted upper limb movement, heel lifting and stepping. Each participant will be given a sports manual with exercise instructions and safety information. During the period of hospitalisation, all training sessions will be carefully supervised by a rehabilitation physician experienced in therapeutic training. Participants will perform 3 to 20 repetitions for each exercise and 3 to 5 times per week for 12 months, and accurately record the frequency, time and feelings concerning exercise each week, gradually increasing to the maximum tolerance and safety intensity. Participants will also be encouraged to exercise on their own and walk for a few minutes each day.

(3) Multi-drug evaluation Assessment: Medication appropriateness index (MAI), review of the type, measurement and quantity of drugs used by patients during hospitalisation.

Advice: Pharmacists will participate in the administration of drugs to the participants, potentially harmful drugs or doses will be revised and patients will receive instructions concerning medication when discharged.

(4) TCM Assessment: TCM physicians will comprehensively and systematically assess the participants based on the TCM theory of syndrome differentiation, including a TCM tongue diagnosis and pulse diagnosis.

Advice: TCM physicians will deliver guidance on performing acupoint massage related to constipation and insomnia, including Zhigou, Zusanli, Dachangshu,

Neiguan, Shenmen, and Sanyinchiao. Each participant will be instructed on 0 to 4 related acupoints, and each acupoint will be massaged for 5 minutes every day.

(5) Patient education: Nurses will issue a patient education manual to each participant, covering the basic introduction of frailty, fall prevention education, first-aid knowledge and cardiovascular medicine guidance. All participants in both groups will undergo routine management.

Participants in the control group will be provided with routine inpatient diagnosis and treatment based on their existing diseases, including disease treatment and nursing intervention. The nursing intervention involved conducting a routine assessment upon the patients' admission and implementing corresponding nursing measures based on the assessment results.

3.3.4 Follow-up

All enrolled patients will be followed up at outpatient clinics at 3, 6, and 12 months after enrollment to evaluate endpoint events, with data recorded in an electronic data management system.

Intervention Group: A digital health record and follow-up system were established. The health record includes basic information, diagnosis, medical history, medications, adverse events, follow-up notifications, and regular patient education. Twelve sessions of patient education will be conducted. Brief telephone reviews will be conducted for all participants to promote adherence to the intervention.

Nutrition: At 3, 6, and 12 months, dietitians will assess dietary intake, calculating total daily energy, macronutrient intake, and proportions, while recording consumption of specific foods (e.g., fish oil, vitamins D/E/C, protein powder, complete nutrients). Dietitians will adjust prescriptions to optimize dietary structure. Nutritional prescriptions will be promptly revised if clinical conditions changed. Monthly phone reminders will correct unsuitable dietary habits, and personalised goal-setting assisted meal planning.

Rehabilitation Exercise: Monthly phone follow-ups (or clinic visits if possible) will identify clinical needs to modify or terminate exercise regimens, ensuring intensity increases only if tolerated without adverse events. Exercise compliance will

be reinforced during 3- and 6-month clinic evaluations.

Polypharmacy: Pharmacists will monitor medication adjustments and adverse effects throughout follow-up, with quarterly phone check-ins.

TCM: In addition to scheduled 3-, 6-, and 12-month visits, patients could book appointments for TCM regimen adjustments as needed.

Patient Education: Regular clinic or phone-based fall prevention and emergency care education will be supplemented. In-person reinforcement occurs at 3, 6, and 12 months.

Compliance: Participants will be required to make records in the debilitating record book. Weekly exercise frequency/duration, clinic visits, and satisfaction surveys will be recorded. Participants in the intervention group will be defined as treatment-compliant if they complete at least 50% of the planned rehabilitation exercises and/or 50% of their planned nutrition guidance programme and 50% of the acupoint massage prescribed by TCM within 12 months.

Control Group: A phone follow-up will be conducted at 1 month post-discharge, with endpoint events evaluated at outpatient visits at 3, 6, and 12 months post-enrollment.

3.3.5 Statistical analyses

Statistical significance will be set at a 2-sided *P* value <0.05.

The participants clinical features will be compared between the intervention and control groups. Continuous variables will be expressed as the mean±standard deviation in a normal distribution and median and interquartile range in a non-normal distribution, and categorical variables will be expressed as numbers and percentages. Baseline characteristics will be compared by χ^2 tests or Fishers exact tests for categorical variables and t-tests or Wilcoxons rank sum tests for continuous variables.

We will summarize the number of enrollments and completion of each centre, list the drop-outs, compare the total drop-out rate and list the reasons for termination in detail and compare the characteristics between those with missing outcome data and those who have completed follow-up.

We will conduct an intention-to-treat (ITT) analysis for the primary analysis,

including primary and secondary outcomes, with a per-protocol (PP) analysis used for the sensitivity analysis. The ITT population is defined as all randomised participants in the study, and the PP population is defined as all randomised participants who were actually compliant with their treatment. A covariance analysis will be used to analyze the Fried Frailty Phenotype, SPPB and modified means of the 2 groups, taking the central effect of the test into account, and the 95% confidence interval (CI) of the difference between the 2 groups will be calculated. Only when the above 2 main indicators satisfy the statistical hypothesis (superior effect), can the hypothesis be considered valid. For time-event data, the Kaplan–Meier method will be used to estimate death, non-elective hospital readmission and fall events. A log rank test will be performed for the 2 groups. A Cox proportional hazards regression model will be used to calculate the effect of potential risk factors on the events.

The safety evaluation table will be included to describe the number of adverse events and the incidence of adverse events, serious adverse events, adverse reactions and serious adverse reactions in the two groups.

4. Flowchart

Table 1 Schedule of enrollment, interventions, and assessments

	Visit 1	Visit 2	Visit 3	Visit 4
Time (months±days)	0±5	3±14	6±30	12±30
Consent	✔			
Randomisation	✔			
**Clinical Evaluation**				
Demographic data	✔			
Medical history	✔			
Physical examination	✔	✔	✔	✔
**Related auxiliary examination**, including routine blood test (±7), blood biochemical (±7), D-dimer (±7), NT-pro-BNP (±7), 25-OH-VD_3 (±7), hsCRP (±7), thyroid function (±30), and echocardiogram (±30).	✔		✔	✔
**CGA***	✔			
**Specimen collection**				
Blood	✔		✔	✔
Urine	✔	✔	✔	✔

feces	✔		✔	✔
**Primary Outcomes**		✔	✔	✔
**Secondary Outcomes**		✔	✔	✔
**Adverse Events**	✔	✔	✔	✔

* Intervention group only.

25-OH-VD$_3$ = 25-dihydroxy-vitamin D$_3$, CGA = comprehensive geriatric assessment, hsCRP = high-sensitivity C-reactive protein, NT-pro-BNP = N-terminus pro-B-type natriuretic peptide.

5. The handling plan for adverse events

This study is a randomised controlled clinical trial. The main intervention measures involve dietary guidance, exercise guidance, medication guidance and patient education. Its serious adverse events/adverse events are mainly related to exercise guidance. The serious adverse events/adverse events are defined as follows:

Adverse events: fractures associated with rehabilitation exercise.

Severe adverse events: persistent or severe disability or dysfunction caused by rehabilitation exercise.

If adverse or severe adverse events occur, the relationship between them and the intervention will be assessed, the intensity and mode of intervention will be adjusted, the intervention will be suspended if necessary, and the participants will receive appropriate treatment. We will immediately provide diagnosis and treatment, and report the occurrence in a timely manner while compensating the participant according to the relevant laws and regulations of our country.

6. Data Management Plan

Patients will be strictly enrolled in accordance with the unified diagnostic criteria. After enrollment, medical files will be established for the patients, and a dedicated person will be responsible for the management of the patients' medical files, including data entry, backup, update and organization. A complete electronic data management system for standardised management will be established based on the case report form. All the data of the patients can be traced, and the traceability of the data needs to be

evaluated during the verification. Establish a quality control centre with dedicated personnel responsible for coordinating the entire research work, regularly announce the research progress, and solve the problems existing in the research. The independent data and Monitoring committee regularly monitors and analyzes the data.

7. Expected research results

By comparing the comprehensive multidisciplinary intervention for elderly frail patients with the simple conventional traditional treatment methods, this study will explore the reasonable treatment methods suitable for frail elderly patients, in order to reduce the occurrence of adverse prognosis of frailty and reduce the expenditure on health economics. It is expected to publish 3 to 5 papers and train 2 to 4 postgraduate students.

8. Ethical issues

This research will strictly abide by *the Helsinki Declaration* and relevant laws and regulations on ethical review. Before the start of the research, the relevant materials must be submitted to the ethics committee for review. Only after the review is approved can the clinical research be carried out. The subjects should sign the informed consent form before participating in the study. During the research process, any modifications to the research plan, informed consent form and other documents need to be resubmitted to the ethics committee for review. Only after approval can they be implemented. Submit research progress reports regularly. When the research information and data obtained from this study are published at scientific conferences or in scientific journals, the identities of the subjects will not be made public.

Appendix 4. Statistical Analysis Plan (SAP)

1.Sample Size

The sample size for this study was determined based on the mean comparison between two groups using the difference test formula (Power = 80%, *P* = 0.05). Previous studies suggested the following sample sizes: 250 participants (125 per group) to detect a 33% increase in the mean number of frailty criteria (from 1.2 to 1.6), 256 participants (128 per group) to detect a 41% between-group difference in the average reduction in the number of frailty criteria (from 0.41 to 0.8), and 202 participants (101 per group) to detect a 1.44-point between-group difference in the average increment on the 12-point SPPB scale (from -0.98 to 0.52). Considering an attrition rate of 20%, a minimum of 320 participants (160 per group) was recruited.

2.Analysis sets

The study included two analysis sets: the Intention-to-Treat (ITT) analysis set and the Per-Protocol (PPS) analysis set.

- The Intention-to-Treat set (ITT) : The ITT set comprises all eligible participants who were randomised and received treatment.
- Per-Protocol set (PPS): The PPS is a subset of the ITT, including only those participants who were randomised, received treatment, demonstrated good compliance, and had no missing primary outcomes.

Demographic and baseline analyses will be conducted based on the ITT, while efficacy analysis will be primarily based on the ITT. Sensitivity analyses of efficacy outcomes will be performed using the PPS.

3.General statistical considerations

Statisticians are responsible for developing a statistical analysis plan (SAP) with the principal investigator after the protocol is confirmed. Statistical analysis was done by SAS 9.4 (SAS Institute, Cary, NC). A 2-tailed P<0.05 is considered statistically significant in all statistical tests. (If no other specification)

The number of subjects, mean, median, standard deviation, the first quartile (Q1), the third quartile (Q3), minimum, and maximum are included in the summary statistics of continuous variables. The number of subjects, frequency and percentages are included in summary statistics of the categorical variables.

Continuous variables are summarized with mean and SD and compared using paired t-test or Wilcoxon rank test, as appropriate. Categorical variables are summarized with frequencies and percentages and compared using chi-square test or Fisher exact test, as appropriate. Time-to-event analyses will be conducted using the Kaplan-Meier estimate and cox regression model.

4.Statistical method

4.1 Enrollment, Baseline and Demographic analysis

Summarize the enrollment and completion numbers for each centre. Present the different datasets sizes across groups, and compare the overall dropout rates. Describe the demographic characteristics of patients (such as age, height, and vital signs), medical history, and medication history. Additionally, compare age, height, and weight for each group to assess the comparability. Baseline characteristics will be summarized and compared between groups followed generally statistical considerations, using chi-square tests or Fisher's exact tests for categorical variables, and t-tests or Wilcoxon rank sum tests for continuous variables.

The baseline and demographic analysis will be conducted on the ITT.

4.2 Efficacy analysis

4.2.1 Primary efficacy analysis:

(1) the Fried Frailty Phenotype score change from baseline to 12 months, with superiority hypothesis, the hypothesis is as following:

$H0\colon\ \pi_T - \pi_C \le 0$

$H1\colon\ \pi_T - \pi_C > 0$

$\alpha = 0.025$ (one-sided test)

At a significance level of α=0.025 (one-sided test) and a power of β=0.2, calculate the 12-month change in Fried scores for both groups and the 95% confidence interval for the difference in these changes. If the lower limit of the 95% confidence interval for the difference in Fried score changes between the two groups is greater than 0, it can be concluded that the experimental group is superior to the control group.

(2) SBBP change from baseline to 12 months, with superiority hypothesis, the hypothesis is as following:

$H0\colon\ \pi_T - \pi_C \le 0$

$H1\colon\ \pi_T - \pi_C > 0$

$\alpha = 0.025$ (one-sided test)

At a significance level of α=0.025 (one-sided test) and a power of β=0.2, calculate the 12-month change in SBBP scores for both groups and the 95% confidence interval for the difference in these changes. If the lower limit of the 95% confidence interval for the difference in Fried score changes between the two groups is greater than 0, it can be concluded that the experimental group is superior to the control group.

Primary efficacy endpoints will be analysed as repeated measures (longitudinal) data, using both analysis of variance (ANOVA) and mixed-effects models.

Repeated measures analysis of variance (ANOVA) will be used to compare between-group difference at each visit. Covariance analysis will be applied to evaluate the Fried Frailty Phenotype (FFP) and SPPB scores, adjusting for central effects, with adjusted means and 95% CIs calculated for differences between the two groups.

Mixed effect model will also be applied for estimating least squares means (Lsmeans) and corresponding 95%CI for FFP change and SPPB change. Individual was considered as random effect.

4.2.2 Secondary efficacy analysis:

Statistically analysis followed by the general statistical consideration. For longitudinal endpoints, statistical summary and between-group comparison were done by ANOVA and Mixed-effect models.

For time-to-event endpoints, Kaplan–Meier methods estimated survival functions for death and hospital readmission, with log-rank tests comparing the two groups. Cox proportional hazards regression models were used to evaluate the hazard ratio between groups on these events.

4.3 Subgroup analysis

For the primary efficacy endpoints, subgroup analysis will be conducted on both the ITT and PPS populations. Between-group differences within each subgroup will be compared, and the results will be presented using forest plots. The subgroups are as follows:

① Age (<80y, ≥80y)

② Sex (Male, Female)

③ BMI (18≤BMI≤24, BMI>24)

④ If Coronary Artery Disease(CAD) (Yes, No)

⑤ If Atrial Fibrillation/Flutter (AFL/AF) (Yes, No)

⑥ If Diabetes (Yes, No)

⑦ If Heart Failure (Yes, No)

For time-to event endpoints, Kaplan-Meier methods will be used to estimate survival functions for death and hospital readmission, with log-rank tests comparing the two groups. Cox proportional hazards regression models will also be used to evaluate the hazard ratio between groups on these events. Additionally, a multivariable Cox regression model will be performed, incorporating subgroup variables as covariates to adjust for potential confounding effects within the subgroups.

5. Missing value imputation

In the ITT analysis, missing values for the primary outcomes will be imputed using the EM method, with a single imputation performed and a fixed seed to ensure reproducibility. In the PPS analysis, no imputation will be conducted for missing data. For time-to-event endpoints, events occurring beyond 1 year will be censored, with the time imputed as 365 days.

References

1. Collard, R. M., Boter, H., Schoevers, R. A. & Oude Voshaar, R. C. Prevalence of frailty in community-dwelling older persons: a systematic review. *J. Am. Geriatr. Soc.* **60**, 1487-1492 (2012). doi: 10.1111/j.1532-5415.2012.04054.x.

2. Dent, E., Chapman, I., Howell, S., Piantadosi, C. & Visvanathan, R. Frailty and functional decline indices predict poor outcomes in hospitalised older people. *Age Ageing*. **43**, 477-484 (2014). doi: 10.1093/ageing/aft181.

3. Makary, M. A., Segev, D. L., Pronovost, P. J. et al. Frailty as a predictor of surgical outcomes in older patients. *J. Am. Coll. Surg.* **210**, 901-908 (2010). doi: 10.1016/j.jamcollsurg.2010.01.028.

4. Chinese Society of Geriatrics. [Chinese expert consensus on assessment and intervention for elderly patients with frailty]. *Zhonghua Lao Nian Yi Xue Za Zhi*. **36**, 251-256 (2017). (in Chinese). doi:10.3760/cma.j.issn.0254-9026.2017.03.007.

5. Deer, R. R., Goodlett, S. M., Fisher, S. R. et al. A Randomized Controlled Pilot Trial of Interventions to Improve Functional Recovery After Hospitalization in Older Adults: Feasibility and Adherence. *J. Gerontol. A Biol. Sci. Med. Sci.* **73**, 187-193 (2018). doi: 10.1093/gerona/glx111.

6. Deer, R. R., Dickinson, J. M., Fisher, S. R. et al. Identifying effective and feasible interventions to accelerate functional recovery from hospitalization in older adults: A randomized controlled pilot trial. *Contemp. Clin. Trials* **49**, 6-14 (2016). doi: 10.1016/j.cct.2016.05.001.

7. Cameron, I. D., Fairhall, N., Langron, C. et al. A multifactorial interdisciplinary intervention reduces frailty in older people: randomized trial. *BMC Geriatr.* **11**, 65 (2013). doi: 10.1186/1741-7015-11-65.

8. Ng, T. P., Feng, L., Nyunt, M. S. Z. et al. Nutritional, Physical, Cognitive, and Combination Interventions and Frailty Reversal Among Older Adults: A Randomized Controlled Trial. *Am. J. Med.* **128**, 1225-1236 (2015). doi: 10.1016/j.amjmed.2015.06.017.

9. Losa-Reyna, J., Baltasar-Fernandez, I., Alcazar, J. et al. Effect of a short multicomponent exercise intervention focused on muscle power in frail and pre frail

elderly: A pilot trial. *Exp. Gerontol.* **115,** 114-121 (2019). doi: 10.1016/j.exger.2018.11.022.

10. Huang, Z. G. & Li, Y. [Effect of individualized treatment on prognosis of elderly hospitalized patients with chronic heart failure]. *Hebei Yi Xue* **24**, 202-205 (2018). (in Chinese). doi:10.3969/j.issn.1006-6233.2018.02.006.

11. Deng, B. F., Luo, C. C., Li, H. F. et al. [Effect of comprehensive geriatric assessment on activities of daily living and nursing adverse events in elderly hospitalized patients]. *Zhonghua Xian Dai Hu Li Za Zhi* **20**, 937-939 (2014). (in Chinese). doi :10.3760/cma.j.issn.1674-2907.2014.08.023.

12. Wu, J., Yang, X. & Zhou, Y. [Effects of multidimensional collaborative nursing on negative emotions and quality of life in elderly patients with coronary heart disease and heart failure]. *Zhonghua Xian Dai Hu Li Za Zhi* **24**, 577-581 (2018). (in Chinese). doi:10.3760/cma.j.issn.1674-2907.2018.05.020.
