# Supplementary material for: Multidisciplinary intervention in frail elderly patients with cardiovascular diseases: a multicentre, single-blinded, randomised controlled trial
Source: Nat Commun. 2026 Aug 31;17:10340. doi: 10.1038/s41467-026-77277-7 (PMC13623951; doi:10.1038/s41467-026-77277-7)
Supplement: Supplementary file 2 [file 41467_2026_77277_MOESM2_ESM.pdf]

nature portfolio

Corresponding author(s): Hua Wang

Last updated by author(s): Aug 14, 2026

Reporting Summary

Nature Portfolio wishes to improve the reproducibility of the work that we publish. This form provides structure for consistency and transparency in reporting. For further information on Nature Portfolio policies, see our Editorial Policies and the Editorial Policy Checklist.

Statistics

For all statistical analyses, confirm that the following items are present in the figure legend, table legend, main text, or Methods section.

n/a	Confirmed	
☐	☒	The exact sample size (*n*) for each experimental group/condition, given as a discrete number and unit of measurement
☐	☒	A statement on whether measurements were taken from distinct samples or whether the same sample was measured repeatedly
☐	☒	The statistical test(s) used AND whether they are one- or two-sided<br>*Only common tests should be described solely by name; describe more complex techniques in the Methods section.*
☐	☒	A description of all covariates tested
☐	☒	A description of any assumptions or corrections, such as tests of normality and adjustment for multiple comparisons
☐	☒	A full description of the statistical parameters including central tendency (e.g. means) or other basic estimates (e.g. regression coefficient) AND variation (e.g. standard deviation) or associated estimates of uncertainty (e.g. confidence intervals)
☐	☒	For null hypothesis testing, the test statistic (e.g. *F*, *t*, *r*) with confidence intervals, effect sizes, degrees of freedom and *P* value noted<br>*Give P values as exact values whenever suitable.*
☒	☐	For Bayesian analysis, information on the choice of priors and Markov chain Monte Carlo settings
☒	☐	For hierarchical and complex designs, identification of the appropriate level for tests and full reporting of outcomes
☒	☐	Estimates of effect sizes (e.g. Cohen's *d*, Pearson's *r*), indicating how they were calculated

Our web collection on statistics for biologists contains articles on many of the points above.

Software and code

Policy information about availability of computer code

Data collection	Data were collected and entered into REDCap version 10.6.22 (Research Electronic Data Capture), an electronic data capture system established and managed by the Peking University Clinical Research Institute. No other software was used for data collection.
Data analysis	Statistical analyses were performed using SAS version 9.4 (Simplified Chinese edition; SAS Institute, Cary, NC, USA). Custom SAS code was written for the outcome analyses. The custom SAS programs used for data processing and statistical analyses have been deposited in Zenodo at https://doi.org/10.5281/zenodo.21026032.

For manuscripts utilizing custom algorithms or software that are central to the research but not yet described in published literature, software must be made available to editors and reviewers. We strongly encourage code deposition in a community repository (e.g. GitHub). See the Nature Portfolio guidelines for submitting code & software for further information.

Data

Policy information about availability of data

All manuscripts must include a data availability statement. This statement should provide the following information, where applicable:

- Accession codes, unique identifiers, or web links for publicly available datasets
- A description of any restrictions on data availability
- For clinical datasets or third party data, please ensure that the statement adheres to our policy

The raw participant-level clinical data from this randomised controlled trial cannot be publicly deposited because the institutional review board approval (Beijing

Hospital Ethics Committee, 2019BJYYEC-045-02) explicitly restricts data access to the study team to protect participant privacy, and the informed consent obtained from participants did not include provisions for open data sharing. Additionally, national health data regulations in China prohibit the unrestricted public release of individual-level clinical data. Aggregate and summary-level numerical source data underlying the figures and tables in the main manuscript and Supplementary Information are provided as a Source Data file with this paper. For analyses involving missing outcome data, the Source Data file reports aggregate model estimates from the ITT analyses; individual observed or imputed participant-level data are not publicly provided. Additional de-identified participant-level data not included in the Source Data file may be made available to qualified academic investigators for non-commercial scientific research. Data access requests should be submitted to the corresponding author (Hua Wang, email: wh74220@aliyun.com) and must include: (1) a brief research proposal stating the purpose and planned analyses; (2) a data confidentiality commitment form; and (3) a completed data use agreement specifying the intended use and duration of access. All requests will be reviewed by the study investigators and the institutional ethics committee. Approved requests will receive a response within 4-6 weeks, and data will be provided via a secure file transfer mechanism. Data will be available for a period of up to 2 years for the specified project only. Source data are provided with this paper.

Research involving human participants, their data, or biological material

Policy information about studies with human participants or human data. See also policy information about sex, gender (identity/presentation), and sexual orientation and race, ethnicity and racism.

Reporting on sex and gender	The term "sex" is used throughout the manuscript to describe the biological attribute of participants. The findings are presented for the entire study population, with no specific indications that the results apply to only one sex. Sex was reported as a baseline characteristic in Table 1. Of the 333 participants, 142 (42.6%) were male and 191 (57.4%) were female, with sex distribution balanced across intervention (44.0% male) and control (41.3% male) groups. Subgroup analyses were conducted for sex, but not exclusively; they were performed alongside other clinically relevant subgroups, with the primary focus being on clinical disease conditions. Individual-level sex data were collected based on self-reporting by the participants but public sharing is restricted per the data use agreement. Gender information was not collected.
Reporting on race, ethnicity, or other socially relevant groupings	The study did not collect data on race or ethnicity. Socially relevant variables included education level, occupational category, and residential status. Educational attainment was included as a key demographic variable to characterize the study population's socioeconomic background and health literacy potential, which may influence intervention adherence and outcomes in geriatric care. Data were categorized as primary school or below, junior high school, senior high school, and junior college or above. Occupation type was documented as an indicator of lifelong socioeconomic status and cognitive engagement patterns, both recognized as relevant factors in aging trajectories and frailty development. Data were classified as mental or physical work. Living arrangement was also analyzed as a social determinant affecting social support systems, which is particularly relevant for elderly populations' health outcomes and intervention compliance. Data were classified as living alone or with others. All data were obtained through direct self-reporting during structured baseline assessments. Each variable was independently analyzed without serving as a proxy for other social constructs.
Population characteristics	The study population consisted of elderly cardiovascular patients with comprehensive covariate documentation. Demographically, participants (mean age 79.5±5.4 years) included 42.6% males with diverse educational backgrounds (primary school to junior college) and occupational histories (mental/physical work), while 14.2% lived alone. Clinically, prevalent conditions included hypertension (84.5%), coronary artery disease (71.5%), and heart failure (27.1%), with 73.0% using ≥5 medications. Systematic geriatric assessments confirmed universal frailty (Fried Frailty Phenotype score 3.5±0.6), impaired physical function (Short Physical Performance Battery score 6.1±2.7), and maintained basic independence (Instrumental Activities of Daily Living score 6.2±2.2). All data were collected through structured researcher-administered evaluations during hospitalization, with clinical parameters verified through medical records. No genotypic information was obtained in this study.
Recruitment	Participants were recruited through systematic daily screening of all newly admitted elderly patients from cardiac wards across three tertiary hospitals in Beijing. A consecutive enrollment approach was implemented, whereby all patients meeting the preliminary inclusion criteria were approached for participation, to minimize selection bias. Potential biases include the exclusion of patients unable to complete rehabilitation exercises or the SPPB assessment, as well as those with severe comorbidities or a life expectancy of less than 12 months, which may limit the generalizability of findings to the broader population of frail elderly patients with cardiovascular diseases. Additionally, the requirement for long-term Beijing residency may introduce geographic and socioeconomic biases. These factors likely result in a study population that is more stable and medically compliant than the general frail population with cardiovascular diseases, potentially leading to an overestimation of intervention effectiveness in real-world settings. The impact of these biases is partially mitigated by the pragmatic trial design and the multi-center recruitment strategy across three distinct tertiary hospitals.
Ethics oversight	The study protocol was approval by the Ethics Committee of Beijing Hospital (2019BJYYEC-045-02), the Ethics Committee of the General Hospital of the People's Liberation Army (S2019-208-01), and the Ethics Committee of Beijing Tsinghua Changgung Hospital (18166-0-01).

Note that full information on the approval of the study protocol must also be provided in the manuscript.

Field-specific reporting

Please select the one below that is the best fit for your research. If you are not sure, read the appropriate sections before making your selection.

☒ Life sciences ☐ Behavioural & social sciences ☐ Ecological, evolutionary & environmental sciences

For a reference copy of the document with all sections, see nature.com/documents/nr-reporting-summary-flat.pdf

Life sciences study design

All studies must disclose on these points even when the disclosure is negative.

Sample size	The sample size was calculated based on a two-sided, two-sample t-test (power = 80%, α = 0.05). For the Fried Frailty Phenotype (FFP) score, we referenced both Cameron et al. (2013, reference 35) and Ng et al. (2015, reference 36). Cameron et al. reported a mean reduction of 0.80 points (SD 1.19) in the intervention group and 0.41 points (SD 1.02) in the control group at 12 months, corresponding to a between-group mean difference of 0.41 points. Ng et al. (2015) reported a between-group difference in frailty score reduction of approximately 0.4 to 0.6 points favoring the intervention groups, which is consistent with the effect size reported by Cameron et al. (2013). Based on these data, we assumed a between-group difference of 0.39 points with a common standard deviation of 1.1. Using a two-sided two-sample t-test (80% power, α = 0.05), approximately 250 participants (125 per group) were required. For the Short Physical Performance Battery (SPPB) score, Cameron et al. reported a mean increase of 0.52 points (SD 2.47) in the intervention group and a mean decrease of -0.98 points (SD 2.30) in the control group at 12 months, corresponding to a between-group mean difference of 1.4 points. Assuming a common standard deviation of 2.4, approximately 94 participants (47 per group) were required. The larger of these estimates (250 participants) was used as the basis for our sample size. Considering an anticipated attrition rate of 20%, the target sample size was set at 320 participants (160 per group), which was successfully recruited.
Data exclusions	In the intention-to-treat (ITT) analysis, all 333 randomised participants were included, with missing primary outcome data handled using the expectation-maximization imputation method as pre-specified in the statistical analysis plan. No participants were excluded from the ITT analysis. In the per-protocol set (PPS) analysis, 81 participants were excluded for the following pre-specified reasons: voluntary withdrawal (n=25), loss to follow-up or missing primary outcome data (n=56). Supplementary analyses demonstrated comparable baseline characteristics between participants who completed the study and those lost to follow-up (Supplementary Tables 2 and 3), supporting the impartiality of these exclusions. Full details of participant flow are provided in Figure 1.
Replication	This study reports the findings of a pre-specified multicentre randomised controlled trial. Reproducibility was ensured through a predefined study protocol, standardized intervention procedures across participating centers, investigator training, blinded outcome assessment, centralized data management using an electronic data capture (EDC) system, and a pre-specified statistical analysis plan. Trial data were checked for completeness, consistency, and accuracy before database lock, and statistical analyses were independently verified. All reported analyses were reproducible from the original trial dataset. No planned analyses failed replication.
Randomization	After obtaining consent and completing baseline assessments, participants were randomly assigned to either the intervention or control group in a 1:1 ratio using a central computer system within the EDC system. Simple randomisation without stratification or blocking was used. The randomisation sequence was generated to ensure no manual interference. The allocation sequence was statistically unpredictable, and group assignment was only revealed after participant enrolment, informed consent, and baseline assessments were completed.
Blinding	Blinding was maintained for endpoint evaluators and data analysts; however, due to the nature of the interventions, participants and multidisciplinary treatment teams were not blinded.

Reporting for specific materials, systems and methods

We require information from authors about some types of materials, experimental systems and methods used in many studies. Here, indicate whether each material, system or method listed is relevant to your study. If you are not sure if a list item applies to your research, read the appropriate section before selecting a response.

Materials & experimental systems

n/a	Involved in the study	
☒	☐	Antibodies
☒	☐	Eukaryotic cell lines
☒	☐	Palaeontology and archaeology
☒	☐	Animals and other organisms
☐	☒	Clinical data
☒	☐	Dual use research of concern
☒	☐	Plants

Methods

n/a	Involved in the study	
☒	☐	ChIP-seq
☒	☐	Flow cytometry
☒	☐	MRI-based neuroimaging

Clinical data

Policy information about clinical studies

All manuscripts should comply with the ICMJE guidelines for publication of clinical research and a completed CONSORT checklist must be included with all submissions.

Clinical trial registration	The study protocol was pre-registered with the China Clinical Trials Registry (ChiCTR1900022623) on April 19, 2019, prior to the first patient enrollment, and patient enrollment is complete. The full protocol is available at https://www.chictr.org.cn/showproj.html?proj=38141.
Study protocol	The full trial protocol can be accessed in the supplementary information. The full protocol is available at https://www.chictr.org.cn/showproj.html?proj=38141.

Data collection	This trial was conducted from June 2019 to June 2022 across three tertiary hospitals in Beijing, China (Beijing Hospital, the Sixth Medical Centre of PLA General Hospital, and Beijing Tsinghua Changgung Hospital). Potential participants were systematically identified by the research team through daily screening of all newly admitted elderly patients from the cardiac wards of these hospitals. Recruitment occurred from June 2019 to June 2021, with follow-up assessments at 3, 6, and 12 months post-randomisation. The first patient was enrolled on 3 June 2019, and the last patient was enrolled on 10 June 2021. The final follow-up was completed in June 2022. Baseline data were collected during the index hospitalisation; subsequent assessments were conducted through a combination of in-person hospital visits and telephone follow-ups. The study conducted in accordance with the Declaration of Helsinki. The study protocol was approval by the Ethics Committee of Beijing Hospital (2019BJYYEC-045-02), the Ethics Committee of the General Hospital of the People's Liberation Army (S2019-208-01), and the Ethics Committee of Beijing Tsinghua Changgung Hospital (18166-0-01).
Outcomes	The primary and secondary outcome measures were pre-defined as follows: (1) The co-primary outcomes were the Fried Frailty Phenotype (FFP) score and Short Physical Performance Battery (SPPB) score at 12-month follow-up. The FFP score was selected as a validated syndromic measure of frailty status assessing five biological criteria (unintentional weight loss, exhaustion, weakness, slowness, low physical activity), with frailty defined as meeting ≥3 criteria. The SPPB was chosen as an objective performance-based measure of physical function evaluating balance, gait speed, and lower-body strength through standardized tasks. Both measures were administered at baseline, 3, 6, and 12 months by trained assessors using established protocols.<br>(2) Pre-specified secondary outcomes included:<br>a. Clinical Frailty Scale (CFS) – a global frailty assessment tool<br>b. Basic Activities of Daily Living (BADL) – assessing disability in self-care tasks<br>c. EQ-5D quality of life index – though ultimately excluded from systematic baseline assessment due to feasibility constraints<br>d. Medical insurance expenses – tracking economic impacts<br>e. Fall events – monitoring safety outcomes<br>f. Nutrition Risk Screening (NRS-2002) – evaluating nutritional status<br>g. All-cause mortality and non-elective hospital readmission – documenting clinical events, with readmission defined as unplanned admission via emergency departments or clinical visits.<br>(3) Additional measures included Instrumental Activities of Daily Living (IADL), Hospital Anxiety and Depression Scale-Anxiety (HADS-A), and Pittsburgh Sleep Quality Index (PSQI), which were analyzed as pre-specified exploratory endpoints despite not being designated as primary or secondary outcomes.<br>All assessments were conducted by blinded evaluators following standardized protocols, with timepoints clearly specified in the study design. The statistical analysis plan detailed handling of all outcome measures.

Plants

Seed stocks	*Report on the source of all seed stocks or other plant material used. If applicable, state the seed stock centre and catalogue number. If plant specimens were collected from the field, describe the collection location, date and sampling procedures.*
Novel plant genotypes	*Describe the methods by which all novel plant genotypes were produced. This includes those generated by transgenic approaches, gene editing, chemical/radiation-based mutagenesis and hybridization. For transgenic lines, describe the transformation method, the number of independent lines analyzed and the generation upon which experiments were performed. For gene-edited lines, describe the editor used, the endogenous sequence targeted for editing, the targeting guide RNA sequence (if applicable) and how the editor was applied.*
Authentication	*Describe any authentication procedures for each seed stock used or novel genotype generated. Describe any experiments used to assess the effect of a mutation and, where applicable, how potential secondary effects (e.g. second site T-DNA insertions, mosiacism, off-target gene editing) were examined.*
