# Supplementary material for: Multidisciplinary intervention in frail elderly patients with cardiovascular diseases: a multicentre, single-blinded, randomised controlled trial
Source: Nat Commun. 2026 Aug 31;17:10340. doi: 10.1038/s41467-026-77277-7 (PMC13623951; doi:10.1038/s41467-026-77277-7)
Supplement: Supplementary file 3 [file 41467_2026_77277_MOESM3_ESM.pdf]

nature portfolio

Peer Review File

Multidisciplinary Intervention in Frail Elderly Patients With Cardiovascular Diseases: a multicentre, single-blinded, randomised controlled trial

Corresponding Author: Dr Hua Wang

This file contains all reviewer reports in order by version, followed by all author rebuttals in order by version.

Version 1:

Reviewer comments:

Reviewer #1

(Remarks to the Author)
Thanks for the opportunity to review the manuscript titled: “Impact of Multidisciplinary Intervention on Frailty, Functionality, and Readmission for Frail Elderly Patients With Cardiovascular Diseases: multicentre, single-blinded, randomised controlled trial”. This is an important topic in the context of an ageing population and the increasing number of adults with CVD entering advanced age.

Some concerns I have are around the reproducibility of the study methods. The current form lacks details of the target population, recruitment process, assessment procedures, details of the intervention, and implementation. It is unclear in the stats analysis if and how confounding factors are addressed. The rationales for subgroup analyses need to be clearer.

Results section: Including the adjusted effect size for intention-to-treat and per-protocol analyses is required. Adverse events and the adherence rate throughout the study duration are missing in the results section.

Discussion section: How do we know if the intervention program impacts the improvement, or is it the frequency (or quality) of the communications with the health professionals in the intervention group? Did the control group have a similar frequency of communications with the study team? More discussion on the acceptability and adherence of the intervention is required; it would be good to include a discussion of the individual components. The authors acknowledge the limitations of the absence of the cost-effectiveness component of the study.

Conclusion: The conclusion appears relevant to the results presented in the current version. However, whether the intervention program's results provide sufficient statistical evidence to justify rejecting the null hypothesis remains unclear.

Reviewer #2

(Remarks to the Author)
Chai et al. report the results of a year-long followup of a multi-modal intervention using education, diet, physical therapy, and traditional Chinese medicine with control of polypharmacy for treatment of frailty in patients with cardiovascular diseases (CVD). As the authors make clear, their approach is predicated on previous work, primarily in outpatient settings, to treat sarcopenia and frailty. A notable point about this work is recruitment during inpatient hospitalizations for CVD, which led to a more severely ill cohort of patients with CVD specifically. The application of culturally appropriate interventions to the Chinese population is also a novel point.

The analysis seems to have been done appropriately and the results suggest that there was clinically relevant and statistically significant improvement in frailty (using the Fried criteria), functon(SPPB and IADLs), and hospitalizations. There was a numeric trend on mortality but it was not significant, probably due to small numbers of events in the two groups. Limitations are clearly described.

My only concern is that the effect seemed to wear off between 6 and 12 months of followup, and this is not addressed in the discussion (see Figure 3). Both groups had strong improvements after hospital discharge, consistent with expectations that going home from the hospital is a good idea and that most people recover from the acute illness to some extent by 3 months. Both groups also maintained that improvement to 6 months in terms of FFP, and both had further improvement in SPPB to 6 months. It is in the next 6 months that the groups diverge, with worsening results between 6 and 12 months that were more notable in the control group than the actively treated group.

A true skeptic could say that there was no benefit to RENAP after 6 months, but a more likely interpretation is that the general improvement caused by recovering from the hospitalization (and reaping the benefits of the inpatient treatment) swamped the effect of the RENAP intervention until after month 6; only once that post-hospitalization effect receded were the effects visible.

Although the investigators "adjusted the interventions (line 449)" during the 3- and 6-month visits in the active group, one wonders whether a more comprehensive refresher treatment with the RENAP protocol at month 6 would have had a larger protective effect at 1 year. This is a key point, since knowing how often to intervene is as important as knowing how to intervene to treat or prevent frailty. This would take a follow up study to ascertain, but at least it should be discussed.

The effect of adherence is also worth additional comment in the discussion. On comparing Fig 3 with Suppl Fig 1, we see a roughly 20% higher difference in FFP (difference between groups at 12 months = 0.8 vs. 0.97, ITT vs PPS) and a larger 45% difference in SPPB (difference 1.84 in ITT vs. 2.68 in PPS). This should be more clearly explained in the Results section, including clearly stating that the primary results are from the ITT population (lines 151ff), and directly comparing the sample sizes and effect sizes in ITT vs. PPS.

Reviewer #3

(Remarks to the Author)
Dear authors,

This is a very interesting study and paper on very timely topics and growing concerns for healthcare and society (frailty, CVD, and outcomes). Having read the response to previous reviews, I also commend the authors for their very frank and thoughtful responses. I do believe this paper can be of great interest to a broad range of readers, but I also have a number of concerns and queries, as below:

Introduction:

Please add a citation for lines 91, 99, 108.
Line 116: Why is this acronym RENAP, or what does RENAP stand for? Please write it out fully first, then use the abbreviation, and cite it if any publication for it already exists.

Results:
Lines 144-145: I commend the authors for including the PSQI. Sleep is a major contributor to physical and mental health and well-being and poor raises the risk and increases the severity of a number of health conditions associated with frailty and CVD.
Line 146: Was the loss to follow-up (LTF) evenly distributed between groups? What was the cause of the LTF?
Line 187: This is likely true but that statement is not a result, it is an conjecture on the result, and that belongs in the discussion section, not the results.

Discussion:
Line 204: I suggest function, rather than “functionality.”
Line 208: This sentence could use some editing. The beginning is a bit unclear: “Our study show integrating frailty assessments with targeted interventions may lead to significant improvements in both clinical and functional outcomes, addressing the growing challenges of CVD and frailty on a global scale.” Does this mean that frailty assessments paired with targeted interventions can lead to the improvements?
Please add a citation for line 214
Line 219: Are all of these targeting insomnia? Does this population have a high prevalence of insomnia, or Insomnia Disorder? This should be mentioned as well in the introduction so that it is more sensible here. Otherwise, this comes as a surprise (insomnia). Same for constipation. How is this related to frailty or CVD? Are the RENAP intervention components all targeting insomnia and constipation?
Line 221- this leaps to anxiety levels. But what was the result in terms of insomnia and constipation? How did you measure constipation in the sample?
Line 227: acupoint massage is never introduced in the intro- what is the background for it. Why did you add this as an intervention?
Line 233: I don’t see how this relates to your study as your participants had frailly. I see the connection in terms of CVD, but the issue is frailty as well.
Line 236: did they investigate this in persons with frailty? If not, this really doesn’t add anything and doesn’t need to be added. If so, say that they investigated this in older adults with frailty.
Line 252: here you could add the line about future studies using a larger sample size to investigate this.
Line 255, line 257, line 261 (susceptibility), line 263 : these definitely need citations. I don’t dispute these statements, but whenever you state something as a fact, it needs to be supported with an appropriate citation.

Line 270: Please explain a bit how this is culturally adaptable.
Line 274: perhaps your research contributes, rather than demonstrates?
Line 281: Regular exercise also improves sleep, which increases immune and metabolic function, organ repair and body tissue repair, cognition, mood, and anxiety. There is also a bidirectional relationship between sleep and exercise. These would be important to mention (and cite) as well. Exercise also improves cognition and mood/anxiety independently of sleep and nutrition.
Line 287: This must be cited. It needs to be supported by citations. Also, are you referring to insomnia symptoms, or insomnia disorder (difficulty falling or staying asleep ≥3 times per week for >3 months with daytime impairment)? They are different.
Line 307: how was missing data handled? I expect this will be in the methods.
Line 307: Also, you defined adherence as better than 50% adherence, but that is a low threshold. Why did you choose 50% and not a higher threshold, like 70 % or greater? The low adherence could certainly have biased the outcomes. See: https://www.jclinepi.com/article/S0895-4356(24)00366-4/fulltext

Line 338: adherence to what? Please specify.
Line 341: At times you say adherence, at times you say compliance. Please use one consistently. These are often used interchangeably but have different connotations and can be misunderstood by different readers. I suggest you stay with the term “adherence” throughout.
Lines 364-370: This section is repetitive. It feels as if you are repeating the last part of your discussion. This should be edited to avoid that.

Methods:
Line 379: they affirm these for the implementation of the protocol and conduct of the trial? If so, please specify that.
Lines 412-417: who increased the intensity, and how? How was this assessed or determined, and by whom? What constitutes a “safe level?”
Lines 425-428. What is TCM/what does it stand for? Why did you choose that in particular? Also, please add citations for these. What is a TCM tongue diagnosis, and what does it or the pulse method diagnose?
Lines 428-430: These should be described somewhere, even if in supplementary materials.
Lines 441-442: Unified approach to what? Please specify.
Lines 449-453: see my comments about this in the discussion section comments, above.

Version 2:

Reviewer comments:

Reviewer #2

(Remarks to the Author)
Thank you for the clear responses - no further concerns.

Reviewer #3

(Remarks to the Author)
Dear authors,

Thank you for your very frank and thoughtful response to my questions and comments, as well as to the other reviwers'. These have improved the manuscript considerably and have addressed my concerns. In particular, the more nuanced and balanced discussion and conclusions now address my previous concerns. The reproducibility of the methods is also significantly improved.

Reviewer #4

(Remarks to the Author)
As Reviewer 1 was unavailable, I have been asked to assay your replies. Here is how I see them, having first read the paper independently of any of the reviews.

Briefly this is an RCT of a truly novel multidimensional intervention: a set of posture, breathing, pelvic floor, and stretching/limb movements termed “rehabilitation exercises”, patient education, nutritional guidance, acupoint massage, and polypharmacy management. It benefited in persuasive ways (moving the needle on the Fried Physical Frailty Phenotype, by the Short Physical Performance Battery score (SBBP), and a difficult-to-change readmission rate. Several points are of note here, including that the intervention was made to be culturally appropriate (the acupoint massage (self-administered) is based on traditional Chinese Medicine, with useful details in the Methods, and especially in the Supplementary Methods. Also, demonstrating benefits of polypharmacy is proving to be quite tricky, enough for achieving modest gains such as reducing anticholinergic burden.
Strikingly, at one year, the intervention group showed a lower degree of frailty than they had at the start by FPFP score, with a significant between-group difference favoring the intervention group. This is bolstered by better SPPB scores and by a similar Clinical Frailty Scale profile, together with superior hospitalization-free survival, but not with better survival overall. These factors in an RCT evidence of a statistically significant, clinically detectable, biologically plausible impact, seen across multiple outcomes and converging with existing evidence, all point to the intervention making a clinically meaningful

difference.

Points 1, 2 As it now stands, I have no concerns about the description of how participants were recruited, or how the data were analysed. It appears that the Reviewer's comments were spot on, and indeed the paper has improved as a result. It's especially reassuring to know too that the subgroups analyses were considered a priori and not post hoc.
Point 3. If Reviewer One, by "adjusted effects size" meant something along the lines of Standardized Response Means, then constructing 95% confidence intervals for the differences is not exactly the same. A standardized response mean (or Hedge's G or Cohen's d) as an estimate of effect size tells you the size of the effect. The area is famously controversial. Here is a detailed guide. Ellis PD.(2012) The Essential Guide to Effect Sizes. Cambridge University Press ISBN: 9780511761676 DOI: https://doi.org/10.1017/CBO9780511761676 Nat Communs likely has a statistical reviewer who can point to an acceptable answer. (Beware though: the most vitriolic review I've ever had came from an aggrieved statistical reviewer over which effect size was most appropriate.)
Points 4 and 5: To me, these read as variants of the standard critique that RCTs might be valid, but not generalizable. In my view, the authors' answers are entirely appropriate.
The points covered from pages 6-21 indicate a very careful read, and equally careful and unobjectionable replies, in my estimation. The single objection is the issue around whether one can ever say that a patient is becoming more frailer (or frailer). In my experience, it is indubitable that people can become frailer – and your data show that they can become less frail too. I respect that, wisely in my view, you elided the issue while still making clear that you employ quantitative reasoning in making your comment – in relation to the differences in the mean number of frailty items present in the two groups. .

This leads me I'd make one other comment. I paid attention that the other secondary measures included the Clinical Frailty Scale, and that it too showed significant improvement. (Let me declare here that I'm Kenneth Rockwood, the originator of the CFS.) I'd be inclined to highlight this finding in the Discussion, Here's why. The CFS is derived from the deficit accumulation approach to frailty, also introduced in 2001. The field is often said to be "plagued" by having the two definitions. Here, you can join the comparatively small number of papers that report interventions with estimates in relation to both measures.

I also note though that the Clinical Frailty Scale, as with all the other outcomes measures, save the Fried Physical Frailty Phenotype, goes uncited. Given that there are hundreds of variation of the Fried Physical Frailty Phenotype (PMID: 25846451) it would be useful to know exactly how it was operationalised here. As far as I can see, the only modification was, by inference from the Statistical Analysis Plan (Appendix 3, p44 and for example, Table 1, main paper, p34, where it is reported as a score with mean ± SD,) was that for analytic purposes, it was treated as a six-point frailty index (that is to say, from zero to five features). This contrasts with the 2001 report (your ref 2) where it is presented as a categorical variable. To avoid confusion, here is the version of the CFS that the authors appear to have used: Rockwood K, Theou O. Using the Clinical Frailty Scale in Allocating Scarce Health Care Resources. Can Geriatr J. 2020 Sep 1;23(3):210-215. doi: 10.5770/cgj.23.463. PMID: 32904824; PMCID: PMC7458601.

Kenneth Rockwood, Halifax, Canada

Reviewer #5

(Remarks to the Author)
This review considers the use of statistics in the paper by Chai et al, a randomised controlled trial of a multicomponent intervention for frail elderly patients with CVD.

Overall, this appears to be a well designed and conducted trial. Generally, the statistical methods are good, and everything is quite well described and presented. I do have a few observations, which should be easily remedied. The following is ordered according to how I encountered things in the paper.

Regarding the randomisation, I would like to see a little more detail about blocking and stratification, if these were done. Also, can the authors be sure that the allocations were effectively concealed, so that those recruiting participants were unaware of what the next allocation was going to be?

The results section begins with a lengthy section about the balance of the baseline data. For one thing, it is generally not advised to report statistical tests of baseline data (given the randomisation, it is known that the null hypothesis is true). In this case, it would be sufficient to state that there were no major differences between the groups, with reference to the table. It is not necessary to list all the baseline factors which were looked at. And, I would advise against reporting statistical results as "p>0.05"; there is a world of difference between p=0.06 and p=0.99.

When looking at the statistical comparisons in Table 1, I was not able to replicate the p-value for BMI (I got t=1.56, p=0.119), assuming a standard t-test was done, and given the means and SDs reported. I was not expecting an exact match, due to rounding of the summary data reported, but this is very different. Also, for age, I got p=0.242 (this time, a smaller p-value). So, whilst I do not recommend statistical tests of baseline data, I would suggest these results need to be checked.

I then looked as the categorical variables. The footnote to the table reports these were compared with chi-square tests, whereas the methods section of the paper also mentions Fisher tests. Looking at a couple of p-values, these all seem to be Fisher test p-values. This is fine – I would routinely use Fisher tests rather than chi-square. If this is what was done, the statistical methods and table footnotes should reflect this.

Line 162, should this be reported as an “adjusted mean difference”, rather than simply “mean difference”?

The PPS is defined as those completing the intervention and having outcome data, but what does “completing the intervention” mean for the control arm?

Line 176, I think the control group showed an increase of 0.2 points, not a decrease.

It is probably worth noting that the analysis of mortality had very little power, and whilst the confidence interval is very wide, the point estimate is the same as for hospitalisations. Simply saying that it was not statistically significant is perhaps a little down-beat.

I note the slightly higher fall rate in the intervention arm, perhaps not unexpected if the intervention leads to greater mobility. Did any falls lead to hospitalisations?

For the subgroup analyses, it is not sufficient to simply conduct the analyses within subgroups and comment on which analyses were statistically significant or not. It is better to fit regression models with interaction terms to assess whether there is evidence that the intervention effect differs between subgroups. Only if the test of interaction is significant (some people use a more relaxed p<0.1 when looking at interactions) does it make sense to comment on how the effects differ between groups. I would include a column of interaction p-values to the right of Figure 3.

For the sample size calculations, in order to be able to replicate the calculations, the reader needs the assumed means and standard deviations (or mean difference and common standard deviation). These should be provided with reference(s) to justify.

When fitting Cox models, one should check the proportional hazards assumption. Was this done?

Line 576: “multivariable” is more correct.

Version 3:

Reviewer comments:

Reviewer #5

(Remarks to the Author)
I thanks the authors for their careful consideration of my original comments. I am satisfied with all of their responses and have no further comments to make.

Regarding a comment from another reviewer, I prefer to see effect estimates (or "effect sizes") reported on the original scale (as in this paper), rather than being standardised, though I do appreciate the worth of standardised versions in some situations.

(Remarks on code availability)
I am not a SAS user.

Open Access This Peer Review File is licensed under a Creative Commons Attribution 4.0 International License, which permits use, sharing, adaptation, distribution and reproduction in any medium or format, as long as you give appropriate credit to the original author(s) and the source, provide a link to the Creative Commons license, and indicate if changes were made.
In cases where reviewers are anonymous, credit should be given to 'Anonymous Referee' and the source.
The images or other third party material in this Peer Review File are included in the article's Creative Commons license, unless indicated otherwise in a credit line to the material. If material is not included in the article's Creative Commons license and your intended use is not permitted by statutory regulation or exceeds the permitted use, you will need to obtain permission directly from the copyright holder.
To view a copy of this license, visit https://creativecommons.org/licenses/by/4.0/

RESPONSE TO REVIEWERS

We sincerely thank the reviewers for their insightful and constructive feedback, which has been invaluable in strengthening our manuscript. We have carefully addressed each comment point-by-point, as detailed below. The revisions have significantly enhanced the clarity, rigor, and overall impact of our work. We believe the manuscript is now substantially improved and hope it meets the high standards of Nature Communications.

We have provided two versions of the revised manuscript: one with tracked changes (Manuscript_Revised_with_Changes) and a clean version (Manuscript_Revised_Clean). The line numbers referenced here correspond to the clean version without revision marks.

Reviewer #1:

Thanks for the opportunity to review the manuscript titled: "Impact of Multidisciplinary Intervention on Frailty, Functionality, and Readmission for Frail Elderly Patients With Cardiovascular Diseases: multicentre, single-blinded, randomised controlled trial". This is an important topic in the context of an ageing population and the increasing number of adults with CVD entering advanced age.

Some concerns I have are around the reproducibility of the study methods. The current form lacks details of the target population, recruitment process, assessment procedures, details of the intervention, and implementation. It is unclear in the stats analysis if and how confounding factors are addressed. The rationales for subgroup analyses need to be clearer.

Results section: Including the adjusted effect size for intention-to-treat and per-protocol analyses is required. Adverse events and the adherence rate throughout the study duration are missing in the results section.

Discussion section: How do we know if the intervention program impacts the improvement, or is it the frequency (or quality) of the communications with the health professionals in the intervention group? Did the control group have a similar frequency of communications with the study team? More discussion on the acceptability and adherence of the intervention is required; it would be good to include a discussion of the individual components. The authors acknowledge the limitations of the absence of the cost-effectiveness component of the study.

Conclusion: The conclusion appears relevant to the results presented in the current version. However, whether the intervention program's results provide sufficient statistical evidence to justify rejecting the null hypothesis remains

unclear.

RESPONSE:

*Thanks for the thoughtful, positive, and constructive feedback on our manuscript. We have carefully considered all the points and believe that the detailed methodology provided in our **Supplementary Material** addresses most of the concerns. Below, we provide a point-by-point response to your comments, with references to the relevant sections in the main manuscript and supplemental file.*

Comment 1: Methods section: Some concerns I have are around the reproducibility of the study methods. The current form lacks details of the target population, recruitment process, assessment procedures, details of the intervention, and implementation.

***Response**: Thanks for emphasizing the importance of methodological reproducibility and statistical rigor. We have thoroughly revised the manuscript to provide greater clarity and detail regarding the target population, recruitment process, assessments, intervention, and statistical adjustments. The key enhancements are summarized below, and comprehensive protocols remain available in the Supplemental Material.*

1.Target Population & Recruitment Process:

（1）Clarification in Manuscript: We have now explicitly defined the recruitment strategy in the main text (Page 17, Lines 404-407), specifying that participants were consecutively screened from cardiac wards during hospitalization.

（2）Detailed Criteria: The inclusion and exclusion criteria have been further elaborated (Pages 17-18, Lines 407-416), including specific examples of "severe comorbidities" (e.g., metastatic cancer, end-stage renal disease) to enhance reproducibility across settings.

2. Assessment Procedures & Intervention Implementation:

（1）Standardized Protocols: All assessment tools and timepoints are detailed in the Methods section. We also explicitly state that the SPPB was administered according to the standardized National Institute on Aging protocol (Page 22, Lines 516-517).

（2）Intervention Details: The RENAP intervention is now thoroughly described, including:

Exercise progression criteria and definition of safe level (Pages 18-19, Lines 436-446);

Nutritional prescription and practical guidance for patients (Page 19, Lines 451-460);

TCM assessment and acupoint massage procedures (Pages 19-20, Lines 461-476);

Polypharmacy management process involving clinical pharmacists (Page 20, Lines 477-483).

（3）Reproducibility Assurance: We emphasize that reproducibility was ensured through detailed flowcharts (Figure 4) and standardized operating procedures (Supplementary Material, Pages 12-28, Appendix 1), as noted in the Methods (Page 18, Lines 433-435).

3. Supplemental Material:

For full reproducibility, the Supplemental Material includes:

（1）The complete study protocol (Supplementary Material, Pages 35-49, Appendix 3) with detailed eligibility criteria and recruitment workflows (Supplementary Material, Appendix 3, Pages 38-40, Sections 2.1-3.3.1).

（2）Step-by-step SOPs for each RENAP component (Supplementary Material, Pages 12-28, Appendix 1).

（3）The "Holistic Health Tracker" used for adherence monitoring (Supplementary Material, Pages 29-34, Appendix 2).

We believe these revisions ensure that our methods are fully transparent and reproducible.

Comment 2: It is unclear in the stats analysis if and how confounding factors are addressed. The rationales for subgroup analyses need to be clearer.

Response: *Thanks for this critical comment regarding statistical rigor. We are pleased to clarify our approach to addressing confounding and the rationale for subgroup analyses, which has been further strengthened in the revised manuscript.*

1. Addressing Confounding Factors

To ensure the robustness of our findings, we employed several statistical methods to account for potential confounding:

(1) For Primary Continuous Outcomes (FFP and SPPB scores): The primary analysis used mixed-effects models for longitudinal data. These models inherently adjust for baseline values of the outcome variable (Page 23, Lines 552 and 556-558). In direct response to this comment, we have now performed and reported a specific multivariate analysis that explicitly adjusts for baseline scores. The results, presented in the manuscript (Pages 7-8, Lines 158-166), robustly confirm the significant treatment effect, with the between-group differences remaining highly significant (both $P < 0.001$).

Furthermore, as a randomized controlled trial, our study design inherently controls for potential confounders through the randomization process, ensuring baseline comparability between the intervention and control groups. Minor imbalance was observed in BMI, which was adjusted in Time-to-Event Outcomes analysis. Based on clinical judgment, we concluded that these limited imbalances were unlikely to exert a significant influence on the prognosis, and thus further adjustment was deemed unnecessary.

(2) For Time-to-Event Outcomes (Readmission and Mortality): We used Cox proportional-hazard regression models. As pre-specified in our Statistical Analysis Plan (Supplementary Material, Appendix 4, Page 54), a multivariate Cox model was employed, incorporating key clinical and demographic subgroup variables as covariates to adjust for potential confounding within the subgroups when examining these clinical endpoints. The variables included age, sex, BMI, coronary heart disease, atrial fibrillation or flutter, heart failure, diabetes mellitus, and our intervention. The results were shown in supplemental materials (Supplementary Table 5).

2. Rationale for Subgroup Analyses

The pre-specified subgroups (e.g., age, sex, heart failure, diabetes) were chosen based on a priori clinical relevance rather than post-hoc data exploration. Our rationale, now explicitly stated in the manuscript (Page 24, Lines 569-572), is that these factors represent common comorbidities and demographic characteristics in our target population of frail elderly CVD patients, which we hypothesized a priori could modify the response to a multidimensional intervention like RENAP.

For instance, we theorized that patients with heart failure might derive different benefits from the rehabilitation component compared to those without. The consistent treatment effects observed across most subgroups (as visually presented in forest plots in Figure 3 and Supplementary Figure 2) enhance the generalizability and clinical applicability of our findings.

By pre-specifying these analyses, employing models that adjust for confounding, and clearly stating our clinical rationale, we have ensured that our statistical approach is both rigorous and transparent.

Comment 3: Results section: Including the adjusted effect size for intention-to-treat and per-protocol analyses is required. Adverse events and the adherence rate throughout the study duration are missing in the results section.

***Response**: Thank you for this important suggestion. We agree that the explicit presentation of adjusted effect sizes, adherence, and safety data is critical for a comprehensive interpretation of the trial results. In response, we have now integrated these key elements directly into the main Results section of the revised manuscript.*

1. Adjusted Effect Sizes for ITT and PPS Analyses:

*We now explicitly state that the primary analysis was performed on the Intention-to-Treat (ITT) population with the following text: "*In the Intention-to-Treat (ITT) analysis, at the 12-month follow-up...*"(Page 7, Line 158) and present the adjusted between-group mean differences for the primary outcomes: "*The mean FFP score decreased by 1.8 points in the intervention group compared to a decrease of 1.1 points in the control group, resulting in a between-group mean difference of -0.79 points (95% CI: -1.11 to -0.46; P < 0.001). Additionally, the mean SPPB score improved by 0.4 points in the intervention group, while it decreased by 1.6 points in the control group, leading to a between-group mean difference of 2.02 points (95% CI: 1.04 to 2.99; P < 0.001).*" (Pages 7-8, Lines 160-165)*

*Furthermore, we immediately follow this by reporting the corresponding effect sizes from the Per-Protocol Set (PPS) analysis, providing a direct comparison: "*In the Per-Protocol Set (PPS) analysis, which included participants completing the intervention and having outcome assessments (n=120 for RENAP, n=132 for control), the intervention group showed significant improvements in both FFP and SPPB scores... The between-group difference in FFP and SPPB at 12 months was 0.90 (95% CI: -1.27 to -0.52; *P* < 0.001) and 2.78 (95% CI: 1.65 to 3.90; *P* < 0.001) in the PPS analysis,

respectively." (Page 8, Lines 166-172). The complete statistical details for both analyses remain available in Supplementary Table 4.

2. Study Retention and Intervention Adherence:
We have now explicitly reported both study completion (retention rate) and intervention adherence in the main Results section to address distinct aspects of trial conduct.
(1) Study Completion (Retention): We report that "Finally, 252 elderly frail patients completed the one-year follow-up, including 120 (72.3%) in the intervention group and 132 (79.0%) in the control group" (Page 7, Lines 151-153), providing the overall retention rate from the original cohort.
(2) Intervention Adherence: To quantify the execution of the RENAP program, we report the specific adherence rate: "In this study, 120 out of 166 participants (72.3%) randomized to the intervention group met the pre-specified adherence criteria" (Page 14, Lines 332-333). This provides a clear measure of the intervention's feasibility and acceptance, with the adherence criteria defined in the Methods section.
3. Adverse Events (Safety Profile):
The safety profile, specifically regarding fall events, is now summarized in the main Results with the addition of "Regarding safety, the incidence of fall events was 21.0% in the intervention group and 15.3% in the control group (P = 0.236, Supplemental Table 6)" (Page 9, Lines 199-200; Supplementary Table 6). This indicates that the intervention did not significantly increase the risk of falls.

By incorporating these elements into the main text, we ensure that readers have immediate access to the most crucial information regarding efficacy, adherence, and safety, thereby enhancing the transparency and clinical relevance of our reported findings.

Comment 4: Discussion section: How do we know if the intervention program impacts the improvement, or is it the frequency (or quality) of the communications with the health professionals in the intervention group? Did the control group have a similar frequency of communications with the study team? More discussion on the acceptability and adherence of the intervention is required; it would be good to include a discussion of the individual components.

***Response**: Thanks for these excellent points, which have significantly helped us refine the discussion of our findings.*
1. Frequency of Communication and Its Role
We acknowledge this insightful comment regarding the potential confounding effect of contact frequency. This is an important methodological consideration, and we have addressed it explicitly in the revised manuscript.
In the Limitations section, we now state: "The differential contact frequency between groups may represent a potential confounding factor, as the additional communication in the intervention group could have contributed to the observed effects beyond the specific intervention components." (Page 16, Lines 362-365)

However, we posit that the structured, supportive communication was not a standalone factor but an integral and necessary component of the RENAP intervention's implementation. It served as the crucial mechanism for delivering and reinforcing the personalized guidance (e.g., tailoring exercises, troubleshooting nutritional plans, coaching acupoint massage) and for sustaining engagement over the 12-month period. This model reflects real-world clinical practice, where successful management of complex, frail patients inherently requires ongoing support and follow-up. Therefore, we believe the observed improvements are attributable to the content of the RENAP program, which was operationalized through this supportive contact.

2. Acceptability and Adherence of the Intervention

Thank you for this valuable suggestion. We have expanded the Discussion to include a dedicated paragraph on this topic. We now highlight the objective adherence rate, noting that 72.3% of the intervention group met the pre-specified criteria for compliance, demonstrating the feasibility of delivering this complex intervention in a frail population.

We discuss the structured strategies that were key to achieving this adherence, including "the use of a personalized patient handbook for self-monitoring, regular brief telephone support to provide guidance and encouragement, and the tailoring of exercise and dietary plans to individual tolerance and preferences" *(Pages 14-15, Lines334-337). The added paragraph concludes that the high adherence rate suggests that* "the program was not only effective but also feasible and acceptable to the target population, underscoring the potential for integrating such a supported model into routine care pathways" *(Page 15, Lines 339-341).*

3. Discussion of Individual Components

We agree that understanding the contribution of individual components is highly valuable. We have explicitly acknowledged in the Limitations that our study design, while ideal for testing the overall effectiveness of the bundled RENAP program, does not allow us to disentangle the specific contribution of each component (Page 16, Lines 374-375).

We suggest that the observed effects are likely the result of synergistic interactions between the components, which collectively address the multifaceted nature of frailty.

*Importantly, we now state that "*evaluating the efficacy of individual components and their potential synergies represents an important goal for future research, and studies using factorial designs could help identify the most effective elements of multidisciplinary interventions.*" (Page 16, Liens 378-381). This positions our current study as a foundational step and clearly outlines a logical and valuable direction for future work.*

Comment 5: Conclusion: The conclusion appears relevant to the results presented in the current version. However, whether the intervention program's results provide sufficient statistical evidence to justify rejecting the null hypothesis remains unclear.

***Response**: Thank you for the opportunity to clarify the strength of the statistical evidence supporting our conclusions. We agree that robust statistical evidence is paramount for justifying the rejection of the null hypothesis.*

The evidence for the superiority of the RENAP intervention is robust and meets conventional statistical standards for significance:

(1) For the co-primary endpoints, both the Intention-to-Treat (ITT) and Per-Protocol (PPS) analyses demonstrated highly statistically significant results.

(2) As detailed in the manuscript and Supplementary Table 4, the PPS analysis showed a between-group difference in the Fried Frailty Phenotype (FFP) score of -0.90 (95% CI: -1.27 to -0.52; $P < 0.001$) and in the Short Physical Performance Battery (SPPB) score of 2.78 (95% CI: 1.65 to 3.90; $P < 0.001$).

(3) The 95% confidence intervals for these differences exclude the null value of zero, and the P-values are highly significant ($P < 0.001$) for both primary outcomes.

Our study was pre-specified as a superiority trial in the registered protocol and Statistical Analysis Plan. The results above provide strong and sufficient statistical evidence to reject the null hypothesis of no difference between the intervention and control groups for the primary outcomes.

We fully acknowledge in the Discussion the limitations inherent in our pragmatic trial design, such as the inability to disentangle the effects of individual intervention components. However, the core conclusion-that the bundled RENAP intervention is superior to usual care-is grounded in a pre-specified, rigorous, and independently monitored trial, and is supported by unequivocal statistical evidence for its primary objectives.

We have ensured that the Conclusion section of the manuscript reflects this clarity and is squarely based on these statistically robust findings.

In addition, the responses to other suggestions mentioned in the PDF file are as follows:

Line 66: inaccurate terminology

***Response**: Thanks for highlighting this important terminological point. We agree that in the context of our pragmatic clinical trial evaluating a complex intervention in a routine practice setting, 'effectiveness' is a more accurate term than 'efficacy'. Accordingly, we have changed the phrase "evaluate the efficacy" to "*<u>evaluate the effectiveness</u>*" in the manuscript abstract (Page 4, Line 72).*

Line 66: please be specific with the outcomes (this should be aligned with the study protocol)

***Response**: Thank you for this constructive feedback. We agree that alignment with the pre-specified primary outcomes is crucial. The aim of our trial was indeed to evaluate*

the intervention's effect on the Fried Frailty Phenotype (FFP) score and Short Physical Performance Battery (SPPB) score at one year. Considering the word count constraints and the need for clarity in the abstract, we have revised the phrasing to explicitly state the targeted domains of the primary outcomes. The sentence now reads: "...for improving physical function and frailty status in 333 frail elderly inpatients..." (Page 4, Lines 73-74), which directly corresponds to the SPPB (physical function) and FFP (frailty status) measures, respectively. The full details of the primary outcomes are explicitly defined in the Methods section.

Line 73-74: overlapping outcomes? Need a clearer justification why FFP and SPPB were chosen.

***Response**:*
Thanks for this insightful comment, which allows us to clarify the strategic rationale behind selecting both the Fried Frailty Phenotype (FFP) and the Short Physical Performance Battery (SPPB) as co-primary outcomes.
These two instruments were chosen not because they are redundant, but because they capture distinct, complementary, and clinically essential dimensions of the frailty construct, providing a comprehensive assessment of our intervention's impact:
(1) FFP as a Syndromic Classification: The FFP provides a holistic, biologically-oriented diagnosis of the frailty syndrome. It answers the question, "Is this patient frail?" by assessing five key domains (unintentional weight loss, exhaustion, weakness, slowness, low activity). A change in the FFP score indicates a shift in the patient's overall vulnerability and frailty status.
(2) SPPB as a Functional Performance Measure: The SPPB serves as a direct, objective, and continuous measure of lower extremity physical function. It answers the question, "How well can this patient physically perform?" by quantitatively assessing balance, gait speed, and lower limb strength. It is highly sensitive to change and reflects tangible, functional gains.
The Combined Rationale:
Our multidisciplinary RENAP intervention was designed not only to reverse the diagnostic syndrome of frailty (captured by FFP) but also to produce meaningful, functional improvements in mobility and strength (captured by SPPB). Using both outcomes allows us to demonstrate that the intervention successfully alters the underlying frailty phenotype and translates into clinically relevant functional benefits for patients. This dual-assessment strategy provides a more robust and clinically meaningful evaluation of effectiveness than either measure alone.
In response to this comment, we have revised the Methods section (Pages 21-22, Lines 509-516) to include this explicit justification, clarifying the distinct roles of FFP and SPPB in our trial evaluation.

Line 82: "reducing anxiety" was not reported in the result section

***Response**: Thanks for your thoughtful reminder. In our study, the mean HADS-A score*

decreased by 0.4 points in the intervention group, while it increased by 0.9 points in the control group. This resulted in a between-group mean difference of -1.31 points (95% CI: -2.18 to -0.43; P = 0.004). This suggests that the intervention may have an effect on improving anxiety, but as you pointed out, since this was not our pre-specified primary endpoint, we have deleted the relevant content from the manuscript (Page 4, Lines 82-83).

Line 93: ref

***Response**: Thanks for this suggestion. A relevant citation has now been added to the sentence (Page 5, Line 93; Ref 2). Ref 2:* Fried LP, Tangen CM, Walston J, et al. Frailty in older adults: evidence for a phenotype. J Gerontol A Biol Sci Med Sci 2001; 56: 146-56.

Line 116-118: More context about the existing multidisciplinary programmes is require to set the scence for the RENAP programme.
Some background information about the RENAP program is desired.

***Response**: Thank you very much for this insightful suggestion. We agree that providing more context better positions our study within the existing literature and highlights the specific contributions of the RENAP program. We have revised the Introduction to address this point (Page 6, Lines 115-124).The revisions accomplish the following:*
(1) Contextualize Existing Programs: We now acknowledge that while previous multidisciplinary interventions show promise, many were conducted in community settings or lacked a specific focus on the high-risk population of frail elderly inpatients with cardiovascular diseases. Furthermore, the variability in components and intensity across programs creates a need for a standardized, reproducible model.
(2) Introduce and Define RENAP: We explicitly introduce RENAP as a comprehensive, in-hospital initiated, multidisciplinary management program. We clarify that its name is an acronym derived from its five core, protocol-driven components: Rehabilitation, Education, Nutritional guidance, Acupoint massage, and Polypharmacy management.
(3) Highlight Key Differentiators: We emphasize that RENAP is distinguished by its standardized operationalization (detailed in SOPs, Supplementary Material, Pages 12-28, Appendix 1), its focus on the acute care and post-discharge transition, and its use of a structured 12-month follow-up supported by tools like the "Holistic Health Tracker" (Supplementary Material, Pages 29-34, Appendix 2).
We believe these enhancements more clearly establish how RENAP builds upon, yet distinctively advances, existing multidisciplinary approaches by offering a specific and sustainable model for frail cardiology inpatients.

Line 116-118: How is the RENAP programme differ to the CGA?

Response*: Thanks for raising this important question, which allows us to clarify the unique role and contribution of the RENAP program.The Comprehensive Geriatric Assessment (CGA) and the RENAP program serve distinct yet complementary purposes in the care pathway for frail patients:*

（1）CGA is a foundational diagnostic process. It is a multidimensional, interdisciplinary assessment designed to identify a frail older person's medical, psychosocial, and functional capabilities and limitations. Its primary output is a comprehensive problem list and a set of recommendations.

（2）The RENAP program is a structured intervention protocol. It is a proactive, operationalized package of care designed to act upon the problems identified by assessments like the CGA. RENAP actively delivers a bundled therapy consisting of five core components: Rehabilitation, Education, Nutritional guidance, Acupoint massage, and Polypharmacy management.

In essence, the CGA answers the question "What are this patient's needs?" while RENAP provides a standardized, actionable answer to "What do we do to address these needs?" RENAP does not replace the CGA but rather translates its findings into a coordinated, deliverable, and sustained intervention. The novelty of our study lies in evaluating this specific, protocol-driven intervention bundle (RENAP), which is initiated based on CGA principles but constitutes a distinct and proactive treatment phase.

We have refined the relevant section in the Methods section (Page 18, Lines 429-432) to make this conceptual relationship clearer.

Line 161: please report clearly the results from the multivariate analysis.

Response*: Thank you for prompting us to enhance the statistical rigor and clarity of our results reporting. In direct response to this comment, we have now performed a multivariate analysis to provide an adjusted estimate of the treatment effect and have clearly reported these results in the manuscript.*

1. Analysis Methodology: In line with our study objective of comparing the improvement between groups, we employed a linear regression model. In this model:

①The dependent variable was the change in score from baseline to 12 months (Δ) for each primary outcome.

②The primary independent variable was the treatment group (intervention vs. control).

③We adjusted for the baseline score of the respective outcome as a key covariate. This approach precisely estimates the between-group difference in the magnitude of change while accounting for potential baseline imbalances, providing a robust and adjusted treatment effect.

2.Reported Results:The results of this multivariate analysis, which we have now integrated into the Results section (Pages 7-8, Lines 158-165), are as follows:

①For the FFP score: The adjusted between-group difference in the change score was -0.79 points (95% CI: -1.11 to -0.46; P < 0.001), favoring the intervention group.

②For the SPPB score: The adjusted between-group difference in the change score

was 2.02 points (95% CI: 1.04 to 2.99; P < 0.001), favoring the intervention group.
We believe that presenting these results from the multivariate regression analysis strengthens our manuscript by providing a clear, adjusted estimate of the intervention effect, directly addressing our research aim.

Line 385: check grammar, cardiac ward?

Response*: Thank you for your precise comment. We agree that 'cardiac ward' is a more appropriate term. We have revised the manuscript accordingly (Page 17, Line 405).*

Line 386: need to include the recruitment strategy

Response*: Thanks very much for this valuable suggestion to clarify the recruitment process. We have now added a detailed description of the recruitment strategy in the Methods section (Pages 17-18, Lines 403-420) of the manuscript.*
The revised text describes the following consecutive recruitment strategy, which was employed to minimize selection bias and ensure a representative sample:
（1）Identification: Potential participants were systematically identified through daily screening of all newly admitted elderly patients (aged ≥70 years) in the cardiac wards of the three participating tertiary hospitals.
（2）Screening: The medical records and clinical status of these patients were reviewed against the study's inclusion and exclusion criteria.
（3）Approach & Consent: Eligible patients and their families were approached on the ward. The study's purpose, procedures, risks, and benefits were explained in detail, and written informed consent was obtained.
（4）Baseline Assessment & Confirmation: Consented patients underwent a formal baseline assessment, including the FFP evaluation, to confirm they met all inclusion criteria (specifically an FFP score ≥3) before final enrollment and randomization.
We believe this revision provides a clear, transparent, and reproducible account of our recruitment process.

Line 387: The inclusion criteria need to be more specific.
Describe the cardiovascular health profile/prognosis of the target population.

Response*: Thank you for these important comments regarding the specificity of our inclusion criteria and the cardiovascular profile of our study population.*
1. Specificity of Inclusion Criteria:
We have revised the Methods section to provide greater operational detail for the inclusion and exclusion criteria, enhancing reproducibility.
（1）The central inclusion criterion of frailty is explicitly defined as a Fried Frailty Phenotype (FFP) score ≥3.
*（2） The exclusion criterion regarding "severe comorbidities" has been specified with objective examples, now reading: "*severe comorbidities including but not limited to metastatic cancer, end-stage renal disease on dialysis, severe chronic liver disease

(e.g., Child-Pugh class C cirrhosis), or any other condition with a clinical life expectancy of less than 12 months as judged by the attending physician." *(Pages 17-18, Lines 410-413).*

2. Cardiovascular Health Profile of the Target Population:

We agree that describing the cohort's cardiovascular profile is essential. As now emphasized in the Results section, our study enrolled a phenotypically-defined frail population from general cardiac wards, representing a broad spectrum of real-world cardiovascular conditions.

*（1）As detailed in **Table 1**, the participants' baseline characteristics confirm a high prevalence of major cardiovascular diseases, including hypertension (>84%), coronary artery disease (~71%), atrial fibrillation/flutter (~27%), and heart failure (~27%).*

（2）Key prognostic indicators, such as left ventricular ejection fraction (LVEF) and N-terminal pro-B-type natriuretic peptide (NT-proBNP) levels, were well-balanced between groups, further characterizing the cohort's cardiac status.

（3）Importantly, the exclusion of critically ill patients (e.g., those in CCU settings or with a life expectancy <12 months) defines a population with stable, yet significant, chronic cardiovascular disease, for whom strategies to manage frailty are highly relevant.

This approach-using a phenotypic frailty criterion within a broadly defined cardiac inpatient population-ensures the clinical generalizability of our findings to the common scenario of managing frailty across various cardiovascular diagnoses.

Line 390: what do you mean by this? please define

***Response**: Thanks for pointing out the need for a clearer definition. We agree that the term "severe comorbidities" was too vague. This criterion was intended to identify patients whose underlying non-cardiovascular conditions would likely limit their life expectancy to less than 12 months or prevent safe participation in the 12-month intervention.*

*We have revised this exclusion criterion in the Methods section for greater precision and reproducibility. It now reads:"*The exclusion criteria were: ... (2) cognitive impairment or severe comorbidities including but not limited to metastatic cancer, end-stage renal disease on dialysis, severe chronic liver disease (e.g., Child-Pugh class C cirrhosis), or any other condition with a clinical life expectancy of less than 12 months as judged by the attending physician; ..." *(Pages 17-18, Lines 408-413).*

Line 404: more elaboration on this is required

***Response:** Thank you for this important comment. We agree that elaborating on the individual assessments is crucial for understanding the personalization of the RENAP intervention. We have now added a description in the manuscript as "*The intervention

cohort participated in a year-long, personalized multidisciplinary program (RENAP) based on the problems identified by CGA. CGA included functional capacity assessment, nutritional status assessment, medication review, insomnia assessment, and TCM constitution and symptom assessment." *(Page 18, Lines 430-432) and a more detailed elaboration is provided in the supplementary materials.*

The assessments primarily comprised these components:

1. Functional Capacity Assessment: The Short Physical Performance Battery (SPPB), physical fitness tests, and Fried Frailty Phenotype (FFP) were used to quantify the degree of physical frailty and functional limitation. The physical fitness tests included a 30 second sit-to-stand test to assess lower body strength, a 30-second arm curl test to assess upper body strength, a 2 minutes step test to assess the cardiopulmonary function and aerobic endurance, a chair sit-and-reach test to assess lower body flexibility, a back scratch test to assess upper body flexibility, and a time-up-and-go test (TUG) to evaluate dynamic balance and agility. This directly informed the intensity and type of rehabilitation exercises prescribed.

2. Nutritional Status Assessment: Age, grip strength, daily physical activity, NRS 2002 score, clinical diagnosis (especially sarcopenia), labora tory results, human body composition analysis (Inbody 720), and dietary survey were administered to identify malnutrition risk. This guided the specific focus and recommendations within the nutritional guidance component.

3. Medication Review: Medication appropriateness index (MAI), review of the type, measurement and quantity of drugs used by patients during hospitalization were evaluated by a clinical pharmacist. This formed the basis for the polypharmacy management intervention.

4. TCM Constitution & Symptom Assessment: For the acupoint massage component, TCM physicians will comprehensively and systematically assess the participants based on the TCM theory of syndrome differentiation, including a TCM tongue diagnosis and pulse diagnosis.

Based on the results of this multidimensional assessment, an individualized care plan was created for each participant, specifying tailored goals and the specific interventions within each of the five RENAP domains. We believe this elaboration provides a clear and reproducible account of how the personalization of the intervention was achieved.

Line 412: exercise duration per session

***Response**: Thank you for this constructive feedback. We agree that specifying the exercise duration per session is essential. We have now revised the manuscript to provide a more detailed description of the exercise protocol, including the duration, structure, and progression principles.*

The revised text in the manuscript now reads as follows:"Participants performed 3 to

20 repetitions for each exercise and three to five times weekly for 12 months. The frequency, time and feelings concerning exercise each week were recorded. The exercise intensity was progressively adjusted by the supervising rehabilitation physicians. The progression was guided by the patient's subjective tolerance and clinical observation for any adverse effects. The intensity remained at a 'safe level,' which was defined as the highest level not associated with pain, adverse symptoms, or functional deterioration. Daily walking was also encouraged." *(Page 19, Lines 439-447).*

We believe this elaboration provides the necessary clarity and ensures the reproducibility of our exercise intervention.

Line 421: how was this translated into layman term for the patients?

***Response**: Thank you for this practical and important question. We agree that translating nutritional science into actionable advice for patients is crucial for adherence. The recommendation for "high-quality protein sources such as whey and casein" was communicated to patients in the following layman's terms and practical strategies:*

-Simple Analogies and Concepts: We explained to patients that "just like building a house needs high-quality bricks, your body needs high-quality protein to build and maintain muscle strength, especially for recovery and to prevent frailty." We described whey and casein as "premium, fast-absorbing protein" found in milk, ideal for recovery.

-Focus on Everyday Foods: The advice was translated into a focus on consuming specific, accessible foods. Patients were encouraged to:

Increase dairy intake: "Please try to include milk, yogurt, or cheese in your meals. For example, drink a glass of milk with breakfast and have a cup of yogurt as a snack."

Use milk in cooking: "You can use milk instead of water to make oatmeal or soups to add more protein easily."

Concrete Action Plan: For patients with poor appetite or difficulty meeting protein needs through food alone, we provided very specific instructions, such as: "If you can't eat much at mealtimes, consider having a nutritional drink supplement, like Ensure or a similar product, once or twice a day. These are convenient sources of the high-quality protein we discussed."

This patient-centered communication strategy was a key component of the nutritional guidance intervention to ensure comprehension and practical implementation.

Line 423: what is the % of total energy intake from protein?

***Response**: Thank you for raising this important point. In our study, the protein*

recommendation was primarily prescribed as an absolute intake of 1.0-1.5 grams per kilogram of body weight per day, consistent with guidelines for muscle health in the elderly.

*Based on the estimated total energy intake for our participant profile, this absolute protein intake corresponds to approximately 15%-20% of total energy intake. We have clarified this in the revised manuscript by adding the following sentence:"*protein intake corresponded to approximately 15%-20% of the total energy intake,*" (Page 19, Lines 458-459).*

This provides both the practical prescription and the complete energy distribution you rightly requested.

Line 425-427: more elaboration is required. Including relevant references are helpful.

***Response**: Thanks for this valuable suggestion. We agree that a more detailed description of the TCM assessment methodology would enhance the clarity and scientific rigor of our manuscript. We have thoroughly revised the corresponding section in the manuscript to provide a more elaborate explanation of the TCM syndrome differentiation process, including tongue and pulse diagnoses. Furthermore, we have cited relevant and authoritative references to support our methodological approach.*

*The revised text now reads:"*TCM physicians assessed the participants based on the TCM theory of syndrome differentiation, including a TCM tongue diagnosis and pulse diagnosis.[31] Tongue diagnosis provides visual information about the patient's health status by examining the tongue's color, shape, and texture.[32] Pulse diagnosis assesses the quality, rhythm, and strength of the pulse to evaluate the functional status of internal organs.[33] The examinations, combined with other clinical symptoms and signs, was integrated to determine the TCM syndrome of each participant. Then TCM practitioners guided participants in self-administered acupoint massage." *(Pages 19-20, Lines 461-469; Refs 31-33).*

We believe these modifications have significantly improved the manuscript, and thank you again for the constructive comment.

Line 428-429: some explanation for these terms would be helpful for the readers.

***Response**: Thank you for this valuable suggestion to enhance the clarity of our manuscript for a broader readership. We agree that some explanation of the acupoint terms is necessary.*

*We have now revised the manuscript to include a brief, accessible explanation of the purpose of these acupoints directly in the main text, while referring the readers to the supplementary materials for the detailed methodological description of location and massage technique. The revised text in the manuscript now reads:"*For constipation, points included Zhigou, Zusanli, and Dachangshu were selected in regulating intestinal function. For insomnia, points included Neiguan, Shenmen and Sanyinjiao were selected in calming the mind and improving sleep quality. A detailed description of acupoint localization and massage procedure is provided in the Supplementary Materials (Appendix 1 and 2). *" (Page 20, Lines 469-474).*

We believe this approach strikes an appropriate balance between maintaining the flow of the main text and providing comprehensive, reproducible details for interested readers.

Line 430: by who? where was this conducted?

***Response**: Thank you for this question, which allows us to clarify the implementation of the acupoint massage intervention.*

The sessions referred to in the sentence were self-administered by the participants themselves at home, following the initial training and guidance provided by the TCM practitioners. This was a core component of the intervention designed to empower patients and promote adherence in a real-world setting.

*We have revised the manuscript to make this point explicitly clear and avoid any ambiguity for the reader. The revised text now reads:"*Following guidance from TCM practitioners, participants were instructed to perform self-administered acupoint massage at home. Each self-administered session lasted 5-10 minutes and was performed 1-2 times daily.*" (Page 20, Lines 474-476). We hope this clarification addresses your question.*

Line 435: Ambiguous. More detail description of the process of consultation is require, e.g. who were the physicians? How did the intervention pharmacist approach the treatment requirement and patients' preference?

***Response**: Thank you for your valuable feedback. We have revised the manuscript to clarify the consultation process, which now reads "*Clinical pharmacists assessed the appropriateness, efficacy, and safety of participants' medications. They then collaborated with the patient's attending physician to develop a consensus-based treatment plan. This plan was discussed with the participant in a shared decision-making process before implementation. Medication use was re-evaluated at follow-ups, and this collaborative process was repeated to adjust treatments as needed. Detailed procedures are provided in the Supplementary Materials.*" (Page 20, Lines 477-483). The text now explicitly states that clinical pharmacists collaborated with*

the patient's attending physicians through a process of structured review, interdisciplinary consensus-building, and shared decision-making with participants. We have also added a reference to the Supplementary Materials, which contain the comprehensive procedure.

We believe these revisions eliminate the ambiguity and appreciate your suggestion.

Line 454: what was the usual care?

***Response**: Thank you for this important question. The usual care included standard medical management by the ward team and routine outpatient clinic visits as per standard practice. No structured, multidisciplinary intervention targeting frailty was provided. All participants, including the control group, underwent the same schedule of research assessments.*

We have revised the manuscript to specify the components of "usual care" received by the control group. The revised text now reads:"<u>Participants in the control group received standard medical management, routine monitoring, and general advice on diet and lifestyle. After discharge, they received the standard follow-up provided by the hospital. No structured, multidisciplinary intervention targeting frailty was provided. All participants, including the control group, underwent the same schedule of research assessments (e.g., FFP, SPPB) at baseline, three, six, and twelve months.</u>*" (Page 21, Lines 502-507).*

We believe this clarification accurately defines the control condition and strengthens the validity of our comparative analysis.

Line 459-460: define each of these criteria.

***Response:** Thank you for this suggestion. We agree that defining the operational criteria for the Fried Frailty Phenotype is essential for clarity and reproducibility.*

Frailty was assessed using the Fried Frailty Phenotype (FFP), which is based on five predefined criteria from [Fried LP, Tangen CM, Walston J, et al. Frailty in older adults: evidence for a phenotype. J Gerontol A Biol Sci Med Sci. 2001 Mar;56(3):M146-56. doi: 10.1093/gerona/56.3.m146.]*:*
1. Unintentional weight loss: Self-reported unintentional loss of >5% of body weight in the past year.
2. Exhaustion: Identified by two statements from the Center for Epidemiologic Studies Depression Scale (CES-D). Participants answering "a moderate amount of time (3-4 days)" or "most of the time" to the question "I felt that everything I did was an effort" or "I could not get going" were categorized as meeting this criterion.
3. Weakness: Assessed by grip strength, measured with a handheld dynamometer. The lowest 20% of the cohort (stratified by gender and body mass index) were categorized

as weak.
4. Slow walking speed: Measured by the time taken to walk 4.57 meters (15 feet). The slowest 20% of the cohort (stratified by gender and height) were categorized as having slow walking speed.
5. Low physical activity: Evaluated using the short version of the International Physical Activity Questionnaire (IPAQ). The lowest 20% of the cohort (stratified by gender) were categorized as having low physical activity.
A participant was defined as frail if they met three or more of these criteria.

*We have revised the manuscript as "*The FFP score, providing a holistic, syndromic classification of an individual's overall vulnerability, assesses frailty through criteria: unintentional weight loss >5% of body weight in the past year, self-reported exhaustion, weakness (low grip strength), slow walking speed, and low physical activity, with frailty defined by meeting three or more criteria.[2]*" (Pages 21-22, Lines 509-513) in the Methods section.*

We believe this provides the necessary methodological detail and thank you for the suggestion to improve our manuscript.

Line 461: what was the protocol for SPPB, was a standardize procedure used or was it developed by the team?

Response: *Thank you for this question. We confirm that the Short Physical Performance Battery (SPPB) was administered using the standardized, well-established protocol originally developed by the National Institute on Aging (NIA) and widely used in geriatric research.*

The assessment was not developed or modified by our research team. It was conducted exactly as per the standard procedure, which includes three components:
-Balance tests: Measuring the ability to hold three standing positions (side-by-side, semi-tandem, and tandem) for 10 seconds each.
-Gait speed test: Timing the participant as they walk 4 meters at their usual pace.
-Chair stand test: Recording the time required to stand up from a seated position five times, as quickly as possible.
Scores from each component (ranging from 0 to 4) were summed to create a total SPPB score between 0 and 12.

*We have now clarified this in the manuscript by adding the sentence: "*The SPPB was administered according to the standardized protocol developed by the National Institute on Aging.*" (Page 22, Lines 516-517). We appreciate this opportunity to enhance the methodological clarity of our work.*

Line 463: spell out acronym when first use

Response: *Thank you for your careful reading of our manuscript. The acronym "CFS" for the Clinical Frailty Scale was indeed defined upon its first introduction in the Results section on page 7 (Line 146). We hope this further clarification meets your approval.*

Line 468: how was this defined?

Response: *Thank you for this important question regarding the definition of our endpoint. We agree that a precise definition is crucial.*

In our study, "non-elective hospital readmission" was defined objectively as an unplanned hospital admission that occurred through the Emergency Department or required direct hospitalization from an outpatient visit, regardless of the primary diagnosis. This definition excludes pre-scheduled admissions for elective procedures or routine care. To ensure consistency and reproducibility, this outcome was ascertained by two independent researchers.

We have revised the manuscript to include this explicit definition: "<u>...non-elective hospital readmission at one year, which was defined as any unplanned admission via the Emergency Department or following an clinical visit</u>*." (Page 22, Lines 521-523).*

We believe this clarification strengthens the objectivity of our secondary outcome measure.

Line 473: ambiguous

Response: *Thank you for this critical comment. We agree that the description of the sample size calculation was ambiguous and have revised it to provide full methodological transparency.*

The sample size was calculated using a two-sided, two-sample t-test for comparing means between the intervention and control groups based on the following parameters:
Statistical power (1-β): 80%
type I error (α): 0.05 (two-sided)
Allocation ratio: 1:1
Anticipated dropout rate: 15%-20%

Ian D Cameron et al. studied frail patients using the reduction in FFP score from baseline after multidisciplinary intervention as the primary endpoint. Their results showed:
  - Intervention group: mean=0.8, SD=1.19
  - Control group: mean=0.41, SD=1.02
- When using SPPB score as the primary endpoint, their results were:

- *Intervention group: mean=5.83, SD=2.82*
- *Control group: mean=4.69, SD=2.91*

Tze Pin Ng et al. studied pre-frail and frail patients with post-intervention Fried score as the primary endpoint, reporting:

- *Intervention group: mean=1.2, SD=1.07*
- *Control group: mean=1.6, SD=0.97*

Based on this calculation, a minimum of 160 participants per group was required and the total target sample size was inflated to 320 participants.

*We have revised the manuscript as follows: "*The sample size was calculated based on the mean comparison between two groups using two-sided, two-sample *t*-test (power = 80%, α = 0.05). *" (Page 22, Lines 528-529).*

Line 474-475: please check, becoming more frail?

Response: *Thank you for this exceptionally astute observation. We sincerely appreciate you pointing out this ambiguity in our wording, which could indeed be misinterpreted.*

*The intended meaning was not that the intervention would cause an increase in frailty. To eliminate all confusion, we have rephrased the sentence in our manuscript as "*to detect a 33% between-group difference in the mean number of frailty criteria*" (Page 22, Lines 530-531). We apologize for the lack of clarity in our original statement and thank you again for helping us improve the precision of our manuscript.*

Line 508: ?

Response: *Thank you for prompting us to clarify this critical endpoint. The outcome of "death" (all-cause mortality) was defined as death from any cause, occurring at any time after randomization and within the one-year follow-up period.*

The ascertainment of death was conducted through a rigorous, multi-source verification process:

-*Primary Source: Review of hospital medical records and death certificates.*

-*Secondary Source: Follow-up phone calls with the participant's designated family members or caregivers.*

All reported death events were centrally adjudicated by two independent investigators blinded to the group allocation, to ensure accuracy and objectivity.

*We have revised the Methods section of the manuscript (Page 24, Line 565) as "*all-cause mortality*" to include this explicit definition and ascertainment process, ensuring full reproducibility.*

Line 512-513: This is confusing. More clarity is desired. How are these different

to the primary outcomes?

Response: *Thank you for this important comment. We apologize for the confusion caused by our initial vague description. We have now revised the manuscript to provide a clear and precise explanation of our subgroup analyses.*

The subgroup analyses were performed for the primary outcomes (specifically, the changes in FFP and SPPB scores at 12 months) to explore whether the treatment effect was consistent across different patient characteristics at baseline. These are not different outcomes but the same primary outcomes analyzed within predefined patient subgroups. The subgroups were defined by the following baseline characteristics:
Age (<80 years, ≥80 years)
Sex (Male, Female)
Body Mass Index (BMI) (≤24, >24)
Presence of Heart Failure (Yes, No)
Presence of Coronary Artery Disease (Yes, No)
Presence of Atrial Fibrillation/Flutter (Yes, No)
Presence of Diabetes (Yes, No)

The revised text in the Methods section now reads: "The pre-specified subgroups were chosen based on a priori clinical relevance rather than post-hoc data exploration to examine the consistency of the intervention's effects across different demographic and clinical subgroups, including sex, age (≥80, <80 years), BMI (BMI >24, 18 ≤ BMI ≤ 24), heart failure, coronary artery disease, atrial fibrillation/flutter (AF/Afl), and diabetes. Pre-specified subgroup analyses were conducted for the primary outcomes (FFP and SPPB score changes) using the same statistical model as the primary analysis." *(Page 24, Lines 569-574). We believe this revision eliminates the ambiguity and accurately reflects our statistical plan.*

Reviewer #2:

Chai et al. report the results of a year-long followup of a multi-modal intervention using education, diet, physical therapy, and traditional Chinese medicine with control of polypharmacy for treatment of frailty in patients with cardiovascular diseases (CVD). As the authors make clear, their approach is predicated on previous work, primarily in outpatient settings, to treat sarcopenia and frailty. A notable point about this work is recruitment during inpatient hospitalizations for CVD, which led to a more severely ill cohort of patients with CVD specifically. The application of culturally appropriate interventions to the Chinese population is also a novel point.

The analysis seems to have been done appropriately and the results suggest that there was clinically relevant and statistically significant improvement in frailty (using the Fried criteria), functon(SPPB and IADLs), and hospitalizations. There was a numeric trend on mortality but it was not significant, probably due to small numbers of events in the two groups. Limitations are clearly described.

My only concern is that the effect seemed to wear off between 6 and 12 months of followup, and this is not addressed in the discussion (see Figure 3). Both groups had strong improvements after hospital discharge, consistent with expectations that going home from the hospital is a good idea and that most people recover from the acute illness to some extent by 3 months. Both groups also maintained that improvement to 6 months in terms of FFP, and both had further improvement in SPPB to 6 months. It is in the next 6 months that the groups diverge, with worsening results between 6 and 12 months that were more notable in the control group than the actively treated group.

A true skeptic could say that there was no benefit to RENAP after 6 months, but a more likely interpretation is that the general improvement caused by recovering from the hospitalization (and reaping the benefits of the inpatient treatment) swamped the effect of the RENAP intervention until after month 6; only once that post-hospitalization effect receded were the effects visible.

Although the investigators "adjusted the interventions (line 449)" during the 3- and 6-month visits in the active group, one wonders whether a more comprehensive refresher treatment with the RENAP protocol at month 6 would have had a larger protective effect at 1 year. This is a key point, since knowing how often to intervene is as important as knowing how to intervene to treat or prevent frailty. This would take a follow up study to ascertain, but at least it should be discussed.

The effect of adherence is also worth additional comment in the discussion. On comparing Fig 3 with Suppl Fig 1, we see a roughly 20% higher difference in

FFP (difference between groups at 12 months = 0.8 vs. 0.97, ITT vs PPS) and a larger 45% difference in SPPB (difference 1.84 in ITT vs. 2.68 in PPS). This should be more clearly explained in the Results section, including clearly stating that the primary results are from the ITT population (lines 151ff), and directly comparing the sample sizes and effect sizes in ITT vs. PPS.

RESPONSE:

We sincerely thank you for the thoughtful, positive, and constructive feedback on our manuscript. We are particularly grateful for the insightful interpretation of the longitudinal results and the specific suggestions to strengthen the discussion. We have revised the manuscript accordingly to address these points, as detailed below.

Comment 1: The primary concern regarding the wearing off of the effect between 6 and 12 months and the lack of discussion on this point.

Response*: We agree that the divergence in trajectories between 6 and 12 months is a critical finding that warrants detailed discussion. We appreciate your plausible interpretation that the strong post-hospitalization recovery may have initially overshadowed the RENAP effect, which became more visible as this natural recovery plateaued.*

*We have now added a dedicated paragraph in the Discussion section to address this directly. "*Our longitudinal analysis revealed a time-dependent treatment effect, which offers crucial insight into the intervention's mechanism of action. The parallel, modest improvements in frailty (FFP) and comparable declines in physical function (SPPB) observed in both groups during the first 6 months suggest that the natural recovery process following hospital discharge was the dominant influence, potentially obscuring the specific effect of the RENAP program in the short term. The critical divergence emerged between 6 and 12 months. The control group experienced a rebound in frailty and stagnation in physical function, a pattern indicative of the natural, unsupported course for these frail patients. In stark contrast, the RENAP group not only maintained its frailty improvements but also achieved significant functional recovery. This pattern underscores that the principal value of our sustained, multi-component intervention lies not in accelerating immediate post-discharge recovery, but in its long-term efficacy to prevent the recurrence of frailty and proactively facilitate functional gains, thereby altering the detrimental trajectory of this high-risk population.*" (Page 13, Lines 291-305).*

Comment 2: The suggestion to discuss the potential impact of a more comprehensive refresher at month 6.

Response*: Thanks very much for this excellent suggestion. We have incorporated this*

directly into the new discussion paragraph (as shown above).

Comment 3: The need for a clearer explanation of the adherence effect by comparing ITT and PPS analyses in the Results section.

***Response**: Thanks for this important clarification. We have now revised the Results section to explicitly state that the primary analysis is based on the Intention-to-Treat (ITT) population and have added a direct comparison of the effect sizes between the ITT and Per-Protocol Set (PPS) analyses, as requested.*
Revised text in the Results section now reads as: "In the Intention-to-Treat (ITT) analysis, at the 12-month follow-up, the intervention group showed significant improvements in both FFP and SPPB scores compared with baseline (Table 2). The mean FFP score decreased by 1.8 points in the intervention group compared to a decrease of 1.1 points in the control group, resulting in a between-group mean difference of -0.79 points (95% CI: -1.11 to -0.46; $P < 0.001$). Additionally, the mean SPPB score improved by 0.4 points in the intervention group, while it decreased by 1.6 points in the control group, leading to a between-group mean difference of 2.02 points (95% CI: 1.04 to 2.99; $P < 0.001$). Figure 2A and 2B summarizes the changes in FFP and SPPB scores over the study period. In the Per-Protocol Set (PPS) analysis, which included participants completing the intervention and having outcome assessments (n=120 for RENAP, n=132 for control), the intervention group showed significant improvements in both FFP and SPPB scores (Supplemental Table 4, Supplemental Figure 1A and 1B). The between-group difference in FFP and SPPB at 12 months was 0.90 (95% CI: -1.27 to -0.52; $P < 0.001$) and 2.78 (95% CI: 1.65 to 3.90; $P < 0.001$) in the PPS analysis, respectively." *(Pages 7-8, Lines 158-172).*

Once again, we extend our deepest gratitude for the time and invaluable comments, which have significantly improved the depth and clarity of our manuscript.

Reviewer #3:

Dear authors,

This is a very interesting study and paper on very timely topics and growing concerns for healthcare and society (frailty, CVD, and outcomes). Having read the response to previous reviews, I also commend the authors for their very frank and thoughtful responses. I do believe this paper can be of great interest to a broad range of readers, but I also have a number of concerns and queries, as below:

Introduction:

Please add a citation for lines 91, 99, 108.

Response: *Thanks for your kind attention to this issue. We have added the appropriate citations in the revised manuscript (Page 5, Lines 91, 99, and 108). The relevant references (Refs 1, 4, and 6) have been included in the reference list accordingly.*

Line 116: Why is this acronym RENAP, or what does RENAP stand for? Please write it out fully first, then use the abbreviation, and cite it if any publication for it already exists.

Response: *Thanks for your kind attention to this issue. This comprehensive multidisciplinary program included Rehabilitation exercises, patient Education, Nutritional guidance, Acupoint massage, and Polypharmacy management. Using the acronyms of these intervention measures, we abbreviated the entire set of intervention measures as RENAP. We have made further explanations in the manuscript. The revised text in the Introduction section now reads: "*Building on established interventions and our clinical experience, we developed a comprehensive, in-hospital initiated, multidisciplinary program—RENAP (Rehabilitation, Education, Nutritional guidance, Acupoint massage, and Polypharmacy management).*" (Page 6, Lines 121-124). Thank you again for your reminding.*

Results:
Lines 144-145: I commend the authors for including the PSQI. Sleep is a major contributor to physical and mental health and well-being and poor raises the risk and increases the severity of a number of health conditions associated with frailty and CVD.

Response: *Thanks for this positive feedback and for highlighting the critical importance of sleep health in the context of frailty and cardiovascular disease. We fully agree with the assessment. The inclusion of the Pittsburgh Sleep Quality Index*

(PSQI) was intentional, as we recognize that sleep disturbances represent a key, yet often under-assessed, domain in comprehensive geriatric care. We are pleased that you acknowledges this aspect of our study design.

Line 146: Was the loss to follow-up (LTF) evenly distributed between groups? What was the cause of the LTF?

Response: *We appreciate your insightful comment on loss to follow-up (LTF). The overall attrition was 81 patients (46 in the intervention group vs. 35 in the control group). The final completion rates (72.3% intervention vs. 79.0% control) were not statistically significantly different, as presented in the manuscript. A detailed breakdown of the LTF reasons is as follows:*
(1) Withdrawal of informed consent: 25 participants (16 in the intervention group, 9 in the control group);
(2) Unable to contact: 29 participants (17 in the intervention group, 12 in the control group);
(3) Early study termination: 4 participants (2 in each group);
(4) Missing primary outcome data due to reasons such as seeking medical care at other hospitals and inability to attend follow-up visits: 23 participants (11 in the intervention group, 12 in the control group).
These data are also summarized in Figure 1 of the manuscript. The distribution of these causes was relatively balanced between the intervention and control groups.

To assess the impact of LTF and evaluate whether it was evenly distributed between the two groups, we performed a two-tiered comparison:
Comparison 1: All Completers vs. All LTF
This analysis aimed to determine whether the group of non-completers itself differed systematically from those who completed the study. The results, presented in the supplementary Table 2, indicate that the 252 patients who completed the study and the 81 patients LTF showed almost no significant differences in most key baseline characteristics (e.g., age, sex, major comorbidities, etc.).

Comparison 2: LTF in the Intervention Group vs. LTF in the Control Group
This analysis was conducted to assess whether the participants lost to follow-up were comparable between the two study arms. As shown in the supplementary Table 3, a comparison of the 46 patients LTF in the intervention group and 35 in the control group revealed nearly no notable differences in their baseline characteristics including age, sex, major comorbidities, and baseline FFP and SPPB scores, except diabetes mellitus. This indicates that the risk of bias due to uneven attrition between the groups is likely low. Regarding the baseline imbalance in diabetes history between groups, clinical judgment suggests its impact on the primary study endpoints is likely limited.

Meanwhile, For missing data in the Intention-to-Treat (ITT) analysis, single

imputation using the Expectation-Maximization algorithm was applied, followed by analysis with a mixed-effects model. For the Per Protocol Set (PPS), no imputation was performed. Regarding time-to-event outcomes, participants who did not experience the event within the one-year observation period were right-censored. The conclusions were consistent between the PPS and ITT analyses.

We fully acknowledge your concern regarding the potential impact of LTF on study outcomes. While our comparative analysis indicated that LTF was relatively balanced between the two groups, we completely agree that any attrition could introduce bias. This limitation has been explicitly acknowledged and discussed in the manuscript as "<u>Third, participants' adherence and missing data have a significant impact on the research outcomes.</u>*" (Page 15, Lines 356-357).*

Line 187: This is likely true but that statement is not a result, it is an conjecture on the result, and that belongs in the discussion section, not the results.

Response: *Thanks for your kind attention to this issue. We couldn't agree more with you and we apologize for the inaccuracy. This sentence has been removed from the results section of the manuscript.*

Discussion:

Line 204: I suggest function, rather than "functionality."

Response: *Thanks for your reminder. It has been changed in the manuscript (Page 9, Line 211).*

Line 208: This sentence could use some editing. The beginning is a bit unclear: "Our study show integrating frailty assessments with targeted interventions may lead to significant improvements in both clinical and functional outcomes, addressing the growing challenges of CVD and frailty on a global scale." Does this mean that frailty assessments paired with targeted interventions can lead to the improvements?

Response: *Thank you for attention. After your reminder, we realized that the expression of this sentence was indeed unclear. At the same time, we found that there was repetition with the context. Therefore, we deleted this sentence to make the paragraph expression clearer and more concise. Thank you again for your correction.*

Please add a citation for line 214

Response: *Thanks for your kind attention to this issue. We have added the appropriate citations in the revised manuscript (Page 10, Line 217; Refs 12 and 13).*

Line 219: Are all of these targeting insomnia? Does this population have a high prevalence of insomnia, or Insomnia Disorder? This should be mentioned as well in the introduction so that it is more sensible here. Otherwise, this comes as a surprise (insomnia). Same for constipation. How is this related to frailty or CVD? Are the RENAP intervention components all targeting insomnia and constipation?

Response: *Thanks for your advice. We fully agree with you. Sleep disturbances and constipation are highly prevalent among frail elderly patients with CVD, contribute significantly to reduced quality of life, and can exacerbate the frailty cycle. Acupoint massage, derived from traditional Chinese medicine (TCM), is recognized as an effective complementary therapy for insomnia symptoms and constipation, which contributes to a higher quality of life for these patients. In response, this sentence has been added in the Introduction section (Page 6, Lines 119-121) in the revised manuscript. This addition provides the necessary justification for including a component specifically aimed at these issues, making the subsequent mention of our intervention components far more logical and less surprising.*

We sincerely apologize for the ambiguity caused by language expression in our previous wording. The RENAP program is a multicomponent intervention designed to address the multifaceted nature of frailty in elderly CVD patients. Among these intervention measures, only acupoint massage was aimed at insomnia and constipation, while other components target broader aspects of health, including medication adherence, malnutrition, physical deconditioning, and overall self-management, which are central to frailty and CVD management. We have revised the order of expression of the sentence as "<u>including acupoint massage to address insomnia and constipation, patient education, and nutritional guidance</u>*" (Page 10, Lines 221-222).*

We hope these revisions have eliminated the confusion and provided appropriate background for the inclusion of acupoint massage in our intervention bundle.

Line 221- this leaps to anxiety levels. But what was the result in terms of insomnia and constipation? How did you measure constipation in the sample?

Response: *Thank you for raising this critical point, which highlights a limitation in our initial analysis and reporting.*

In this study, constipation was not assessed using a validated scale. It was documented as a patient-reported outcome based on spontaneous reporting of symptoms. Therefore, we lack quantitative data to formally compare constipation rates between the groups.

We acknowledge that our original claim was not fully supported by the presented evidence regarding the specific symptoms of insomnia and constipation. We have toned down the conclusion in the manuscript to focus on the outcomes we can robustly demonstrate. The sentence now reads: "<u>These added intervention items were designed</u>

to address a broader range of geriatric issues." *(Page 10, Lines 222-223).*

We have also added a sentence in the Discussion-Limitations section to acknowledge this: "Furthermore, while interventions like acupoint massage were included to address insomnia and constipation, the lack of systematic, quantitative assessment of these specific outcomes limits our ability to draw definitive conclusions about their efficacy." *(Page 16, Lines 375-378).*

Thanks for this suggestion, which has improved the precision of our manuscript.

Line 227: acupoint massage is never introduced in the intro- what is the background for it. Why did you add this as an intervention?

Response: *Thanks for your attention. As a simple and easy-to-implement intervention suitable for China's current diagnostic and treatment landscape, traditional Chinese acupoint massage has been proven to improve patients' sleep and constipation, and may improve frailty. Our prior clinical practice and preliminary experiments indicate that acupressure is beneficial in alleviating patient frail condition. Based on existing literature and intervention strategies, combined with our clinical experience, we have incorporated traditional Chinese acupoint massage into our intervention methods. We have added this in the introduction section in the Introduction section of the manuscript (Page 6, Lines 119-124).*

Line 233: I don't see how this relates to your study as your participants had frailly. I see the connection in terms of CVD, but the issue is frailty as well.

Response: *Thank you very much for this insightful comment, which has helped us clarify the logical connection between our study and the SPRINTT trial. We can't agree more that frailty is the core condition our study aims to address. The SPRINTT project specifically enrolled community-dwelling older adults with physical frailty and sarcopenia. Our revised text now explicitly highlights this fundamental common ground.*
The sentence in the Discussion has been amended to read:"The SPRINTT project by Bernabei et al. demonstrated the efficacy of physical activity and nutritional counseling in reducing mobility disability in older adults with physical frailty and sarcopenia" *(Page 10, Lines 226-228).*
This revision serves to emphasize the shared frailty, thereby justifying the comparison. Position our work logically as an extension of the SPRINTT findings, demonstrating how we build upon evidence-based components (physical activity and nutrition) by integrating them with additional modalities (e.g., acupoint massage, polypharmacy management) and applying them in a novel clinical context (the inpatient CVD setting). We believe this clarification strengthens the logical flow of our argument by making it clear that we are intervening on the same core condition while introducing novel elements for a distinct patient population and care setting.

Line 236: did they investigate this in persons with frailty? If not, this really doesn’t add anything and doesn’t need to be added. If so, say that they investigated this in older adults with frailty.

Response: *Thank you for this correct and insightful observation. Upon re-examination, the study by Eichler et al. did not specifically enroll a cohort defined by frailty but rather assessed frailty as one of several metrics in a general TAVR population undergoing rehabilitation. Therefore, as you suggested, the comparison does not add value and could be misleading. We have removed this sentence from the manuscript.*

Line 252: here you could add the line about future studies using a larger sample size to investigate this.

Response: *Thanks for your thoughtful reminder. It has been added in the manuscript (Page 12, Line 270).*

Line 255, line 257, line 261 (susceptibility), line 263 : these definitely need citations. I don’t dispute these statements, but whenever you state something as a fact, it needs to be supported with an appropriate citation.

Response: *Thanks for this feedback. We agree that these foundational statements should be supported by citations. We have added the appropriate citations in the revised manuscript. The relevant references have been included in the reference list accordingly.*

Line 255 (Page 12, Line 265 in the revised manuscript): Ref 19 [Welch Carly,Chen Yaohua,Hartley Peter,et al.New horizons in hospital-associated deconditioning: a global condition of body and mind.AGE AND AGEING.2024;53 (11).doi:10.1093/ageing/afae241].

Line 257 (Page 12, Line 267 in the revised manuscript): Ref 3 [Hoogendijk Emiel O, Afilalo Jonathan,Ensrud Kristine E,et al. Frailty: implications for clinical practice and public health. Lancet. 2019; 394 (10206): 1365-1375. doi: 10.1016/S0140-6736(19)31786-6].

Line 261 (Page 12, Line 274 in the revised manuscript): Ref 20 [Dapp Ulrike,Minder Christoph E,Golgert Stefan,et al.The inter-relationship between depressed mood, functional decline and disability over a 10-year observational period within the Longitudinal Urban Cohort Ageing Study (LUCAS).JOURNAL OF EPIDEMIOLOGY AND COMMUNITY HEALTH.2021;75 (5) ： 450-457.doi:10.1136/jech-2020-214168].

Line 263 (Page 12, Line 276 in the manuscript): Ref 21 [Davoodian Najmeh,Forbes Malcolm,Berk Michael,et al.Contribution of depression and cardiometabolic diseases and the role of depression treatment in survival and functioning in older adults.EClinicalMedicine.2025;82：103182.doi:10.1016/j.eclinm.2025.103182].

Line 270: Please explain a bit how this is culturally adaptable.

Response: *Thanks for your advice. What we meant originally was that it was more suitable and adaptable for Chinese people. To avoid misunderstanding, we have removed relevant expressions in the revised manuscript (Page 13, Line 287).*

Line 274: perhaps your research contributes, rather than demonstrates?

Response: *Thanks for this thoughtful suggestion. We agree that the wording could be more precise. To strengthen the manuscript and avoid repetition, we have chosen to remove the entire sentence in question. We believe this decision streamlines the narrative and addresses the comment effectively.*

Line 281: Regular exercise also improves sleep, which increases immune and metabolic function, organ repair and body tissue repair, cognition, mood, and anxiety. There is also a bidirectional relationship between sleep and exercise. These would be important to mention (and cite) as well. Exercise also improves cognition and mood/anxiety independently of sleep and nutrition.

Response: *Thank you very much for this suggestion to elaborate on the pleiotropic benefits of exercise. After careful consideration, we determined that while these points are scientifically valid, expanding on them in this specific paragraph would shift the focus away from the primary thread of our discussion, which is the integrated effect of the RENAP bundle.*

To maintain a concise and focused narrative that directly serves our study's primary objectives, we have opted to remove the broader sentence on the systemic benefits of exercise. We believe this decision strengthens the manuscript by ensuring that every element of the Discussion is directly aligned with the interpretation of our results and the core message of our multidisciplinary intervention.

We have, however, ensured that the fundamental role of exercise within our intervention is clearly articulated in the Introduction and Methods sections.

Line 287: This must be cited. It needs to be supported by citations. Also, are you referring to insomnia symptoms, or insomnia disorder (difficulty falling or staying asleep ≥3 times per week for >3 months with daytime impairment)? They are different.

Response: *Thank you for this critical and insightful comment. We fully agree that the statement required both citation support and conceptual precision.*

We have added citations to robustly support the claim (Page 6, Line 121) and clarified the terminology. In the context of our study, which did not conduct a formal clinical diagnosis, we are referring to insomnia symptoms. The text has been updated

*to precisely reflect this by stating "*insomnia symptoms*" (Page 6, Line 120).*

*Meanwhile, in response to this and previous comments regarding Line 227 (*acupoint massage is never introduced in the intro- what is the background for it. Why did you add this as an intervention?*), we have moved this entire sentence to the Introduction section (Page 6, Lines 119-121). This allows us to properly establish the background before presenting our own findings. The Discussion section is now more focused on interpreting the results of our specific study. We hope these changes have substantially strengthened the manuscript's clarity, scholarly rigor, and logical flow.*

Line 307: how was missing data handled? I expect this will be in the methods.

Response: *Thanks for raising this important methodological point. The handling of missing data was pre-specified in our Statistical Analysis Plan (SAP), which is detailed in Supplementary Appendix 4 (Supplementary Material, Pages 50-54, Appendix 4).*

1. For the primary outcomes (FFP and SPPB scores) in the Intention-to-Treat (ITT) analysis, missing values were imputed using the Expectation-Maximization (EM) algorithm with a single imputation approach and a fixed seed to ensure reproducibility, as stated in the SAP section Missing value imputation.
2. For the Per-Protocol Set (PPS) analysis, no imputation was performed for missing data. The PPS included only randomized participants who were compliant and had complete primary outcome data.
3. For time-to-event data (e.g., readmission, mortality), events occurring beyond the 1-year study period were censored, with the time imputed as 365 days.

*Following your suggestion, we have now explicitly added a summary of this missing data handling procedure to the Statistical Analysis section in the revised manuscript for clarity. The text reads: "*The handling of missing data was pre-specified. In the ITT analysis, missing values for the primary outcomes were imputed using the expectation-maximization method, with one imputation performed and a fixed seed to ensure reproducibility. In the PPS analysis, no imputation was performed for missing data.*" (Pages 23-24, Lines 558-562). We believe this revision ensures that our approach to managing missing data is transparent and readily accessible to the reader within the main methods section. Thank you for this valuable suggestion.*

Line 307: Also, you defined adherence as better than 50% adherence, but that is a low threshold. Why did you choose 50% and not a higher threshold, like 70 % or greater? The low adherence could certainly have biased the outcomes. See: https://www.jclinepi.com/article/S0895-4356(24)00366-4/fulltext

Response: *Thank you for this insightful comment and for pointing us to the highly relevant meta-epidemiological study by Páez et al. (2025). We agree that the choice of*

an adherence threshold is critical and that a higher threshold, such as 70%, might better reflect optimal intervention delivery, as suggested by the literature.

Our decision to employ a ≥50% threshold was guided by several methodological and pragmatic considerations specific to our trial:

（1）Real-World Pragmatism: A key aim was to assess effectiveness under conditions resembling clinical practice, where perfect adherence is often unattainable, especially in a frail elderly population with complex needs. The ≥50% threshold was designed to identify a cohort that received a meaningful minimum dose of the intervention, allowing us to contrast them with largely non-adherent participants and probe for a dose-response effect.

（2）Statistical Power for Sensitivity Analysis: The primary purpose of the Per-Protocol analysis was a sensitivity check of the Intention-to-Treat results. Applying a substantially higher threshold (e.g., ≥70%) would have drastically reduced the size of the adherent subgroup, compromising the statistical power and interpretability of this sensitivity analysis. The ≥50% threshold preserved a robust sample size for a meaningful comparison.

（3）Operational Definition of Engagement: We conceptualized the 50% mark as a pragmatic cut-off for substantive engagement with the intervention protocol, distinguishing it from minimal or non-adherence.

*We acknowledge that use of a single 50% adherence rate is relatively low, and we have explicitly recognized this as a study limitation in the manuscript (Page 15, Lines 357-358). Meanwhile we have incorporated the recommended literature as a reference in the revised manuscript (Page 15, Line 358; Ref 30). In direct response to your valuable suggestion, we now state in the Limitations section that "*Higher adherence thresholds are needed in future studies to clarify the dose-response relationship.*" (Page 15, Lines 358-359).*

Line 338: adherence to what? Please specify.

Response: *Thanks for your detailed reading and review. What we want to express is adherence to treatment and comprehensive interventions. The sentence has been revised as "*adherence to treatment and comprehensive intervention*" (Page 14, Lines 323-324).*

Line 341: At times you say adherence, at times you say compliance. Please use one consistently. These are often used interchangeably but have different connotations and can be misunderstood by different readers. I suggest you stay with the term "adherence" throughout.

Response: *Thanks very much for your valuable correction. We have now consistently used the term "*adherence*" throughout the entire manuscript to avoid any potential confusion and to maintain terminological precision. We appreciate the guidance on this matter.*

Lines 364-370: This section is repetitive. It feels as if you are repeating the last part of your discussion. This should be edited to avoid that.

Response: *Thanks for your professional advice. We have carefully reviewed this section and identified redundant content, which has now been deleted. Furthermore, we have conducted a thorough rereading of the entire manuscript to restructure the Discussion section, removing tangential or non-essential commentary not directly supported by the research findings. We sincerely appreciate your valuable suggestions.*

Methods:
Line 379: they affirm these for the implementation of the protocol and conduct of the trial? If so, please specify that.

Response: *Thanks for your insightful comment. We have revised the statement in the manuscript to specify that our affirmation refers to the implementation and conduct of the trial. The text now clearly states: "*The authors affirm adherence to the trial protocol during its implementation and conduct, ensuring accuracy and completeness. *" (Page 17, Lines 396-398).*

Lines 412-417: who increased the intensity, and how? How was this assessed or determined, and by whom? What constitutes a "safe level?"

Response:
Thank you for your valuable and insightful comments, which have allowed us to clarify the exercise prescription and safety monitoring procedures in the revised manuscript.
The exercise intensity was progressively adjusted by the supervising rehabilitation physicians. The progression was guided by a combination of: ①Subjective feedback from the patient regarding their perceived exertion and tolerance. ②Objective clinical assessment by the rehabilitation team during supervised sessions or follow-up consultations. A "safe level" was explicitly defined as the highest intensity not associated with pain, adverse symptoms (e.g., severe dyspnea, dizziness), or functional deterioration.

*We have revised the Methods section to incorporate these clarifications. The text now reads:"*The exercise intensity was progressively adjusted by the supervising rehabilitation physicians. The progression was guided by the patient's subjective tolerance and clinical observation for any adverse effects. The intensity remained at a 'safe level,' which was defined as the highest level not associated with pain, adverse symptoms, or functional deterioration.*" (Page 19, Lines 441-446).*

Lines 425-428. What is TCM/what does it stand for? Why did you choose that in particular? Also, please add citations for these. What is a TCM tongue diagnosis, and what does it or the pulse method diagnose?

Response: *Thanks for these important questions, which have allowed us to significantly improve the clarity and methodological transparency of the TCM components in our manuscript.*

*1. Definition and Rationale for TCM: We have now spelled out the acronym as "*Traditional Chinese Medicine (TCM)*" upon its first use in the Introduction section (Page 6, Line 119) and provided a clear rationale for its inclusion. The text states: "*Acupoint massage, derived from traditional Chinese medicine (TCM), is recognized as an effective complementary therapy for insomnia symptoms and constipation, which contributes to a higher quality of life for elderly CVD patients*" (Page 6, Lines 119-121), supported by relevant citations (Refs 9 and 10).*

2. TCM Diagnostic Methods: In the Methods section, we have added concise explanations of the TCM assessments:

（1）Tongue Diagnosis is described as providing visual information about the state of organ systems and overall health.

（2）Pulse Diagnosis is described as assessing circulatory function and the functional status of internal organs.

We clarify that these methods, performed by licensed TCM practitioners, were integrated to determine the individual's TCM syndrome pattern, which in turn guided the personalization of the acupoint massage prescription (Pages 19-20, Lines 461-469).

We believe these revisions provide the necessary context and scientific rationale for the inclusion of TCM in our multidisciplinary intervention, ensuring it is understandable and reproducible for a broad readership.

Lines 428-430: These should be described somewhere, even if in supplementary materials.

Response: *Thanks for your important comment. We agree that a detailed description of the acupoints is essential for the reproducibility of the study. We wish to clarify that a comprehensive description of the acupoints, including their anatomical locations has already been provided in the Supplementary Materials (Page 15, Supplementary Methods). Furthermore, to ensure this information is easily accessible to the reader, we have now added a direct reference to it in the main text within the Methods section: "*A detailed description of acupoint localization and massage procedure is provided in the Supplementary Materials (Appendix 1 and 2).*" (Page 20, Lines 472-474).*

Lines 441-442: Unified approach to what? Please specify.

Response: *Thanks for your pertinent comment. We agree that greater specificity is*

needed. The "unified approach" primarily refers to the consistent application of the standardized comprehensive multidisciplinary intervention protocols detailed in our study, ensuring that every patient received the same core intervention regardless of which team member was delivering it.

To clarify this in the manuscript, we have revised the text as follows:"<u>Team members received specialized training on the standardized comprehensive multidisciplinary intervention protocols to ensure a unified approach to patient management and effective collaboration.</u>*" (Page 21, Lines 488-490). We believe this revision eliminates ambiguity and clearly specifies the objective of our training and coordination efforts.*

Lines 449-453: see my comments about this in the discussion section comments, above.

Response: *Thanks for pointing us back to this important discussion. As detailed in our previous response above (Line 307), the choice of a ⩾50% adherence threshold was primarily driven by our study's focus on real-world effectiveness, the need for sufficient statistical power in sensitivity analyses, and the aim to define a minimum threshold for meaningful engagement.*
We fully acknowledge your concern that this relatively low threshold could have biased our outcomes. In direct response to this valid point, we have explicitly recognized the potential for bias due to the chosen adherence threshold as a study limitation in the Discussion section (Page 15, Lines 357-358). We also outlined our plan for future studies with higher adherence targets (e.g., ⩾70%) to further elucidate this relationship (Page 15, Lines 358-359).

We thank again for raising this critical point and for the invaluable guidance throughout the review process. We are grateful for the opportunity to have strengthened our manuscript in response to all comments. The feedback has been instrumental in enhancing the clarity, rigor, and overall quality of our work.

RESPONSE TO REVIEWERS

We sincerely thank the Editor for the opportunity to further revise our manuscript and for arranging an additional statistical review. We are particularly grateful to Reviewer #5 for the meticulous evaluation of the statistical aspects of our work. The insightful comments have guided us to substantially improve the methodological rigour, transparency, and reproducibility of our report.

In this revision, we have thoroughly addressed all remaining concerns. Key modifications include: (i) removal of statistical tests from baseline characteristic tables in accordance with best practices for randomised trials; (ii) precise reporting of adjusted effect estimates and clarification of analytical terminology throughout the manuscript; (iii) comprehensive revision of the fall event analysis, now presented as new falls per follow-up period with a cautious discussion of temporal patterns; (iv) expanded transparency regarding sample size calculations, subgroup analyses, and the proportional hazards assumption; and (v) incorporation of additional citations and clarifications regarding the Clinical Frailty Scale and Fried Frailty Phenotype operationalisation as requested by Reviewer #4.

We greatly appreciate all the reviewers for their thorough evaluation and thoughtful recommendations. Their expertise has helped us identify key areas for improvement, and we have carefully revised the manuscript in response to each point raised. The detailed responses are provided below. We are confident that these changes have markedly improved the quality and clarity of the work, and we hope the revised manuscript is now suitable for publication in *Nature Communications*.

We have provided two versions of the revised manuscript: one with tracked changes (Manuscript_Revised_with_Changes) and a clean version (Manuscript_Revised_Clean). The line numbers referenced here correspond to the clean version without revision marks.

Reviewer #2 :

Thank you for the clear responses - no further concerns.

RESPONSE:

Thanks for the positive acknowledgment and for confirming that no further concerns remain. We greatly appreciate your insightful comments on the previous version, which have substantially strengthened the manuscript.

Reviewer #3:

Dear authors,

Thank you for your very frank and thoughtful response to my questions and comments, as well as to the other reviwers'. These have improved the manuscript considerably and have addressed my concerns. In particular, the more nuanced and balanced discussion and conclusions now address my previous concerns. The reproducibility of the methods is also significantly improved.

RESPONSE:
Thanks for the positive and encouraging feedback. We are delighted to hear that our revisions, particularly the more nuanced discussion and conclusions as well as the improved methodological reproducibility, have addressed your previous concerns. We greatly appreciate your thoughtful and constructive comments on the earlier version, which have significantly enhanced the quality of our manuscript.

Reviewer #4:

As Reviewer 1 was unavailable, I have been asked to assay your replies. Here is how I see them, having first read the paper independently of any of the reviews.

Briefly this is an RCT of a truly novel multidimensional intervention: a set of posture, breathing, pelvic floor, and stretching/limb movements termed "rehabilitation exercises", patient education, nutritional guidance, acupoint massage, and polypharmacy management. It benefited in persuasive ways (moving the needle on the Fried Physical Frailty Phenotype, by the Short Physical Performance Battery score (SBBP), and a difficult-to-change readmission rate. Several points are of note here, including that the intervention was made to be culturally appropriate (the acupoint massage (self-administered) is based on traditional Chinese Medicine, with useful details in the Methods, and especially in the Supplementary Methods. Also, demonstrating benefits of polypharmacy is proving to be quite tricky, enough for achieving modest gains such as reducing anticholinergic burden.
Strikingly, at one year, the intervention group showed a lower degree of frailty than they had at the start by FPFP score, with a significant between-group difference favoring the intervention group. This is bolstered by better SPPB scores and by a similar Clinical Frailty Scale profile, together with superior hospitalization-free survival, but not with better survival overall.
These factors in an RCT evidence of a statistically significant, clinically detectable, biologically plausible impact, seen across multiple outcomes and converging with existing evidence, all point to the intervention making a clinically meaningful difference.

Points 1, 2 As it now stands, I have no concerns about the description of how participants were recruited, or how the data were analysed. It appears that the Reviewer's comments were spot on, and indeed the paper has improved as a result. It's especially reassuring to know too that the subgroups analyses were considered a priori and not post hoc.

Point 3. If Reviewer One, by "adjusted effects size" meant something along the lines of Standardized Response Means, then constructing 95% confidence intervals for the differences is not exactly the same. A standardized response mean (or Hedge's G or Cohen's d) as an estimate of effect size tells you the size of the effect. The area is famously controversial. Here is a detailed guide. Ellis PD.(2012) The Essential Guide to Effect Sizes. Cambridge University Press ISBN:

9780511761676 DOI: https://doi.org/10.1017/CBO9780511761676 Nat Communs likely has a statistical reviewer who can point to an acceptable answer. (Beware though: the most vitriolic review I've ever had came from an aggrieved statistical reviewer over which effect size was most appropriate.)
Points 4 and 5: To me, these read as variants of the standard critique that RCTs might be valid, but not generalizable. In my view, the authors' answers are entirely appropriate.
The points covered from pages 6-21 indicate a very careful read, and equally careful and unobjectionable replies, in my estimation. The single objection is the issue around whether one can ever say that a patient is becoming more frailer (or frailer). In my experience, it is indubitable that people can become frailer – and your data show that they can become less frail too. I respect that, wisely in my view, you elided the issue while still making clear that you employ quantitative reasoning in making your comment – in relation to the differences in the mean number of frailty items present in the two groups. .

This leads me I'd make one other comment. I paid attention that the other secondary measures included the Clinical Frailty Scale, and that it too showed significant improvement. (Let me declare here that I'm Kenneth Rockwood, the originator of the CFS.) I'd be inclined to highlight this finding in the Discussion, Here's why. The CFS is derived from the deficit accumulation approach to frailty, also introduced in 2001. The field is often said to be "plagued" by having the two definitions. Here, you can join the comparatively small number of papers that report interventions with estimates in relation to both measures.

I also note though that the Clinical Frailty Scale, as with all the other outcomes measures, save the Fried Physical Frailty Phenotype, goes uncited. Given that there are hundreds of variation of the Fried Physical Frailty Phenotype (PMID: 25846451) it would be useful to know exactly how it was operationalised here. As far as I can see, the only modification was, by inference from the Statistical Analysis Plan (Appendix 3, p44 and for example, Table 1, main paper, p34, where it is reported as a score with mean ± SD,) was that for analytic purposes, it was treated as a six-point frailty index (that is to say, from zero to five features). This contrasts with the 2001 report (your ref 2) where it is

presented as a categorical variable. To avoid confusion, here is the version of the CFS that the authors appear to have used: Rockwood K, Theou O. Using the Clinical Frailty Scale in Allocating Scarce Health Care Resources. Can Geriatr J. 2020 Sep 1;23(3):210-215. doi: 10.5770/cgj.23.463. PMID: 32904824; PMCID: PMC7458601.

Kenneth Rockwood, Halifax, Canada

RESPONSE:
We express our sincere gratitude to you for your invaluable insights and for the time you have devoted to reviewing our manuscript. We are especially honoured that you, the originator of the Clinical Frailty Scale (CFS) has provided such a thoughtful assessment of our work. Your pioneering work on the deficit accumulation model of frailty has fundamentally shaped the field, and we are grateful for your guidance.

We are delighted that you recognise the novelty of our multidimensional RENAP intervention and find the results persuasive. We also appreciate your acknowledgment that the intervention was designed to be culturally appropriate, including the self-administered acupoint massage based on traditional Chinese medicine.

Thank you for your insightful comment regarding the challenges inherent in demonstrating benefits from polypharmacy management. We fully agree that proving the benefits of polypharmacy interventions alone is quite difficult. This observation further underscores the value of our integrated multidisciplinary RENAP approach, which combines polypharmacy management with rehabilitation exercises, nutritional guidance, patient education, and acupoint massage to achieve synergistic effects on frailty and clinical outcomes.

We also appreciate your observation that the benefits are seen across multiple outcomes, including the FFP, SPPB, CFS, and hospitalisation-free survival. This convergence, together with the pre-specified subgroup analyses, supports our conclusion that the RENAP intervention produces clinically meaningful improvements.

In addition to your overall comments, we have carefully considered each of the specific points you raised. Our responses are as follows.

Points 1-2: Recruitment and data analysis

Response*: Thanks for confirming that you have no concerns about our recruitment description or data analysis, and for noting that our subgroup analyses were pre-specified rather than post hoc.*

Point 3: Effect size reporting

Response: *Thank you for this important methodological clarification and for directing our attention to the relevant literature on this topic, including the detailed guide by Ellis (2012). We are grateful for your guidance in navigating this nuanced area.*

You are correct that the between-group adjusted mean differences with 95% confidence intervals we reported are absolute effect sizes, which differ from standardised effect sizes such as Cohen's d or Hedge's g. Our reporting follows the CONSORT recommendation for RCTs, which emphasises presenting adjusted between-group differences with confidence intervals.

We also acknowledge your point that the choice of standardised effect size can be a matter of ongoing discussion. In light of your advice, should the journal's statistical reviewer deem it necessary to report standardised effect sizes, we would be happy to calculate and report Cohen's d (or Hedge's g, as appropriate) for the primary outcomes. We would respectfully defer to the statistical reviewer's guidance on this matter.

Once again, we appreciate your thoughtful advice and your kind warning regarding the potential pitfalls in this area.

Points 4 and 5: Generalizability

Response: *Thanks for acknowledging that our responses to these standard critiques of RCT generalizability were appropriate. We have made no further changes on these points.*

Language of frailty change:

Response: *Thank you for your thoughtful observation on this point. We are grateful that you recognise our effort to avoid potential linguistic ambiguity by relying on quantitative comparisons of the mean number of frailty criteria between groups. Meanwhile, taking into account the subsequent suggestion from Reviewer 5 regarding sample size calculation, we have further revised the description in the manuscript.*

CFS findings in Discussion

Response: *We fully agree with your suggestion that the significant improvement observed in the CFS deserves greater emphasis in the Discussion. This convergence across two complementary frailty frameworks strengthens the evidence for the clinical meaningfulness of the RENAP intervention. The frailty field has long been described as being "plagued" by the coexistence of two distinct conceptual frameworks-the phenotype-based model (Fried) and the deficit accumulation model (CFS). Both have proven valuable, yet studies reporting intervention effects using both measures simultaneously remain comparatively rare.*

*Following your suggestion, we have added a paragraph to the Discussion section as follows: "*Our research suggests that the multidisciplinary RENAP approach may contribute to meaningful improvements in frailty, as measured by FFP score. Notably, the CFS score, a measure derived from the deficit-accumulation approach to frailty, was also significantly improved. It is worth noting that the FFP and CFS represent two conceptually distinct approaches to defining frailty: the former is based on a specific phenotype, while the latter relies on the accumulation of deficits across multiple domains. Despite these conceptual differences, both showed convergent improvement following the RENAP intervention. To our knowledge, limited intervention studies have reported simultaneous benefits across both of these frailty measures. This convergence across two complementary frailty frameworks strengthens the evidence that the RENAP intervention produces meaningful, multidomain enhancements in muscle strength, physical endurance, and overall functionality.*" (Pages 11-12, Lines 244-255) .*
We are deeply honoured by your suggestion and believe this addition substantially strengthens the Discussion by highlighting the convergent validity of the intervention effects across both frailty paradigms.

CFS citation and version

Response*: We sincerely apologise for omitting the citation for the CFS. We recognise that this was a significant oversight, especially given that you are the originator of this important scale, which has made a profound impact on frailty research and clinical practice worldwide. Your development of the CFS has provided the field with a practical, clinically meaningful tool for assessing frailty, and we are grateful for your contributions.*

We have now added the recommended citation to the manuscript: Rockwood K, Theou O. Using the Clinical Frailty Scale in Allocating Scarce Health Care Resources. Can Geriatr J. 2020;23(3):210-215. This reference has been included in the Methods section where the CFS is formally defined as a secondary outcome measure (Page 24, Line 553), as well as in the reference list (Page 31, reference 34).

We confirm that the version of the CFS we used is indeed the 9-point scale as described in that paper, ranging from 1 (very fit) to 9 (terminally ill). For analytical purposes, we treated the CFS score as a continuous variable, consistent with common practice in frailty research.

FFP operationalisation

Response*: We agree that it is important to specify exactly how the FFP was operationalised in our study. We have revised the Methods section to explicitly it (Pages 23-24, Lines 546-549). Specifically, each of the five criteria was scored as 0 (absent) or 1 (present), yielding a total score from 0 to 5. For primary analysis, the FFP score was*

treated as a continuous variable, which allowed us to detect changes in frailty severity beyond the binary classification of frail versus non-frail. Frailty was defined as a score of ≥3 for inclusion criteria, consistent with the original CHS definition (reference 2). We acknowledge that this analytic approach treats the FFP as a continuous measure rather than a categorical variable, which we have now clarified in the manuscript (Pages 23-24, Lines 547-549).

We are deeply grateful for your time, expertise, and constructive guidance. Your insights have significantly strengthened our manuscript, and we appreciate your pioneering work that has shaped the field of frailty research. Please accept our sincere apologies once again for the omission.

Reviewer #5:

This review considers the use of statistics in the paper by Chai et al, a randomised controlled trial of a multicomponent intervention for frail elderly patients with CVD.

Overall, this appears to be a well designed and conducted trial. Generally, the statistical methods are good, and everything is quite well described and presented. I do have a few observations, which should be easily remedied. The following is ordered according to how I encountered things in the paper.

Regarding the randomisation, I would like to see a little more detail about blocking and stratification, if these were done. Also, can the authors be sure that the allocations were effectively concealed, so that those recruiting participants were unaware of what the next allocation was going to be?

The results section begins with a lengthy section about the balance of the baseline data. For one thing, it is generally not advised to report statistical tests of baseline data (given the randomisation, it is known that the null hypothesis is true). In this case, it would be sufficient to state that there were no major differences between the groups, with reference to the table. It is not necessary to list all the baseline factors which were looked at. And, I would advise against reporting statistical results as “p>0.05”; there is a world of difference between p=0.06 and p=0.99.

When looking at the statistical comparisons in Table 1, I was not able to replicate the p-value for BMI (I got t=1.56, p=0.119), assuming a standard t-test was done, and given the means and SDs reported. I was not expecting an exact match, due to rounding of the summary data reported, but this is very different. Also, for age, I got p=0.242 (this time, a smaller p-value). So, whilst I do not recommend statistical tests of baseline data, I would suggest these results need to be checked.

I then looked as the categorical variables. The footnote to the table reports these were compared with chi-square tests, whereas the methods section of the paper also mentions Fisher tests. Looking at a couple of p-values, these all seem to be Fisher test p-values. This is fine – I would routinely use Fisher tests rather than chi-square. If this is what was done, the statistical methods and table footnotes should reflect this.

Line 162, should this be reported as an “adjusted mean difference”, rather than simply “mean difference”?

The PPS is defined as those completing the intervention and having outcome

data, but what does “completing the intervention” mean for the control arm?

Line 176, I think the control group showed an increase of 0.2 points, not a decrease.

It is probably worth noting that the analysis of mortality had very little power, and whilst the confidence interval is very wide, the point estimate is the same as for hospitalisations. Simply saying that it was not statistically significant is perhaps a little down-beat.

I note the slightly higher fall rate in the intervention arm, perhaps not unexpected if the intervention leads to greater mobility. Did any falls lead to hospitalisations?

For the subgroup analyses, it is not sufficient to simply conduct the analyses within subgroups and comment on which analyses were statistically significant or not. It is better to fit regression models with interaction terms to assess whether there is evidence that the intervention effect differs between subgroups. Only if the test of interaction is significant (some people use a more relaxed $p<0.1$ when looking at interactions) does it make sense to comment on how the effects differ between groups. I would include a column of interaction p-values to the right of Figure 3.

For the sample size calculations, in order to be able to replicate the calculations, the reader needs the assumed means and standard deviations (or mean difference and common standard deviation). These should be provided with reference(s) to justify.

When fitting Cox models, one should check the proportional hazards assumption. Was this done?

Line 576: “multivariable” is more correct.

RESPONSE:

Thanks for your thorough and insightful review of the statistical aspects of our manuscript. We are grateful that you have recognised the overall quality of the trial design and the clarity of our statistical presentation. Your detailed observations, which we address point-by-point below, have been invaluable in further improving the methodological rigor and transparency of our work. We appreciate the time and expertise you have devoted to this review.

Comment 1: more detail about blocking and stratification.

Response*: Thank you for this question. The randomisation was performed using a*

simple randomisation scheme without stratification. The allocation sequence was generated by a central computer system within the EDC, with a 1:1 allocation ratio. No blocking or stratification was applied.

We have added a brief clarification to the Randomisation and blinding section of the manuscript (Page 20, Lines 452-455) to reflect these details.

Comment 2: can the authors be sure that the allocations were effectively concealed, so that those recruiting participants were unaware of what the next allocation was going to be?

Response*: Thank you for this important question.*

We can confirm that allocation concealment was effectively ensured. The randomisation sequence was generated and maintained by the central computer system within the EDC using a simple randomisation scheme with a 1:1 ratio, without blocking or fixed patterns. The group assignment was only revealed after the participant had been fully enrolled, had provided written informed consent, and had completed all baseline assessments. The research staff responsible for participant recruitment and baseline assessment had no access to the upcoming allocation sequence.

Critically, because the allocation sequence was truly random (simple randomisation) rather than following any predictable pattern, the next assignment was statistically unpredictable. Even if a researcher had observed previous allocations, they could not infer the next treatment assignment. This, combined with the centralised web-based system, provides robust allocation concealment.

*In response to your comment, we have added the following clarification to the Methods section (Page 20, Lines 455-458): "*The randomisation sequence was generated to ensure no manual interference. The allocation sequence was statistically unpredictable, and group assignment was only revealed after participant enrolment, informed consent, and baseline assessments were completed.*"*

We believe this addition improves the transparency of our randomisation procedures. We appreciate your careful attention to this methodological detail.

Comment 3: results section, begins with a lengthy section about the balance of the baseline data.

Response*: Thanks for this important methodological guidance. We agree that formal statistical testing of baseline characteristics is generally not recommended for randomised controlled trials (RCT), and that reporting "P > 0.05" as a single category is imprecise. Accordingly, we have revised the manuscript as follows:*

First, the Results section has been streamlined. Instead of listing individual baseline variables with their P values, we now simply state the enrollment number and demographic characteristics, with reference to Table 1. The statistical results has been removed.

Second, the P value and test statistic columns have been removed from Table 1. The table now presents only the descriptive statistics for the intervention and control groups, without comparative statistical testing.

In addition to revising Table 1 as recommended, we have also removed the P value and test statistic columns from Supplementary Table 1 (baseline characteristics of the PPS population) to maintain consistency with the main table and to avoid unnecessary statistical testing of baseline variables.

We believe these revisions align the manuscript with current best practices for RCT reporting and thank you again for these constructive suggestions.

Comment 4: When looking at the statistical comparisons in Table 1, I was not able to replicate the p-value for BMI (I got t=1.56, p=0.119), assuming a standard t-test was done, and given the means and SDs reported. I was not expecting an exact match, due to rounding of the summary data reported, but this is very different. Also, for age, I got p=0.242 (this time, a smaller p-value). So, whilst I do not recommend statistical tests of baseline data, I would suggest these results need to be checked.

***Response**: Thank you for the meticulous verification of our baseline data. You correctly identified a discrepancy between the p-value that would be obtained from a standard t-test using the reported means and standard deviations (approximately p=0.119 for BMI) and the p-value we reported (p=0.015).*

This discrepancy arises because we used the Wilcoxon rank-sum test for comparing BMI and age, rather than the t-test. The Wilcoxon test was chosen because the distribution of several baseline continuous variables deviated from normality, as assessed by the Shapiro-Wilk test We apologize for this statistical inconsistency..

Nevertheless, we fully agree with your primary and more critical point: statistical testing of baseline characteristics in a RCT is generally not recommended. Randomisation ensures that any observed differences are due to chance. Conducting and reporting these tests can be misleading. Therefore, to address your concern and to adhere to current best practice, we have taken the following actions as previously described:

Removed the p-value and test statistic columns entirely from Table 1.

We are very grateful for this insightful comment, which has led us to substantially improve the methodological rigor and clarity of our manuscript. We apologise again for any confusion our original presentation may have caused.

Comment 5: Categorical variable comparisons

***Response**: Thanks for this careful observation. We agree that both chi-square tests and Fisher's exact tests were used for categorical variables in Table 1, depending on the expected cell frequencies. Specifically, Fisher's exact test was applied when any cell had an expected frequency less than 5; otherwise, the chi-square test was used.*

As noted previously, we have removed the P value column along with the corresponding test statistics from Table 1 in response to your earlier recommendation that formal statistical testing of baseline characteristics is not warranted for RCTs. Consequently, Table 1 now presents only descriptive statistics for the two groups, and the issue of whether chi-square tests or Fisher's exact tests were used for baseline categorical comparisons is no longer applicable, as these results are no longer reported. We appreciate the meticulous attention to detail.

Comment 6: adjusted mean difference

***Response**: Thank you for this important terminological clarification. You are correct. The between-group differences for all primary and secondary outcomes were estimated from mixed-effects models that adjusted for baseline outcome values and centre effects, as described in the Statistical analysis section. Therefore, these should be reported as "adjusted mean differences" rather than unadjusted "mean differences." This applies to both the ITT and PPS analyses, as well as the Abstract and the supplementary material.*

We have revised the manuscript accordingly at the following locations:
Abstract (Page 5, Lines 88-89):Two instances of "mean difference" were changed to "adjusted mean difference"

Results section (Pages 8-9):
Lines 156-157: "between-group mean difference" → "between-group adjusted mean difference"
Line 160: "between-group mean difference" → "between-group adjusted mean difference"
Line 165: "between-group difference" → "between-group adjusted difference"
Line 173: "between-group mean difference" (CFS) → "between-group adjusted mean difference"
Line 176: "between-group mean difference" (IADL) → "between-group adjusted mean difference"

Lines 179-180: "between-group mean difference" (HADS-A) → "between-group adjusted mean difference"

Table 2 (Page 38):
Table 2 title: "Estimated Between-group Mean Change Differences" → "Estimated Between-group Adjusted Mean Change Differences"
Table 2 column header: "Estimated difference in mean change at 1-year (95% CI)" → "Estimated adjusted difference in mean change at 1-year (95% CI)"
Table 2 footnote: "estimated difference in mean change" → "estimated adjusted difference in mean change"

The corresponding content in the supplementary material has also been revised.

We appreciate your meticulous attention to this terminological nuance, which enhances the precision our reporting across the entire manuscript.

Comment 7: Definition of PPS for the control arm

***Response**: Thank you for this important clarification. You are correct that the phrase "completing the intervention" in the Results section does not apply appropriately to the control arm.*

To address this, we have taken the following approach: the Results section now simply states the sample sizes for the PPS analysis (n=120 in the intervention group, n=132 in the control group) without repeating the definition, as the full definition is provided in the Methods section. The ambiguous phrase "completing the intervention" has been removed.

We have also revised the definition of the Per-Protocol Set in the Statistical analysis section (Page 24-25, Lines 572-579) to provide clear, parallel criteria for both arms:
"The PPS was a subset of the ITT, comprising participants who completed the study per protocol. For the intervention group, this required completing at least 50% of the prescribed RENAP components (rehabilitation exercises, nutritional guidance, and acupoint massage) and having no missing primary outcome data. For the control group, this required receiving standard medical management without crossover to the RENAP intervention, attending all scheduled follow-up assessments, and having no missing primary outcome data."

We believe this approach eliminates the ambiguity while keeping the Results section concise. We appreciate your careful reading and thoughtful guidance.

Comment 8: CFS change direction for the control group

***Response**: Thank you for catching this error. You are absolutely correct. According to*

the data in Table 2, the control group showed an increase of 0.2 points in the CFS score (from 4.8 at baseline to 5.0 at 1-year follow-up), not a decrease. We apologise for this mistake.

We have revised the sentence in the Results section (Page 8, Lines 171-172) as follows: "The mean Clinical Frailty Scale (CFS) score decreased by 0.4 points in the intervention group compared to an increase of 0.2 points in the control group."

We appreciate your meticulous attention to detail, which has corrected an important inaccuracy in our manuscript.

Comment 9: Reporting of mortality results

***Response**: Thanks for this thoughtful and constructive suggestion. You are absolutely correct. Simply stating that the mortality result was "not statistically significant" fails to convey the full picture and may be overly pessimistic.*

As you note, the point estimate for mortality (HR = 0.53) is identical to that for readmissions, which did reach statistical significance. However, the mortality analysis was severely underpowered due to the small number of events (only 15 deaths in total), resulting in a very wide confidence interval (0.18 to 1.55) that is compatible with both a clinically meaningful benefit and no effect.

We have revised the manuscript accordingly:

Results section (Page 9, Lines 190-194) now reads:
"Regarding mortality during the 1-year follow-up, 5 patients (3.0%) in the intervention group and 10 patients (6.0%) in the control group experienced all-cause mortality (P = 0.237 in the log-rank test, Figure 2D). Cox regression analysis yielded a HR of 0.53 (95% CI: 0.18 to 1.55, P = 0.286, Supplementary Table 6) and the PPS analysis also did not indicate statistical significance (Supplementary Figure 1D)."

Discussion section (Page 13, Lines 275-283) has been expanded to provide the interpretative context: "Although mortality was lower in the intervention group, the difference did not reach statistical significance, likely due to the small number of events (n=15) and consequent low statistical power. Notably, the point estimate for mortality (HR = 0.53) was identical to that for hospital readmissions, which did show a statistically significant benefit. However, the wide confidence interval (0.18 to 1.55) is consistent with both a clinically meaningful protective effect and no effect. Therefore, no firm conclusion can be drawn regarding mortality from this study, and future adequately powered trials using a larger sample size are needed to address this question."

We appreciate your guidance in providing a more nuanced and informative presentation of these results.

Comment 10: Fall events

***Response**: Thank you for this insightful observation. You astutely noted that the different fall rates in the two groups might reflect the potential impact of the intervention on patients,, a comment that we found highly instructive and valuable. In response to your comment, we have re-analysed the fall data and revised the manuscript accordingly.*

Instead of reporting a single overall fall incidence (which was based on an erroneous calculation and has been removed), we now report new falls occurring during each follow-up period (0-3, 3-6, and 6-12 months). The revised data are presented in Supplementary Table 7. At each individual time point, there were no statistically significant differences in fall incidence between the two groups. However, an exploratory inspection of the temporal pattern revealed that the intervention group experienced more new falls during the first six months (20 vs. 16) but fewer during the 6-12 month period (8 vs. 13). This observation raises the possibility that the RENAP intervention might be associated with an initial increase in fall risk, possibly due to increased mobility, followed by a potential protective effect in the longer term, which could reflect improved balance and physical function.

We have also reviewed fall-related hospitalisations. In the intervention group, 1 out of 28 falls led to hospitalisation. In the control group, 4 out of 29 falls led to hospitalisation, involving three participants (one participant experienced two such events). All fall-related hospitalisations were non-fatal.

*In the Results section, we have replaced the previous text as follows: "*the number of new falls occurring during each follow-up period was shown in Supplementary Table 7. At each time point, there were no statistically significant differences between the two groups (0-3 months: P=0.715; 3-6 months: P=0.294; 6-12 months: P=0.313). Fall-related hospitalisations occurred in one participant in the intervention group and in three participants in the control group. All fall-related hospitalisations were non-fatal.*" (Pages 9-10 , Lines 195-200).*

*In the Discussion section, we have added a paragraph interpreting the temporal pattern with appropriately cautious language: "*Regarding safety, the temporal pattern of falls deserves comment. At each individual time point, there were no statistically significant differences in fall incidence between the two groups. However, an exploratory inspection of the data revealed that the intervention group experienced more new falls during the first six months but fewer during the 6-12 month period. This pattern raises the possibility that the RENAP intervention might be associated

<u>with an initial increase in fall risk, potentially due to increased mobility, followed by a longer-term trend toward fewer falls, which could reflect improved balance and physical function. Nevertheless, given the small numbers and the lack of statistical significance at individual time points, these observations are hypothesis-generating only and should be interpreted with caution.</u>" *(Page 13, Lines 284-294).*

We appreciate your insightful suggestion, which has allowed us to provide a more nuanced and rigorous analysis of the intervention's safety profile.

Comment 11: Subgroup analyses and interaction tests

Response*: Thank you for this important methodological suggestion.*

We would like to clarify our original intention for the subgroup analyses. Our primary goal was to assess whether the beneficial effect of the RENAP intervention was consistent across clinically relevant subgroups, rather than to directly compare treatment effects between subgroups. The subgroup factors were prespecified based on clinical judgment as potentially prognostic factors, not selected through data-driven or post-hoc approaches. The purpose of these subgroup analyses was to provide a descriptive and exploratory assessment of the treatment effect within clinically relevant subgroups, rather than to formally identify effect modification or to make inferential comparisons between subgroups. As shown in Figure 3 and Supplementary Figure 2, the intervention consistently favoured the RENAP group across nearly all subgroups, with point estimates all in the same direction and no evidence of effect reversal.

We fully acknowledge your point that formal interaction tests provide a more rigorous statistical assessment of whether treatment effects differ between subgroups. However, our original analysis plan did not include interaction tests, as our focus was on demonstrating within-subgroup consistency rather than between-subgroup heterogeneity. Given the exploratory nature of these subgroup analyses and the fact that they were pre-specified based on clinical rationale, we respectfully note that the absence of interaction tests does not undermine the main finding that the RENAP intervention was broadly effective across diverse patient subgroups.

Nevertheless, we recognise the value of your suggestion and will consider including interaction tests in future studies designed with sufficient power for such comparisons. We have added a statement to the Limitations section acknowledging that formal interaction tests were not performed and these subgroup analyses were descriptive in nature and should not be interpreted as evidence of differential treatment effects between subgroups.

In response to your suggestion, we have revised the manuscript as follows:

Methods section (Page 26, Lines 611-616): "Subgroup analyses were conducted to assess whether the beneficial effect of the RENAP intervention was consistently observed across clinically relevant subgroups. The pre-specified subgroups were chosen based on a priori clinical relevance rather than post-hoc data exploration, including sex, age (≥80, <80 years), body mass index (BMI, >24, 18 ≤ BMI ≤ 24), heart failure, coronary artery disease, atrial fibrillation/flutter, and diabetes."

Results section (Page 10, Lines 202-206): "Subgroup analyses were presented in Figure 3 (Figure 3A for FFP and Figure 3B for SPPB). For all subgroups, the point estimates for the between-group adjusted mean differences favoured the RENAP group for both FFP and SPPB. For all-cause readmission, the hazard ratios favoured the intervention group across most subgroups (Supplementary Figure 2)."

Discussion section (Page 18, Lines 399-404): "Subgroup analyses were pre-specified and intended to assess the consistency of the intervention effect across clinically relevant subgroups. Formal interaction tests were not performed, as these analyses were exploratory in nature and the study was not powered for between-subgroup comparisons. Therefore, the subgroup findings should be interpreted as descriptive and hypothesis-generating rather than confirmatory."

We believe these revisions clarify the intent and scope of our subgroup analyses while maintaining transparency about their exploratory nature. We appreciate your guidance on this matter, which has helped us better articulate the purpose and limitations of our subgroup analyses. We fully acknowledge that interaction tests are the gold standard for assessing effect modification. While our study was not powered for such analyses, this important suggestion will guide the design and statistical planning of our future, larger-scale trials to formally investigate potential heterogeneity of treatment effects.

Comment 12: Sample size calculation transparency

Response*: Thank you for this important methodological point. To enable replication of the sample size calculation, we have revised the Statistical analysis section to clearly report the assumed parameters with reference to the original sources.*

For the Fried Frailty Phenotype (FFP) score, we referenced both Cameron et al. (2013, reference 35) and Ng et al. (2015, reference 36). Cameron et al. reported a mean reduction of 0.80 points (SD 1.19) in the intervention group and 0.41 points (SD 1.02) in the control group at 12 months, corresponding to a between-group mean difference of 0.41 points. Ng et al. (2015) reported a between-group difference in frailty score reduction of approximately 0.4 to 0.6 points favoring the intervention groups, which is consistent with the effect size reported by Cameron et al. (2013). Based on these data, we assumed a between-group difference of 0.39 points with a common standard deviation of 1.1. Using a two-sided two-sample t-test (80% power,

α = 0.05), approximately 250 participants (125 per group) were required.

For the Short Physical Performance Battery (SPPB) score, Cameron et al. reported a mean increase of 0.52 points (SD 2.47) in the intervention group and a mean decrease of -0.98 points (SD 2.30) in the control group at 12 months, corresponding to a between-group mean difference of 1.4 points. Assuming a common standard deviation of 2.4, approximately 94 participants (47 per group) were required.

The larger of these estimates (250 participants) was used as the basis for our sample size. Considering an anticipated attrition rate of 20%, the target sample size was set at 320 participants (160 per group), which was successfully recruited.

*We have added the assumed standard deviations to the manuscript and cited the original sources as follows: "*Based on Cameron et al. and Ng et al.,[35, 36] we assumed a between-group mean difference of 0.39 points in the FFP score reduction, with a common standard deviation of 1.10, requiring 250 participants (125 per group). For the SPPB score, we assumed a between-group mean difference of 1.40 points, with a common standard deviation of 2.40,[35] requiring 94 participants (47 per group). The larger of these estimates (250 participants) was used. Considering an attrition rate of 20%, a minimum of 320 participants was recruited.*" (Page 24, Lines 564-570). We appreciate your guidance in improving the transparency of our methods.*

Comment 13: Proportional hazards assumption for Cox models
When fitting Cox models, one should check the proportional hazards assumption. Was this done?

***Response**: Thank you for this important comment.*

We assessed the proportional hazards assumption using Schoenfeld residual-based methods (supremum test) implemented in SAS (PROC PHREG with the ASSESS statement). The results indicated that the proportional hazards assumption was generally satisfied for the treatment variable (RANDGRD, P = 0.304) and most covariates.

Although a statistically significant deviation was observed for one category of the NYHA classification variable, this is likely due to sparse data and instability in that subgroup. Importantly, this deviation does not materially affect the estimation of the treatment effect for RANDGRD, which remains the primary focus of our analysis.

Overall, there was no evidence suggesting violation of the proportional hazards assumption for the primary exposure of interest. In response to your comment, we have added a statement to the Methods section (Page 26, Lines 608-610) as follows: "The proportional hazards assumption was assessed using Schoenfeld residual-based methods and was satisfied for the treatment variable."

We believe this addition improves the transparency and rigor of our statistical reporting. We appreciate your careful review and constructive suggestion.

Comment 14: Line 576: “multivariable” is more correct.

***Response**: Thank you for this clarification. You are correct. The Cox regression model included multiple covariates to adjust for potential confounding, but only one outcome (all-cause readmission or mortality). Therefore, "multivariable" is the appropriate term. We have revised the manuscript accordingly at Line 618 in the revised manuscript, changing "multivariate" to "*multivariable*".*

We appreciate your meticulous attention to this detail. We extend our sincere gratitude for your expert statistical review. Your careful scrutiny and thoughtful recommendations have substantially enhanced the overall quality of our manuscript.

Response to Reviewer #5

We sincerely thank you for your thoughtful comments on our manuscript, and for your positive acknowledgment of our previous revisions. We greatly appreciate the time you have dedicated to reviewing our work. Below we address each of your comments point by point.

Comment 1:

"I thanks the authors for their careful consideration of my original comments. I am satisfied with all of their responses and have no further comments to make."

Response: *Thank you for the positive feedback and for your recognition of our previous revisions. It is an honour to receive your approval, and we thank you once again for your valuable input, which has helped improve our manuscript.*

Comment 2:

"Regarding a comment from another reviewer, I prefer to see effect estimates (or 'effect sizes') reported on the original scale (as in this paper), rather than being standardised, though I do appreciate the worth of standardised versions in some situations."

Response*: Thanks for sharing your methodological perspective. We have carefully re-examined our entire manuscript and confirm that all effect estimates (e.g., mean differences for FFP, SPPB, IADL, CFS, HADS-A; hazard ratios for readmission and mortality) are reported on the original scale, as preferred. No standardised effect sizes appear anywhere in the paper. We appreciate your note that standardised versions may be useful in certain contexts, and we will keep this in mind for future studies, as appropriate.*

Comment 3:

"I am not a SAS user."

Response: *Thank you for pointing this out. We fully understand that SAS may not be*

the preferred platform for all readers, and we appreciate your effort to review our code.

To ensure transparency, we have uploaded the complete SAS code used for this study to Zenodo and made it publicly accessible to reflect the full analytical process.

Meanwhile, we have provided a detailed and step-by-step description of our analytical methods in the manuscript, covering all key aspects including data processing, variable definitions, model specifications, and sensitivity analyses. We believe that, taken together, the publicly available code and the thorough methodological description in the manuscript jointly ensure the traceability of our analytical approach.

The code repository is available at https://doi.org/10.5281/zenodo.21026032. *We hope this adequately addresses the reviewer's concern, and we remain happy to provide additional clarification if needed.*

Once again, thank you for your rigorous and insightful review. Your guidance has been instrumental in strengthening this manuscript, and we are sincerely grateful for your continued support.
